# Evaluation Frameworks for Clinical AI Incorporating Validation Strategies, Real-World Applicability, and Ethical Principles: Scoping Review

**DOI:** 10.2196/78168

**Published:** 2026-07-22

**Authors:** Diana Carolina López Medina, Aida Oliveros-Navarro, Nataly Moreno Angel, Andrés Camilo Herrera- Arellano, Marcela Henao-Pérez

**Affiliations:** 1 Research Grupo Infettare, School of Medicine, Universidad Cooperativa Colombia, Calle 50, No 40-74, Bloque A, Medellín, Antioquia, 050012, Colombia, 57 3108406678; 2 Internal Medicine Residency Program, School of Medicine, Universidad Cooperativa de Colombia, Medellín, Colombia; 3 Medicine Program, School of Medicine, Universidad Cooperativa de Colombia, Medellín, Colombia

**Keywords:** artificial intelligence, clinical decision support systems, biomedical technology assessment, bioethics, validation studies as topic, scoping reviews, machine learning, evidence-based practice

## Abstract

**Background:**

AI shows substantial potential in health care; however, the absence of standardized evaluation frameworks limits its safe and effective clinical implementation because of inconsistent validation requirements and fragmented ethical principles. Existing guidelines vary in structure, methodological rigor, and ethical integration, creating uncertainty.

**Objective:**

This study aimed to systematically map, characterize, and critically analyze existing evaluation frameworks for clinical AI, focusing on three core dimensions: methodological rigor, validation strategies (internal validation, including reporting of technical and clinical performance; external validation, including real-world applicability), and alignment with the United Nations Educational, Scientific and Cultural Organization (UNESCO) AI ethical considerations.

**Methods:**

A scoping review was conducted following PRISMA-ScR (Preferred Reporting Items for Systematic Reviews and Meta-Analyses extension for Scoping Reviews) guidelines. Six databases (PubMed, Embase, BVS, EBSCOhost, ProQuest, and Sage) and the Enhancing the Quality and Transparency of Health Research Network were searched without language or date restrictions up to February 2026. Eligible documents included peer-reviewed papers, gray literature, and organizational guidelines describing evaluation or reporting frameworks for clinical AI. Editorials, commentaries, and conference abstracts lacking a clearly defined evaluative framework or clinical applicability were excluded. Two reviewers independently screened records and extracted data. Data were extracted across three domains: (1) general characteristics, (2) methodological rigor and validation parameters, and (3) ethical integration and were synthesized using a dot plot–based gap map. Ethical adherence was assessed using a 10-domain UNESCO-based scoring matrix. No formal risk-of-bias assessment was conducted, consistent with scoping review methodology.

**Results:**

From 3363 records, 46 frameworks met the inclusion criteria. Mapping revealed a rapidly expanding but fragmented landscape. Most frameworks targeted investigational use (88%), with limited focus on clinical applicability. Frameworks varied in structure, methodology, and scope, with a predominance of reporting guidelines and few validated tools. Most (63%) were developed through multi-institutional collaborations, and 32.6% incorporated transdisciplinary participation. Only 31.8% reported technical metrics (commonly area under the curve, sensitivity, and specificity), and 15.9% provided clinical indicators (eg, predictive values or calibration). Only 11.4% achieved methodological rigor, incorporating validation aligned with intended use, while most relied on partial validation strategies, highlighting a gap between model development and clinical evaluation. Ethical integration was heterogeneous: only 5 frameworks achieved high compliance (≥80%), whereas 4 scored <10%. The most frequently addressed UNESCO principles were awareness and education (71.1%) and transparency and explainability (70%), while human oversight (24.4%) and adaptive governance (33.3%) were least represented. Findings indicate a misalignment between framework design, validation requirements, and clinical implementation.

**Conclusions:**

Evaluation frameworks for clinical AI remain heterogeneous and oriented toward investigational contexts. Critical gaps persist in methodological rigor, validation aligned with intended use, and fragmented ethical coverage. These findings highlight the need for standardized, robust, and ethically grounded frameworks to enable safe, reliable, and scalable integration of AI into clinical practice.

## Introduction

AI has evolved substantially since its conceptualization in the mid-20th century, when early research explored the possibility of machines emulating human cognitive functions. Driven by advances in computational power, algorithm development, and access to large-scale datasets, AI has expanded from theoretical domains to widespread applications, including health care. Current uses include medical image analysis, disease risk prediction, drug discovery, and treatment personalization [1-3].

In recent years, the number of AI applications proposed for health care has increased rapidly, with hundreds of models developed for diagnostic support, risk prediction, and workflow optimization. However, only a limited proportion of these models have undergone rigorous external validation or prospective clinical evaluation, raising concerns about their readiness for real-world clinical implementation [4-6].

Despite these advances, several challenges continue to limit the integration of AI into clinical practice [7]. These include variability in data quality, limited external validation, lack of transparency in algorithm development, and insufficient assessment of clinical impact in real-world settings [8]. Ensuring accuracy, safety, and reproducibility, while addressing ethical and regulatory requirements, remains essential. The absence of standardized validation procedures and reporting practices represents a major barrier to reliable clinical adoption.

In response, multiple guidelines and evaluation frameworks have been developed to improve the quality, transparency, and clinical applicability of AI models [9]. This lack of standardized evaluation approaches has prompted increasing concern among clinicians, regulators, and researchers regarding the reproducibility, safety, and transparency of AI-based clinical tools [10].

Notable initiatives include the TRIPOD+AI (Transparent Reporting of Multivariable Prediction Models with Artificial Intelligence) [11], the CONSORT-AI (Consolidated Standards of Reporting Trials for Artificial Intelligence) [12], and the CLAIM (Checklist for Artificial Intelligence in Medical Imaging) [13]. More recently, additional initiatives such as STARD-AI (Standards for Reporting Diagnostic Accuracy) [14] and PRISMA-AI (Preferred Reporting Items for Systematic Reviews and Meta-Analyses extension for Artificial Intelligence) [15] have further expanded reporting standards for diagnostic accuracy studies and systematic reviews involving AI technologies. These frameworks provide structured approaches for assessing data quality, methodological rigor, performance metrics, and clinical utility [16].

However, substantial heterogeneity persists across these initiatives, particularly in methodological scope, validation strategies, and the integration of ethical considerations. This variability makes it difficult for researchers and health care systems to identify appropriate approaches for evaluating AI systems intended for clinical use [17,18].

The objective of this scoping review was to systematically map, characterize, and critically analyze existing evaluation and reporting frameworks for clinical AI. Specifically, this review aimed to examine how these frameworks conceptualize and operationalize: (1) methodological rigor and validation strategies, including internal and external validation; (2) claims related to clinical applicability and real-world use; and (3) the integration of ethical principles, with particular emphasis on alignment with the 10 ethical domains proposed by the United Nations Educational, Scientific and Cultural Organization (UNESCO).

## Methods

### Protocol and Registration

We conducted a scoping review incorporating a quantitative documentary analysis to examine evaluation frameworks for AI models in clinical practice. The methodology was guided by the PRISMA-ScR (Preferred Reporting Items for Systematic Reviews and Meta-Analyses extension for Scoping Reviews) [19], the PRISMA (Preferred Reporting Items for Systematic Reviews and Meta-Analyses) 2020 for Abstracts Checklist [20], and was registered in PROSPERO (CRD420251019640).

The process involved the following two main stages: (1) a systematic search and selection of information sources and (2) data extraction and comparative analysis across predefined thematic domains. These domains encompassed (1) the general characteristics of each AI evaluation framework, (2) validation aspects (eg, performance metrics and generalizability), and (3) the integration of ethical principles.

### Eligibility Criteria

All searches covered literature up to February 28, 2026, with no start date limit and no language or publication status restrictions applied.

We included documents that proposed or described an evaluation or reporting framework for clinical AI, encompassing the following source types:

- Peer-reviewed scientific papers: publications in scientific journals presenting frameworks or guidelines for AI evaluation.
- Gray literature documents: documents such as white papers, technical reports, or policy guidelines not formally published in peer-reviewed journals.
- Official websites of relevant organizations: frameworks or directives available on official websites of relevant health, research, or regulatory organizations.
- Introductory series or conceptual papers without real-world application or empirical evaluation.
- Comparative or descriptive analyses of existing AI checklists.
- Documents from out-of-scope domains, including expert conference proceedings
- Letters to the editor, commentaries, or editorial responses lacking original data
- Other reasons

Publications were excluded if they did not present a tangible AI evaluation framework or were outside the scope of our review. Specifically, we excluded methodological or development protocols without validation results or empirical testing of AI models; introductory series or conceptual papers without real-world application or empirical evaluation; comparative or descriptive analyses of existing AI checklists; documents from out-of-scope domains, including expert conference proceedings; letters to the editor, commentaries, or editorial responses lacking original data; and other reasons. Duplicate frameworks reported in multiple sources were counted only once.

### Information Sources

Comprehensive searches were performed in six electronic databases: PubMed (MEDLINE), BVS, Embase, EBSCOhost (including CINAHL and others), Sage Journals, and ProQuest Central. The process was conducted independently by DCLM and MH-P.

In addition, we searched pertinent gray literature sources. The EQUATOR (Enhancing the Quality and Transparency of Health Research) Network was used to identify relevant reporting guidelines for AI, and we screened the official websites of major organizations and consortia involved in clinical AI for any published frameworks [21]. This search was conducted by NMA and DCLM.

### Search

The search strategy combined controlled vocabulary terms (eg, MeSH and Emtree headings) with free-text keywords, ensuring comprehensive coverage of the following three concept areas: (1) AI and machine learning in health care, (2) model validation and performance evaluation, and (3) ethical principles and frameworks. An example PubMed search strategy is provided below:

(“Artificial Intelligence”[Mesh] OR “Artificial Intelligence”[tiab] OR “Machine Learning”[Mesh] OR “Machine Learning”[tiab] OR “Deep Learning”[tiab] OR “Neural Networks, Computer”[Mesh] OR “Neural Network*”[tiab] OR “Natural Language Processing”[Mesh] OR “Natural Language Processing”[tiab] OR “Generative AI”[tiab] OR “Explainable AI”[tiab]) AND (“Validation Studies as Topic”[Mesh] OR “Validation”[tiab] OR “External Validation”[tiab] OR “Model Validation”[tiab] OR “Generalizability”[tiab] OR “Calibration”[tiab] OR “Performance Metrics”[tiab] OR “Reproducibility of Results”[Mesh] OR “Overfitting”[tiab]) AND (“Decision Support Systems, Clinical”[Mesh] OR “Clinical Decision Support”[tiab] OR “Clinical Decision-Making”[Mesh] OR “Predictive Models”[Mesh] OR “Prediction Model*”[tiab] OR “Prognostic Model*”[tiab] OR “Risk Prediction”[tiab] OR “Diagnostic Accuracy”[tiab] OR “Computer-Assisted Diagnosis”[Mesh]) AND (“Ethics, Medical”[Mesh] OR “Bioethics”[Mesh] OR “Ethic*”[tiab] OR “Equity”[tiab] OR “Transparency”[tiab] OR “Accountability”[tiab] OR “Informed Consent”[tiab] OR “Patient Autonomy”[tiab] OR “Privacy”[tiab] OR “Confidentiality”[tiab] OR “Responsible AI”[tiab] OR “Evaluation Framework*”[tiab] OR “Reporting Guideline*”[tiab] OR “CONSORT-AI”[tiab] OR “SPIRIT-AI”[tiab] OR “TRIPOD-AI”[tiab] OR “STARD-AI”[tiab] OR “CLAIM”[tiab]).

Equivalent search queries were adapted for each database using the respective controlled vocabulary and syntax (refer to Table S1 in Multimedia Appendix 1 for the full search strategies).

### Selection of Sources of Evidence

The search strategies were reviewed by the study coauthors. All references retrieved were exported into Microsoft Excel, which was used for reference management, deduplication, and subsequent screening steps. First, 2 reviewers (ACHA and NMA) independently screened the titles and abstracts of all unique records against the eligibility criteria. Next, full-text papers or documents were obtained and assessed for inclusion by the same 2 reviewers working in duplicate. Any discrepancies or uncertainties in study selection were resolved through discussion; if consensus could not be reached, a third reviewer served as an adjudicator.

### Data Charting Process

A standardized data charting form was used to extract relevant information from each included source, structured according to the 3 key domains of interest. For each included framework, data were charted and organized into comparative tables corresponding to general framework characteristics, validation and performance evaluation aspects, and ethical considerations.

Two reviewers collaboratively performed the data extraction, with cross-verification by the broader team to ensure consistency. Any discrepancies were resolved through discussion, and when consensus could not be reached, a third reviewer adjudicated (DCLM).

### General Characteristics

General characteristics extracted and compared across frameworks included development team composition, disciplinary scope, methodological approach, structural domains, extensions, and stated purpose. Development team composition was categorized as unitary, binary, ternary, or quaternary, including the involvement of one, two, three, or four or more institutions, respectively. Disciplinary scope was defined as interdisciplinary if the team included members from different academic fields, and transdisciplinary if it also incorporated nonacademic stakeholders. Structural domains were grouped into IMRD (Introduction, Methods, Results, and Discussion) and non-IMRD formats. Methodological approach, stated purpose, and extensions were recorded exactly as reported in each evaluation framework.

### Validation and Performance Aspects

We extracted details to understand each framework’s orientation toward clinical use and its recommended validation stringency. Each framework’s intended purpose or primary use case was noted and categorized as either research-focused (primarily for studies developing or reporting AI models) or clinical utility-focused (intended to guide evaluation for real-world implementation). For clinically oriented frameworks, we further noted the specified application domain (eg, diagnostic vs prognostic models) and any criteria used to define “clinical utility” [9].

To appraise methodological rigor, we collected information on the following three key validation parameters defined a priori: technical performance, clinical performance, and generalizability [22]. Technical performance (internal validity) measured using metrics such as sensitivity, specificity, area under the curve (AUC) or receiver operating characteristic (ROC), and free-response ROC curve (FROC). ROC and FROC were classified as internal validation parameters, as they are primarily influenced by threshold effects rather than disease prevalence [23,24]. Clinical performance (external validity), assessed using predictive values (positive and negative) and calibration accuracy in AI models. These metrics are influenced by disease prevalence and therefore better reflect real-world performance [22]. Generalizability, defined as the framework’s ability to account for limitations related to uncontrolled overfitting. Overfitting occurs when an algorithm is trained and tested on the same dataset used for development or when internal data splits are used instead of independent or external datasets for validation [25].

- Technical performance (internal validity): measured using metrics such as sensitivity, specificity, AUC or ROC, and FROC. ROC and FROC were classified as internal validation parameters, as they are primarily influenced by threshold effects rather than disease prevalence [23,24].

For this review, evaluation frameworks were classified a priori into three levels of methodological rigor with respect to their assessment of clinical utility and generalizability:

- Complete evaluation: defined as frameworks that require validation using independent external datasets distinct from those used for model training, while also considering the epidemiological study design (eg, diagnostic cohort studies or case-control designs). This approach ensures both internal validity (the model performs adequately in its development sample) and external validity (the model retains performance across real-world clinical settings). Frameworks in this category provide the strongest evidence of clinical reliability, as they minimize overfitting and explicitly account for study design quality.
- Partial evaluation: defined as frameworks that acknowledge the importance of external validation datasets but do not assess the methodological quality or epidemiological design of those datasets. Although this category represents a step toward generalizability, it does not guarantee that external data are sufficiently robust or representative. Consequently, such frameworks provide only limited evidence to support clinical implementation, as risks of selection bias and prevalence-related distortions remain.
- Inadequate evaluation: defined as frameworks that consider AI models to be generalizable even when validation is based solely on internal datasets, typically through data splitting or cross-validation within the same population. This approach fails to ensure external validity and is highly susceptible to overfitting, as reported performance primarily reflects adaptation to the original dataset. Frameworks in this category should not be regarded as sufficient to support clinical applicability; rather, they are relevant only for the developmental phase of model assessment.

### Synthesis of Results

We synthesized the charted data through a descriptive comparative approach, emphasizing both the distribution of characteristics across frameworks and the identification of patterns or gaps in current practice. Extracted information was tabulated and compared across the three thematic domains to facilitate a side-by-side analysis of frameworks (eg, allowing readers to contrast different frameworks’ requirements for validation or ethical compliance).

A dot plot was used as a graph to present the gap map, following the approach suggested by Nyanchoka et al [26]. The evaluation of gaps was performed across 3 predefined domains. For each domain, frameworks were assessed according to specific criteria, and scores were assigned to indicate the presence or absence of gaps.

In Domain 1 “general characteristics,” frameworks were classified as having no gap (1 point) when they incorporated quaternary teams and a transdisciplinary scope. Frameworks that only included an interdisciplinary scope or other team compositions were considered to have a gap (0 points).

In Domain 2 “validation and performance aspects,” frameworks received a score of no gap (1 point) when they addressed clinical utility and provided comprehensive validation (complete generalizability) and appropriate performance considerations. Frameworks that did not explicitly include these elements were classified as having a gap (0 points).

In Domain 3 “ethics,” frameworks were considered to have no gap (1 point) when they explicitly incorporated direct and indirect principles and did not present missing ethical components (direct + incorrect and no missing components). Frameworks lacking these elements were categorized as having a gap (0 points).

This scoring approach allowed the identification of gaps across domains and facilitated the graphical representation of the distribution of strengths and weaknesses among the evaluated frameworks.

We did not perform a formal quantitative meta-analysis given the qualitative nature of the data; however, we carried out simple quantitative aggregations (counts and percentages) for certain features to summarize prevailing trends (reported in the Results section).

### Protocol Deviations

Deviations from the initial protocol included updating the literature search at a later date than originally planned to ensure the currency of the evidence base, adding a gap map to enhance the presentation of results, incorporating PRISMA-S (Preferred Reporting Items for Systematic Reviews and Meta-Analyses literature search extension) reporting elements to improve transparency of the search process, refining the approach to results synthesis, and expanding the number of databases searched. These modifications were made to strengthen the completeness, transparency, and interpretability of the review and did not change its primary objective.

### Ethical Considerations

The study was approved by the Research Ethics Committee of the Universidad Cooperativa de Colombia (protocol INV3688). The ethical evaluation of the included frameworks followed the UNESCO ethical principles for AI [27]. An ethical scoring matrix (Table S2 in Multimedia Appendix 1 [13-15,17,28-69]) was used to assess each framework across the 10 UNESCO domains, with predefined criteria indicating whether each principle was explicitly addressed (fully covered with clear guidance), partially aligned (mentioned or implied but not fully detailed), or absent. Each principle (eg, transparency, fairness, privacy, human oversight, and accountability) was evaluated individually using a semiquantitative scoring system. Domain-level scores were then calculated to compare the ethical coverage across frameworks. Two reviewers conducted the assessment independently, resolving discrepancies through consensus. This structured matrix-based approach ensured a systematic and reproducible analysis of the ethical strengths and gaps within current AI evaluation frameworks used in clinical practice.

## Results

### Study Selection

**Figure 1.**
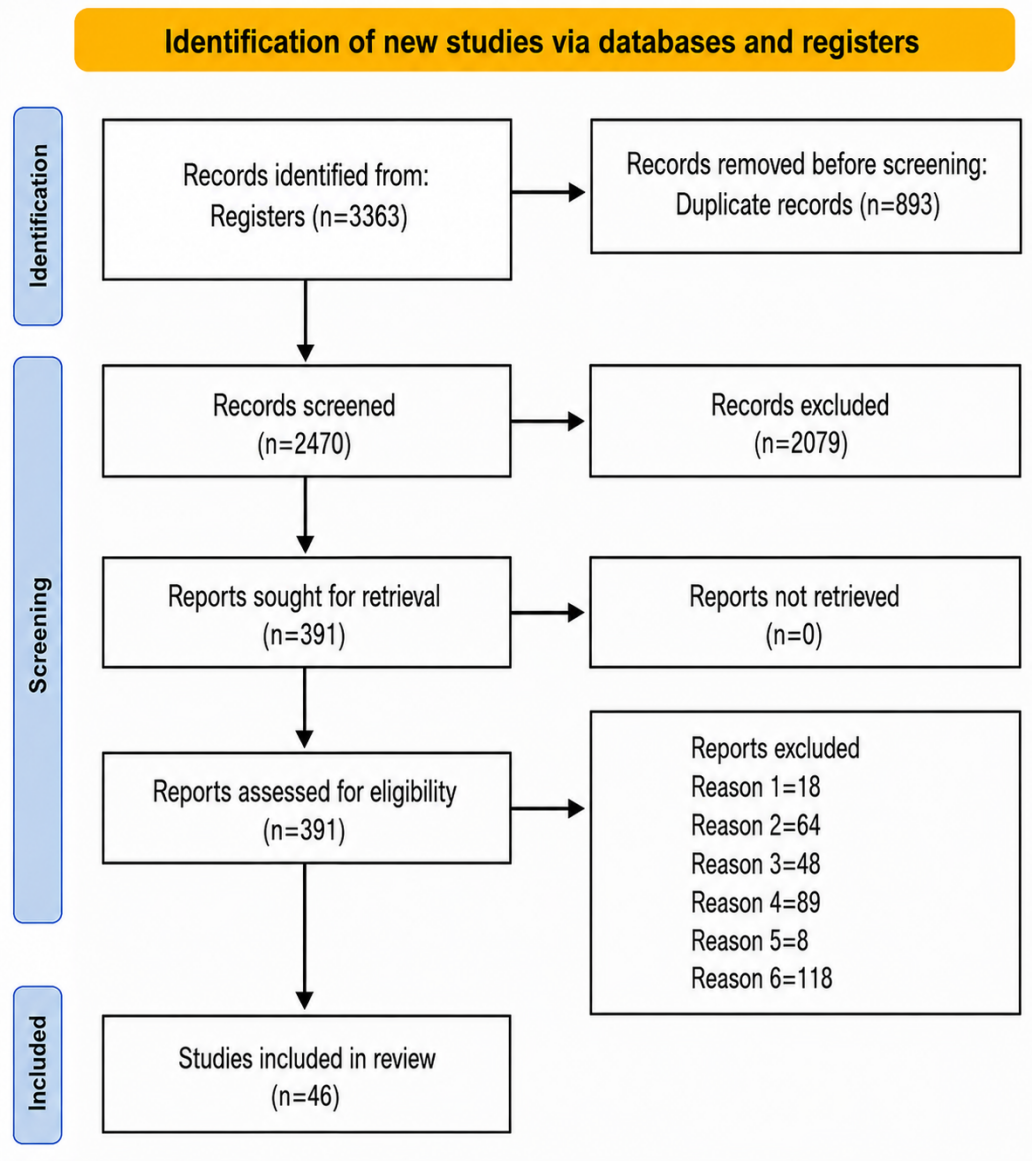
PRISMA (Preferred Reporting Items for Systematic Reviews and Meta-Analyses) flow diagram. Adapted from Page et al [70].

A total of 3363 records were identified through database and gray literature searches. After removal of duplicates, 391 records were screened at the full-text level. Of these, 345 were excluded for not meeting the eligibility criteria, primarily because they did not propose or describe an evaluation framework for clinical AI or lacked evaluative content. Ultimately, 46 distinct evaluation frameworks were included in the scoping review. Figure 1 presents the PRISMA flow diagram detailing the number of records identified, screened, excluded, and finally included in the review. Table 1 summarizes all included papers and their main characteristics, while the complete list of excluded studies with corresponding reasons is available in Multimedia Appendix 2.

**Table 1.** Summary of the included documents: collaboration type, disciplinary scope, development methodology, structural domains, and stated purpose of the identified AI evaluation frameworks.

Author, year, and reference	Title	Development team	Disciplinary scope	Method	General domains	Extension	Purpose
Luo et al, 2016 [28]	Guidelines for Developing and Reporting Machine Learning Predictive Models in Biomedical Research	Quaternary	Interdisciplinary	Delphi method (iterative consensus process via email with 11 experts from 3 institutions across 3 continents).	IMRD^a^	Not applicable	To establish minimum reporting items and procedural steps ensuring valid application, transparent reporting, and reproducibility of machine learning predictive models in biomedical research.
Floridi et al, 2018 [29]	AI4People—An Ethical Framework for a Good AI Society: Opportunities, Risks, Principles, and Recommendations	Quaternary	Transdisciplinary	Consensus-based ethical synthesis and policy Delphi integrating comparative analysis of existing AI ethics principles and collaborative drafting of 20 action-oriented recommendations.	Non-IMRD	Not applicable	To formulate a comprehensive ethical framework and actionable policy roadmap for building a “Good AI Society” that aligns technological innovation with human dignity, social justice, and accountability.
Reps et al, 2018 [30]	Standardized Framework for Generating and Evaluating Patient-Level Prediction Models Using Observational Healthcare Data	Ternary	Interdisciplinary	Framework design and proof-of-concept implementation integrating best-practice guidelines with the OMOP^b^ Common Data Model; implemented via open-source R packages (R Core Team), (Patient Level Prediction) within the OHDSI^c^ network.	IMRD	Not applicable	To provide a standardized, transparent, and reproducible framework for developing, validating, and sharing patient-level predictive models across observational health care databases using a common data model.
Cruz Rivera et al, 2020 [31]	Guidelines for clinical trial protocols for interventions involving artificial intelligence: the SPIRIT-AI^d^ Extension	Quaternary	Transdisciplinary	Multistage consensus-based development: literature review, expert consultation, generation of 26 candidate items, 2-round Delphi (103 participants), international consensus meeting (31 stakeholders), checklist pilot (34 participants), and final EQUATOR^e^-registered extension.	IMRD	EQUATOR—Official reporting guideline extension of SPIRIT 2013 (SPIRIT-AI)	To establish AI-specific protocol reporting standards ensuring transparency, reproducibility, safety, and completeness in clinical trial protocols evaluating AI interventions.
Hernandez-Boussard et al, 2020 [32]	MINIMAR (MINimum Information for Medical AI Reporting): Developing reporting standards for artificial intelligence in health care	Unitary	Interdisciplinary	Conceptual proposal based on expert opinion and synthesis of existing reporting standards (MIAME^f^, CONSORT^g^, SPIRIT^h^, PRISMA^i^, STROBE^j^, and TRIPOD^k^); no Delphi or consensus process.	Non-IMRD	Not applicable	To propose minimum reporting standards for AI and ML^l^ models in health care, focusing on transparency of training data, model design, population characteristics, and validation to improve reproducibility and mitigate bias.
Liu et al, 2020 [33]	Reporting guidelines for clinical trial reports for interventions involving artificial intelligence: the CONSORT-AI^m^ Extension	Quaternary	Transdisciplinary	Staged consensus process: literature review, candidate item generation, 2-round Delphi survey (103 participants), international consensus meeting (31 participants), pilot testing (34 participants), and development using EQUATOR^e^ reporting-guideline methodology.	IMRD	CONSORT-AI (EQUATOR)	To develop a reporting guideline extension (CONSORT-AI) specifying additional items required for transparent, complete, and reproducible reporting of randomized clinical trials evaluating AI-based interventions.
Mongan et al, 2020 [34]	Checklist for Artificial Intelligence in Medical Imaging (CLAIM)	Binary	Interdisciplinary	Consensus-based guideline development integrating and adapting existing reporting standards (STARD^n^, STROBE, CONSORT, and EQUATOR) to AI in medical imaging.	IMRD	Not applicable	To establish a standardized reporting framework for authors and reviewers to ensure transparency, reproducibility, and methodological rigor in studies applying AI to medical imaging.
Norgeot et al, 2020 [35]	Minimum Information about Clinical Artificial Intelligence Modeling (MI-CLAIM) Checklist	Quaternary	Interdisciplinary	Consensus-driven documentation framework: design of a 6-part checklist defining minimal reporting standards for clinical AI modeling; developed collaboratively and released with a public GitHub repository for community feedback.	Non-IMRD	Not applicable	To establish a minimum set of information required for transparent, fair, and reproducible reporting of clinical AI modeling studies.
Sengupta et al, 2020 [36]	Proposed Requirements for Cardiovascular Imaging–Related Machine Learning Evaluation (PRIME) Checklist	Quaternary	Interdisciplinary	Consensus-based methodological synthesis: iterative development of a 7-domain framework through expert review and synthesis of best practices in cardiovascular ML^l^ model design, validation, and reporting.	Non-IMRD	Not applicable	To establish a standardized set of requirements and reporting guidelines for developing, validating, and communicating machine learning models in cardiovascular imaging, ensuring reproducibility, transparency, and methodological consistency.
Stevens et al, 2020 [37]	Recommendations for Reporting Machine Learning Analyses in Clinical Research	Ternary	Interdisciplinary	Consensus-based methodological synthesis: development of structured recommendations integrating existing reporting standards (STROBE and TRIPOD) with machine learning–specific considerations for clinical research.	Non-IMRD	Not applicable	To propose structured recommendations and reporting elements for transparent, reproducible, and interpretable reporting of machine learning analyses in clinical research.
Young et al, 2020 [38]	Artificial Intelligence in Dermatology: A Primer	Binary	Interdisciplinary	Narrative review and conceptual synthesis: integrative appraisal of AI applications in dermatology, analyzing performance, equity, generalizability, and interpretability across existing studies.	Non-IMRD	Not applicable	To provide a comprehensive overview and conceptual framework for understanding the capabilities, limitations, and ethical challenges of AI in dermatology, with proposed metrics for reporting model performance.
Cabitza and Campagner, 2021 [39]	The need to separate the wheat from the chaff in medical informatics: Introducing a comprehensive checklist for the (self)-assessment of medical AI studies	Unitary	Interdisciplinary	Checklist development based on CRISP-DM^o^ methodology: synthesis of prior frameworks (MINIMAR^p^, CONSORT-AI, SPIRIT-AI, MI-CLAIM^q^, WHO^r^ or ITU^s^ ML4H^t^, PROBAST, and TRIPOD) into a 30-item tool organized in six phases: problem understanding, data understanding, data preparation, modeling, validation, and deployment.	Non-IMRD	Not applicable	To introduce a comprehensive 30-item checklist to assist authors and reviewers in evaluating the methodological soundness, reproducibility, and quality of medical AI studies, aligning with international reporting standards.
Ji et al, 2021 [40]	Evaluation Framework for Successful Artificial Intelligence–Enabled Clinical Decision Support Systems: Mixed Methods Study	Ternary	Interdisciplinary	Mixed methods design combining Delphi process, cognitive interviews, and structural equation modeling development and psychometric validation of a 28-item measurement instrument assessing AI-CDSS^u^ success across six latent variables (system, information, and service quality; perceived ease of use; perceived benefit; user acceptance).	IMRD	Not applicable	To develop and validate a comprehensive evaluation framework identifying key determinants of success for AI-enabled clinical decision support systems, focusing on user acceptance as the central construct.
Olczak et al, 2021 [41]	Presenting Artificial Intelligence, Deep Learning, and Machine Learning Studies to Clinicians and Healthcare Stakeholders: An Introductory Reference with a Guideline and a Clinical AI Research (CAIR) Checklist Proposal	Quaternary	Interdisciplinary	Consensus-driven guideline development: synthesis of key methodological, statistical, and ethical principles in medical AI, culminating in the CAIR^v^ checklist and reporting recommendations for clinicians and AI researchers.	IMRD	Not applicable	To propose a comprehensive reporting and evaluation checklist (CAIR) for presenting and interpreting medical AI studies, enhancing communication between engineers, clinicians, and health care stakeholders.
Schwendicke et al, 2021 [42]	Artificial Intelligence in Dental Research: Checklist for Authors, Reviewers, and Readers	Quaternary	Transdisciplinary	Consensus-based e-Delphi process following CREDES^w^ guidelines, integrating prior frameworks (CLAIM^x^, STARD, TRIPOD, CONSORT-AI, SPIRIT-AI, RECORD)^y^; resulted in a 31-item checklist for authors, reviewers, and readers.	IMRD	Not applicable	To provide a 31-item consensus checklist for improving the planning, conduct, and reporting of AI studies in dental research, enhancing reproducibility, transparency, and methodological rigor.
Bazoukis et al, 2022 [43]	The inclusion of augmented intelligence in medicine: A framework for successful implementation	Quaternary	Transdisciplinary	Conceptual synthesis and policy framework development integrating ethical, regulatory, and practical dimensions of AI adoption into a unified framework addressing reliability, oversight, liability, equity, patient rights, and cybersecurity.	Non-IMRD	Not applicable	To propose an integrated regulatory and operational framework to guide developers, clinicians, researchers, and regulators in the safe and equitable implementation of augmented intelligence in clinical practice.
Daneshjou et al, 2022 [44]	CheckList for Evaluation of Image-Based AI Reports in Dermatology (CLEAR Derm): Consensus Guidelines from the International Skin Imaging Collaboration Artificial Intelligence Working Group	Quaternary	Transdisciplinary	Two-round virtual consensus process: systematic literature review (PubMed 2008‐2021) followed by expert panel consensus (19 ISIC^z^ members) using Delphi methodology to develop a 25-item checklist grouped into four domains (data, technique, technical assessment, and application).	IMRD	Yes – aligns with EQUATOR or PRISMA-related initiatives (referencing STARD-AI, CONSORT-AI, SPIRIT-AI, and DECIDE-AI^aa^)	To establish a consensus-based, dermatology-specific checklist (CLEAR Derm) for evaluating image-based AI studies, ensuring fairness, reliability, transparency, and ethical clinical translation.
Fusar-Poli et al, 2022 [45]	Development and validation of the Clinical Artificial Intelligence Research (CAIR) checklist for reporting clinical AI studies: a multi-phase international consensus study	Quaternary	Transdisciplinary	Multiphase international Delphi consensus: four-stage process (literature review, Delphi rounds with >150 experts from 26 countries, pilot testing, and validation) to develop a 22-item checklist for transparent and standardized reporting of clinical AI research.	IMRD	Yes – aligned with EQUATOR or PRISMA-related initiatives; complementary to CONSORT-AI, SPIRIT-AI, and TRIPOD-AI^ab^	To create a validated, consensus-based reporting guideline (CAIR) ensuring transparency, reproducibility, and clinical relevance in studies applying AI to health care research and practice.
Kwong et al, 2021 [46]	Standardized Reporting of Machine Learning Applications in Urology: The STREAM-URO Framework	Quaternary	Interdisciplinary	PRISMA-guided systematic literature review and expert item synthesis resulting in a 26-item checklist mapped a TRIPOD.	IMRD	PRISMA followed; no specific PRISMA extension reported	To develop a urology-specific, standardized reporting framework for ML studies to improve transparency, reproducibility, comparability, and clinical uptake.
Lu et al, 2022 [47]	Assessment of Adherence to Reporting Guidelines by Commonly Used Clinical Prediction Models From a Single Vendor: A Systematic Review	Unitary	Interdisciplinary	Systematic review (PRISMA-based): MEDLINE search (Nov 2020 to Dec 2020) identifying 15 model reporting guidelines; synthesis of 220 unique items and cross-sectional evaluation of 12 deployed AI models from Epic Systems using expert adjudication.	IMRD	PRISMA followed; no specific PRISMA extension reported	To evaluate the overlap among existing AI model reporting guidelines and assess how well the documentation of widely deployed models adheres to these standards, identifying reporting gaps in reliability and fairness.
Shen et al, 2022 [48]	An Ethics Checklist for Digital Health Research in Psychiatry: Viewpoint	Ternary	Transdisciplinary	Stakeholder-informed consensus workshop: interdisciplinary and stakeholder meeting (May 2020) supported by NIH^ac^ Bioethics Supplement; qualitative synthesis leading to a 20-item ethics checklist across six domains (informed consent, equity and access, privacy and partnerships, regulation and law, return of results, and duty to warn or report).	Non-IMRD	Not applicable	To develop a 20-question ethics checklist addressing procedural safeguards and ethical, legal, and social implications (ELSI) in digital psychiatry and deep phenotyping research.
Vasey et al, 2022 [49]	Reporting guideline for the early-stage clinical evaluation of decision support systems driven by artificial intelligence: DECIDE-AI	Quaternary	Transdisciplinary	Two-round modified Delphi + virtual consensus meeting conducted according to EQUATOR Network standards; 151 experts from 18 countries and 20 stakeholder groups participated; guideline registered on EQUATOR and Open Science Framework.	IMRD	Yes – official EQUATOR or PRISMA-related extension; complements CONSORT-AI, SPIRIT-AI, TRIPOD-AI, and STARD-AI	To provide a multistakeholder, consensus-based reporting guideline (DECIDE-AI) for the early clinical evaluation of AI-based decision support systems, focusing on clinical utility, safety, human factors, and readiness for large-scale trials.
Abdulazeem et al, 2023 [50]	A systematic review of clinical health conditions predicted by machine learning diagnostic and prognostic models trained or validated using real-world primary health care data	Binary	Interdisciplinary	Systematic review (PRISMA-based): registered on PROSPERO (CRD42021264582); databases: Cochrane, PubMed, Web of Science, Elsevier, BioRxiv, ACM, IEEE; screening with Rayyan (Rayyan Systems Inc., Cambridge, MA, USA); risk of bias via PROBAST and methodological appraisal via CHARMS^ad^.	IMRD	PRISMA (2020) followed; no extension reported	To identify clinical conditions targeted by ML models trained or validated using real-world primary health care (PHC) data, and to map the methodological characteristics, validation approaches, and performance measures of these models.
Cacciamani et al, 2023 [15]	PRISMA-AI: Reporting guidelines for systematic reviews and meta-analyses on AI in healthcare	Quaternary	Transdisciplinary	Multiphase development process: literature review, Delphi survey among multidisciplinary experts, consensus meeting, piloting, and creation of the PRISMA-AI^ae^ checklist and explanation or elaboration document; guideline registered with EQUATOR and ClinicalTrials.gov (NCT05382455).	Non-IMRD	Yes–official PRISMA (PRISMA-AI)	To establish PRISMA-AI, a reporting guideline extension for systematic reviews and meta-analyses addressing AI-based interventions in health care, ensuring transparency, reproducibility, and clinical applicability.
Debray et al, 2023 [51]	Transparent reporting of multivariable prediction models developed or validated using clustered data (TRIPOD-Cluster^af^): explanation and elaboration	Quaternary	Interdisciplinary	Delphi consensus and methodological synthesis: iterative expert consensus based on prior TRIPOD, TRIPOD-E&E^ag^ (2015), and empirical evaluations of clustered datasets (individual participant data meta-analyses and EHR^ah^-based studies); resulted in a 19-item checklist.	IMRD	Yes–TRIPOD-Cluster builds upon the original TRIPOD and aligns with PRISMA-IPD, EQUATOR, and PROBAST frameworks	To provide guidance for transparent reporting of multivariable prediction models developed or validated using clustered data, addressing heterogeneity, generalizability, and bias in individual participant data meta-analyses and multicenter or EHR studies.
Elvidge et al, 2023 [52]	Consolidated Health Economic Evaluation Reporting Standards for Interventions that use Artificial Intelligence (CHEERS-AI)	Quaternary	Transdisciplinary	Multiphase development: (1) initiation (steering group + longlist), (2) three-round Delphi study with 58‐31 experts, (3) consensus meeting, (4) patient involvement via EURORDIS Digital Advisory Group, (5) piloting of items on 9 economic evaluations, and (6) final steering group ratification.	IMRD	Not PRISMA – this is an official CHEERS extension (CHEERS-AI), aligned with EQUATOR, not PRISMA.	To develop a reporting guideline extension ensuring transparent, complete, and reproducible reporting of economic evaluations of AI-based health interventions, adding AI-specific items to CHEERS-2022.
Klement and El Emam, 2023 [53]	Consolidated Reporting Guidelines for Prognostic and Diagnostic Machine Learning Modeling Studies: Development and Validation	Binary	Interdisciplinary	Scoping-review–informed consolidation of reporting guidelines: broad literature search (192 papers), screening against predefined criteria, quality appraisal using a 9-item checklist, extraction of reporting items from 17 high-quality guidelines; followed by external expert review (JMIR AI editorial board) and initial validation on 6 ML studies.	IMRD	PRISMA followed (for the ML guideline search); no specific PRISMA extension reported	To produce a single consolidated checklist of reporting items for prognostic and diagnostic ML modeling studies (in-silico and shadow-mode), improving transparency, reproducibility, and methodological clarity.
Kwong et al, 2023 [54]	APPRAISE-AI^ai^ Tool for Quantitative Evaluation of AI Studies for Clinical Decision Support	Quaternary	Interdisciplinary	Framework development informed by literature review + expert panel refinement; includes reliability testing (interrater and intrarater ICCs^aj^), construct validation (correlation with expert scores, citation rates, QUADAS-2^ak^, and TRIPOD), and application to a published systematic review of sepsis prediction models.	IMRD	Not applicable	To develop and validate APPRAISE-AI, a quantitative scoring tool that assesses the methodological rigor, reporting quality, and robustness of clinical AI studies across six domains (clinical relevance, data quality, methodological conduct, robustness, reporting quality, reproducibility).
Murphy et al, 2023 [55]	A guide to optometrists for appraising and using artificial intelligence in clinical practice	Binary	Interdisciplinary	Narrative review with expert-informed synthesis: conceptual explanation of AI fundamentals for optometry, followed by a practical checklist addressing regulatory approval, intended use, clinical applicability, population fit, performance metrics, and explainability.	Non-IMRD	Not applicable	To provide optometrists with a practical, clinically oriented checklist for appraising whether AI systems are safe, appropriate, and suitable for use in routine optometric practice.
Collins et al, 2024 [56]	TRIPOD + AI statement: updated guidance for reporting clinical prediction models that use regression or machine learning methods	Quaternary	Transdisciplinary	Multiphase consensus process following EQUATOR guidance: literature review, item generation, modified Delphi (2 rounds, 170‐200 participants); patient or public involvement meeting; online consensus meeting (28 participants); checklist refinement and expansion.	IMRD	TRIPOD family (EQUATOR): TRIPOD-Cluster, TRIPOD-SRMA, TRIPOD-LLM^al^, TRIPOD-AI (TRIPOD + AI). Not a PRISMA extension.	To provide an updated, harmonized reporting guideline (TRIPOD + AI) for transparent, complete, and accurate reporting of prediction model studies using regression or machine learning methods.
Elfer et al, 2024 [57]	Reproducible Reporting of the Collection and Evaluation of Annotations for Artificial Intelligence Models	Quaternary	Interdisciplinary	Expert-informed operationalization of a prior workflow (Wahab et al[57,71]) into a reporting framework + checklist, demonstrated through application to an annotation project (HTT^am^). Includes iterative refinement, piloting, expert review, and quality assessment of dataset construction processes. No Delphi or formal consensus process.	IMRD	CLEARR-AI (not a PRISMA, TRIPOD, CONSORT, SPIRIT, STARD, DECIDE, or CHEERS extension. Conceptually aligned with EQUATOR AI extensions but not an official extension.)	To develop and demonstrate CLEARR-AI, a reporting framework and checklist for transparent, reproducible documentation of medical image annotation datasets used in AI model development, validation, and evaluation.
El Emam et al, 2024 [58]	Consolidated Reporting Guidelines for Prognostic and Diagnostic Machine Learning Models (CREMLS)	Binary	Interdisciplinary	Editorial synthesis describing CREMLS^an^ checklist (developed previously via structured literature review and quality appraisal), illustrating item application through published examples; not a new framework development study.	Non-IMRD	CREMLS (independent checklist)	To present and formalize JMIR Publications’ adoption of the CREMLS checklist for improving completeness, methodological transparency, and reproducibility in diagnostic and prognostic ML studies.
Guni A et al, 2024 [59]	Revised Tool for the Quality Assessment of Diagnostic Accuracy Studies Using AI (QUADAS-AI): Protocol for a Qualitative	Quaternary	Transdisciplinary	Three-stage development protocol: (1) project organization; (2) item generation (mapping review, meta-research study, international scoping survey, PPIE^ao^ focus group); (3) modified Delphi process (multiple online rounds and consensus meeting); piloting and drafting of QUADAS-AI tool and explanation or elaboration document.	IMRD	EQUATOR: QUADAS family extension (in development). Not PRISMA.	To develop QUADAS-AI, an AI-specific quality assessment tool for systematic reviews evaluating the diagnostic accuracy of AI systems; addressing biases, applicability, and methodological challenges unique to AI-based diagnostic studies.
Kapoor et al, 2024 [60]	REFORMS: Consensus-based Recommendations for Machine-learning-based Science	Quaternary	Interdisciplinary	Consensus-based development: extensive literature review + multiple rounds of internal expert revision + virtual consensus discussions among 19 researchers; no Delphi, no PPIE, no stakeholder outreach.	Non-IMRD	Independent checklist. NOT a PRISMA extension; NOT EQUATOR-registered; NOT: TRIPOD, CONSORT, SPIRIT, STARD, CHEERS	To establish cross-disciplinary recommendations and a 32-item checklist to improve rigor, reproducibility, transparency, and error detection in machine-learning–based science.
Labkoff et al, 2024 [61]	Toward a responsible future: recommendations for AI-enabled clinical decision support	Quaternary	Transdisciplinary	Large-scale consensus process: four introductory webinars (ethics or religion, patient perspectives, regulatory context, and risk management), a 2-d in-person workshop with >200 stakeholders, breakout groups, iterative qualitative synthesis, and 4-month iterative Delphi-like consensus process among authors.	IMRD	Independent consensus framework; not PRISMA or EQUATOR	To produce consensus-based, cross-stakeholder recommendations for trustworthy, safe, effective, and equitable development, validation, implementation, certification, monitoring, and lifecycle governance of AI-enabled clinical decision support (AI-CDS) systems.
Masters and Salcedo, 2024 [62]	A checklist for reporting, reading, and evaluating Artificial Intelligence Technology Enhanced Learning (AITEL) research in medical education	Binary	Interdisciplinary	Narrative, expert-informed development: multisource review of ISO^ap^ standards, FDA^aq^ guidance, EU AI^ar^ Act, educational frameworks; iterative refinement; workshop feedback (AMEE 2022 [62]); internal expert review; no Delphi and no consensus panel.	Non-IMRD	Not applicable	To propose a structured reporting checklist for describing, evaluating, and understanding AI-based Technology-Enhanced Learning (AITEL) systems in medical education, improving transparency, reproducibility, and ethical oversight.
Ning et al, 2024 [17]	Generative artificial intelligence and ethical considerations in health care: a scoping review and ethics checklist	Quaternary	Interdisciplinary	Systematic scoping review (193 papers) using PRISMA-ScR^as^, followed by thematic synthesis of ethical principles, generation of a 10-principle ethics checklist (TREGAI^at^); iterative expert internal review; no Delphi or consensus process; checklist maintained as a live online document.	IMRD	PRISMA-ScR (used for the review); TREGAI itself is not a PRISMA or EQUATOR extension	To provide a systematic ethical assessment framework and propose the TREGAI checklist to reinforce transparency, accountability, equity, and responsible practice in generative AI health care research.
Ray et al, 2024 [63]	Decoding skin cancer classification: perspectives, insights, and advances through researchers’ lens	Binary	Interdisciplinary	Narrative, experience-based framework derivation: introduction of 2 practical checklists based on prior educational literature, teaching experience, and iterative refinement within clinical simulation programs; no Delphi or formal guideline development.	Non-IMRD	Not applicable	To introduce 2 practical checklists for faculty preparation and scenario design in AI-enhanced medical simulation, supporting safe, transparent, and intentional incorporation of AI tools in clinical education.
Tejani et al, 2024 [13]	Checklist for Artificial Intelligence in Medical Imaging (CLAIM): 2024 Update	Quaternary	Interdisciplinary	Formal Delphi consensus process: renewal of EQUATOR registration, recruitment of 73 international experts, 2-round Delphi (n=72), steering committee review, restructuring of items, and creation of CLAIM 2024 checklist.	IMRD	EQUATOR-registered reporting guideline (CLAIM 2024) — NOT PRISMA	To revise and formalize CLAIM into the 2024 EQUATOR-registered guideline, improving transparency, reproducibility, and completeness of AI medical imaging research through a structured, expert-derived 44-item reporting checklist.
Uribe et al, 2024 [64]	Integrating Generative AI in Dental Education: A Scoping Review of Current Practices and recommendations	Binary	Interdisciplinary	Scoping review following JBI^au^ methodology; protocol registered on OSF^av^; multisource search (university websites, search engines, and email outreach).	IMRD	PRISMA-ScR (used for reporting the review)	To identify and summarize existing institutional guidelines on generative AI use in dental education; does not create or validate an evaluation framework.
Warren et al, 2024 [65]	An Introductory Guide to Artificial Intelligence in Interventional Radiology: Part 2: Implementation Considerations and Harms	Ternary	Interdisciplinary	Narrative expert-informed framework development, derived from regulatory documents (WHO^aw^, FDA, and IMDRF)^ax^, prior IR AI frameworks, safety science, and authors’ clinical experience; iterative internal refinement; no Delphi, no consensus meeting, no systematic review.	IMRD	Independent implementation framework. Not a PRISMA extension; not EQUATOR-registered	To provide a risk-based framework and an 11-item checklist to guide safe, structured, and context-aware implementation and evaluation of AI tools in interventional radiology practice.
Kalaycıoğlu et al, 2025 [66]	Evaluating the sample size requirements of tree-based ensemble machine learning techniques for clinical risk prediction	Ternary	Interdisciplinary	Extensive simulation study comparing sample size requirements for ensemble MLTs^ay^ (bagging, RF^az^, and boosting) vs logistic regression; uses real datasets + multiple DGMs^ba^; evaluates performance metrics (MAPE^bb^, C-statistic, calibration, and Brier score).	IMRD	Not applicable	To evaluate whether existing sample size formulas for logistic regression apply to tree-based MLTs and to determine sample size requirements for development and external validation of MLT-based clinical risk prediction models.
Pan American Health Organization (PAHO), 2025 [67]	AI prompt design for public health: Using generative AI responsibly	Unitary	Interdisciplinary	Narrative, evidence-informed guidance manual; structured chapters; practical prompt templates; includes a Prompt Review Checklist but no evaluation framework, no Delphi, no consensus process.	Non-IMRD	Not applicable	To provide guidance for responsible prompt design in generative AI applications for public health, including templates, examples, quality control considerations, and a prompt review checklist.
Sounderajah et al, 2025 [14]	STARD-AI Steering Committee. The STARD-AI reporting guideline for diagnostic accuracy studies using artificial intelligence	Quaternary	Transdisciplinary	Multistakeholder development: systematic review, expert survey (80 experts), PPIE focus group, candidate item generation, modified Delphi (2 rounds, >240 participants), preconsensus prioritization, international consensus meeting, and final Steering Committee refinement.	IMRD	EQUATOR — Official reporting guideline extension of STARD 2015 (STARD-AI)	To provide a minimum essential reporting guideline for diagnostic accuracy studies evaluating AI-based tests, improving transparency, reducing bias, enhancing reproducibility, and enabling clinical, regulatory, and policy decision-making.
Tuygunov et al, 2025 [68]	The Transformative Role of Artificial Intelligence in Dentistry: A Comprehensive Overview Part 2: The Promise and Perils, and the International Dental Federation Communique	Quaternary	Interdisciplinary	Narrative concise review synthesizing recent literature and summarizing the FDI White Paper; no Delphi, no consensus, no systematic review.	Non-IMRD	Not applicable	To provide an updated overview of AI in dentistry, including educational uses, patient communication, integration challenges, ethical considerations, and a summary of the FDI AI Communiqué.
Wang et al, 2025 [69]	A practical guide for nephrologist peer reviewers: evaluating artificial intelligence and machine learning research in nephrology	Quaternary	Interdisciplinary	Narrative expert synthesis integrating established guidelines (TRIPOD-AI, TRIPOD-LLM, STARD, STROBE, and CRISP-DM) into an applied framework for peer reviewers; includes an 8-step evaluation schema but no Delphi or consensus or systematic review.	IMRD	TRIPOD-AI; TRIPOD-LLM (EQUATOR extensions used as foundational tools; no new extension created)	To provide a structured, guideline-based evaluation framework enabling nephrologist peer reviewers to appraise AI or machine learning studies rigorously, focusing on validation, dataset quality, bias, interpretability, and real-world applicability.

a IMRD: Introduction, Methods, Results, and Discussion.

b OMOP: Observational Medical Outcomes Partnership.

c OHDSI: Observational Health Data Sciences and Informatics.

d SPIRIT-AI: Standard Protocol Items: Recommendations for Interventional Trials–Artificial Intelligence.

e EQUATOR: Enhancing the Quality and Transparency of Health Research.

f MIAME: Minimum Information About a Microarray Experiment.

g CONSORT: Consolidated Standards of Reporting Trials.

h SPIRIT: Standard Protocol Items: Recommendations for Interventional Trials.

i PRISMA: Preferred Reporting Items for Systematic Reviews and Meta-Analyses.

j STROBE: Strengthening the Reporting of Observational Studies in Epidemiology.

k TRIPOD: Transparent Reporting of a Multivariable Prediction Model for Individual Prognosis or Diagnosis.

l ML: machine learning.

m CONSORT-AI: Consolidated Standards of Reporting Trials for Artificial Intelligence.

n STARD: Standards for Reporting Diagnostic Accuracy Studies.

o CRISP-DM: Cross-Industry Standard Process for Data Mining.

p MINIMAR: MINimum Information for Medical AI Reporting.

q MI-CLAIM: Minimum Information About Clinical Artificial Intelligence Modeling.

r WHO: World Health Organization.

s ITU: International Telecommunication Union.

t ML4H: Machine Learning for Health

u AI-CDSS: Artificial Intelligence–Clinical Decision Support System.

v CAIR: Clinical AI Research.

w CREDES: Conducting and REporting DElphi Studies.

x CLAIM: Checklist for Artificial Intelligence in Medical Imaging.

y RECORD: Reporting of studies Conducted using Observational Routinely-collected health Data.

z ISIC: International Skin Imaging Collaboration.

aa DECIDE-AI: Developmental and Evaluation Checklist for Decision Support Systems Driven by Artificial Intelligence.

ab TRIPOD-AI: Transparent Reporting of a Multivariable Prediction Model for Individual Prognosis or Diagnosis–Artificial Intelligence

ac NIH: National Institutes of Health.

ad CHARMS: Critical Appraisal and Data Extraction for Systematic Reviews of Prediction Modelling Studies.

ae PRISMA-AI: Preferred Reporting Items for Systematic Reviews and Meta-Analyses extension for Artificial Intelligence.

af TRIPOD-Cluster: Transparent Reporting of Multivariable Prediction Models Developed or Validated Using Clustered Data.

ag TRIPOD-E&E: Transparent Reporting of a Multivariable Prediction Model for Individual Prognosis or Diagnosis – Explanation and Elaboration.

ah EHR: electronic health record.

ai APPRAISE-AI: Assessment of Predictive Performance and Bias in Artificial Intelligence Studies.

aj ICC: intraclass correlation coefficient.

ak QUADAS-2: Quality Assessment of Diagnostic Accuracy Studies 2.

al TRIPOD-LLM: Transparent Reporting of a Multivariable Prediction Model for Individual Prognosis or Diagnosis–Large Language Models.

am HTT: High-Throughput Truthing project

an CREMLS: Consolidated Reporting of Machine Learning Studies.

ao PPIE: Patient and Public Involvement and Engagement.

ap ISO: International Organization for Standardization.

aq FDA: Food and Drug Administration.

ar EU AI: European Union Artificial Intelligence Act.

as PRISMA-ScR: Preferred Reporting Items for Systematic Reviews and Meta-Analyses extension for Scoping Reviews

at TREGAI: Transparent Reporting of Ethics for Generative Artificial Intelligence.

au JBI: Joanna Briggs Institute.

av OSF: Open Science Framework.

aw WHO: World Health Organization.

ax IMDRF: International Medical Device Regulators Forum.

ay MLT: Machine Learning Technique.

az RF: random forest.

ba DGM: data-generating mechanism.

bb MAPE: mean absolute prediction error.

### Domain 1: General Characteristics

The included publications, spanning from 2016 to 2025, illustrate a substantial expansion of methodological and reporting initiatives aimed at strengthening the evaluation of AI systems in health care. A majority of frameworks (63%, 29/46) were developed through quaternary, multi-institutional collaborations, and 32.6% (15/46) involved transdisciplinary participation, including regulators, industry partners, policymakers, or patient representatives.

With respect to methodological development, more than half of the frameworks were created using formal consensus processes, such as Delphi rounds, modified Delphi methods, consensus meetings, or iterative multistakeholder refinement. Among these, several represent EQUATOR-endorsed extensions (eg, CONSORT-AI, SPIRIT-AI [Standard Protocol Items – Recommendations for Interventional Trials – Artificial Intelligence Extension], Developmental and Evaluation Checklist for Decision Support Systems Driven by Artificial Intelligence [DECIDE-AI], CLAIM 2024, TRIPOD-Cluster [TRIPOD Extension for Clustered Data], TRIPOD + AI, CHEERS-AI [Consolidated Health Economic Evaluation Reporting Standards for Interventions That Use Artificial Intelligence], and STARD-AI). An additional 13% (6/46) were derived from systematic or scoping reviews. Only 2 frameworks (4.3%), APPRAISE-AI (Assessment of Predictive Performance and Bias in Artificial Intelligence Studies) and the AI-CDSS success framework, underwent empirical validation procedures, including reliability testing, construct validity assessment, or psychometric modeling.

Structural formats also varied: 65% (30/46) of the documents followed an IMRD-compliant structure. Across all frameworks, recurring domains included dataset description, model development procedures, performance metrics, internal and external validation strategies, reproducibility requirements, and overall reporting transparency. Several frameworks were specialty-specific, addressing fields such as dermatology, dentistry, cardiology, urology, or interventional radiology.

Importantly, 52.2% (24/46) of the documents corresponded to official extensions of established reporting guidelines (PRISMA or EQUATOR) or to independently implemented frameworks aligned with these standards.

Taken together, the included documents reveal a rapidly expanding and increasingly structured landscape of AI evaluation frameworks, characterized by high levels of interdisciplinarity, growing methodological convergence, and a clear movement toward formalized reporting standards designed to enhance transparency, reproducibility, and clinical applicability in AI-based health care research.

### Domain 2: Validation and Performance Aspects

Two documents were not included in Table 2 because they addressed exclusively ethical considerations and did not provide evaluative components related to technical performance, clinical effectiveness, or clinical purpose.

**Table 2.** Characterization of the utility of AI evaluation frameworks applied in clinical practice.

Author, Year, and Reference	Objective	Technical performance	Clinical effectiveness	Generalizability	Clinical purpose
Luo et al, 2016 [28]	Investigative	It does not specify the parameters	It does not specify the parameters	Complete	Prediction
Floridi et al, 2018 [29]	Investigative	Not reported	Not reported	Not reported	Not reported
Reps et al, 2018 [30]	Clinical utility	AUC^a^	Sensitivity, specificity	Incomplete	Prediction
Cruz Rivera et al, 2020 [31]	Investigative	Not reported	Not reported	Not reported	Diagnostic
Hernandez-Boussard et al, 2020 [32]	Clinical utility	Not reported	Not reported	Complete	Diagnostic and prediction
Liu et al, 2020 [33]	Investigative	Not reported	Not reported	Not reported	Diagnostic
Mongan et al, 2020 [34]	Investigative	Sensitivity, specificity, AUC, PPV^b^, NPV^c^, and calibration accuracy	Not reported	Complete	Prediction
Norgeot et al, 2020 [35]	Investigative	*F*_1_-scores, Dice coefficient, or AUC	Sensitivity, specificity, PPV, NPV, NNT^d^, and AUC	Complete	Not reported
Sengupta et al, 2020 [36]	Investigative	AUC	Not reported	Incomplete	Not reported
Stevens et al, 2020 [37]	Investigative	AUC	Not reported	Incomplete	Prediction
Young et al, 2020 [38]	Investigative	Not reported	Not reported	Not reported	Not reported
Cabitza and Campagner, 2021 [39]	Investigative	Sensitivity, specificity, and AUC	PPV, NPV^c^, and accuracy	Not reported	Not reported
Ji et al, 2021 [40]	Clinical utility	Not reported	Not reported	Not reported	Not reported
Olczak et al, 2021 [41]	Clinical utility	Exactitude, sensitivity, specificity, AUC, others (*F*_1_-score or Dice score)	Calibration accuracy, PPV and NPV, among others	Wrong	Diagnostic and Prediction
Schwendicke et al, 2021 [42]	Investigative	Sensitivity, specificity, and AUC	Not reported	Wrong	Not reported
Bazoukis et al, 2022 [43]	Investigative	Not reported	Not reported	Not reported	Not reported
Daneshjou et al, 2022 [44]	Investigative	Accuracy and free-response ROC^e^	Not reported	Incomplete	Diagnostic
Fusar-Poli et al, 2022 [45]	Investigative	Not reported	Not reported	Not reported	Diagnostic and prediction
Kwong et al, 2021 [46]	Investigative	Sensitivity, PPV, and AUC	Not reported	Complete	Diagnostic and prediction
Lu et al, 2022 [47]	Investigative	Not reported	Not reported	Not reported	Not reported
Vasey et al, 2022 [49]	Investigative	Not reported	Not reported	Not reported	Not reported
Abdulazeem et al, 2023 [50]	Investigative	Not reported	AUROC^f^, sensitivity, specificity, predictive values, accuracy	Not reported	Diagnostic and prediction
Cacciamani et al, 2023 [15]	Investigative	Not reported	Not reported	Not reported	Not reported
Debray et al, 2023 [51]	Investigative	Not reported	Not reported	Wrong	Prediction
Elvidge et al, 2023 [52]	Investigative	Not reported	Not reported	Not reported	Not reported
Klement et al, 2023 [53]	Investigative	Not reported	Not reported	Not reported	Diagnostic and prediction
Kwong et al, 2023 [54]	Investigative	Not reported	Not reported	Not reported	Prediction
Murphy et al, 2023 [55]	Investigative	AUC	Sensitivity and specificity	Not reported	Not reported
Collins et al, 2024 [56]	Investigative	Not reported	Not reported	Not reported	Diagnostic and prediction
Elfer et al, 2024 [57]	Investigative	Not reported	Not reported	Not reported	Diagnostic
El Emam et al, 2024 [58]	Investigative	Not reported	Not reported	Not reported	Diagnostic and prediction
Guni et al, 2024 [59]	Investigative	Not reported	Not reported	Not reported	Not reported
Kapoor et al, 2024 [60]	Investigative	Not reported	Not reported	Not reported	Diagnostic and prediction
Labkoff et al, 2024 [61]	Investigative	Not reported	Not reported	Not reported	Not reported
Masters and Salcedo, 2024 [62]	Investigative	Not reported	Not reported	Not reported	Not reported
Ray et al, 2024 [63]	Investigative	AUC	Sensitivity, specificity, accuracy, and precision	Not reported	Diagnostic
Tejani et al, 2024 [13]	Investigative	Sensitivity	Not reported	Incomplete	Diagnostic and prediction
Uribe et al, 2024 [64]	Investigative	Not reported	Not reported	Not reported	Not reported
Warren et al, 2024 [65]	Investigative	Not reported	Not reported	Not reported	Not reported
Kalaycioglu et al, 2025 [66]	Investigative	Not reported	HF 30-d mortality; AMI^g^ in-hospital death	Not reported	Prediction
PAHO^h^ 2025 [67]	Investigative	Not reported	Not reported	Not reported	Not reported
Sounderajah et al, 2025 [14]	Investigative	Not reported	Not reported	Not reported	Diagnostic
Tuygunov et al, 2025 [68]	Not reported	Not reported	Not reported	Not reported	Not reported
Wang et al, 2025 [69]	Investigative	Not reported	Not reported	Not reported	Not reported

a AUC: area under the curve.

b PPV: positive predictive value.

c NPV: negative predictive value.

d NNT: number needed to treat.

e ROC: receiver operating characteristic.

f AUROC: area under the receiver operating characteristic curve.

g AMI: acute myocardial infarction.

h PAHO: Pan American Health Organization.

The evaluation frameworks revealed substantial heterogeneity in how studies articulate their objectives, define clinical purpose, and report technical or clinical performance parameters. Most frameworks were investigational in nature (approximately 88%, 39/44), whereas only 4 frameworks explicitly stated a focus on assessing clinical utility (eg, Reps et al [30], Hernandez-Boussard et al [32], Ji et al [40], and Olczak et al [41]). This distribution indicates that most published frameworks remain exploratory rather than implementation-oriented.

Across studies, technical performance reporting was inconsistent. Only 31.8% (14/44) provided at least 1 performance metric. The most frequently reported indicators were AUC or ROC values, present in 12/44 studies, followed by sensitivity and specificity. Less commonly, frameworks used positive predictive values and negative predictive values, accuracy, or segmentation metrics such as the *F*_1_-score or Dice coefficient (eg, Norgeot et al [35] and Olczak et al [41]). A small subset incorporated measures of calibration accuracy, number needed to treat, or task-specific metrics (eg, 30-day mortality in Kalaycıoğlu et al [66]).

Reporting of clinical effectiveness metrics was even more limited. Only 7 (15.9%) studies explicitly referenced indicators of clinical performance, most commonly predictive values, accuracy, or calibration. The overwhelming majority did not report any clinical effectiveness parameters, suggesting that most frameworks remain focused on model development and internal performance rather than real-world clinical applicability.

When classifying the rigor of evaluation (technical performance), only 5 (11.4%) frameworks were categorized as providing a complete evaluation, integrating internal and external validation components (eg, Luo et al [28], Hernandez-Boussard et al [32], Mongan et al [34], Norgeot et al [35], Kwong et al 2021, and Debray et al [51]). An additional 5 (11.4%) studies were judged as incomplete, and 3 studies were rated as incorrect (“wrong”) due to methodological inconsistencies or misalignment between reported metrics and intended clinical purpose.

Regarding clinical purpose, approximately one-third (9/44) of the frameworks focused on diagnostic applications, while 7 of 44 addressed prediction. Another subset (7/44) incorporated both diagnostic and prognostic applications.

### Domain 3: Ethical Alignment With UNESCO Principles

**Figure 2.**
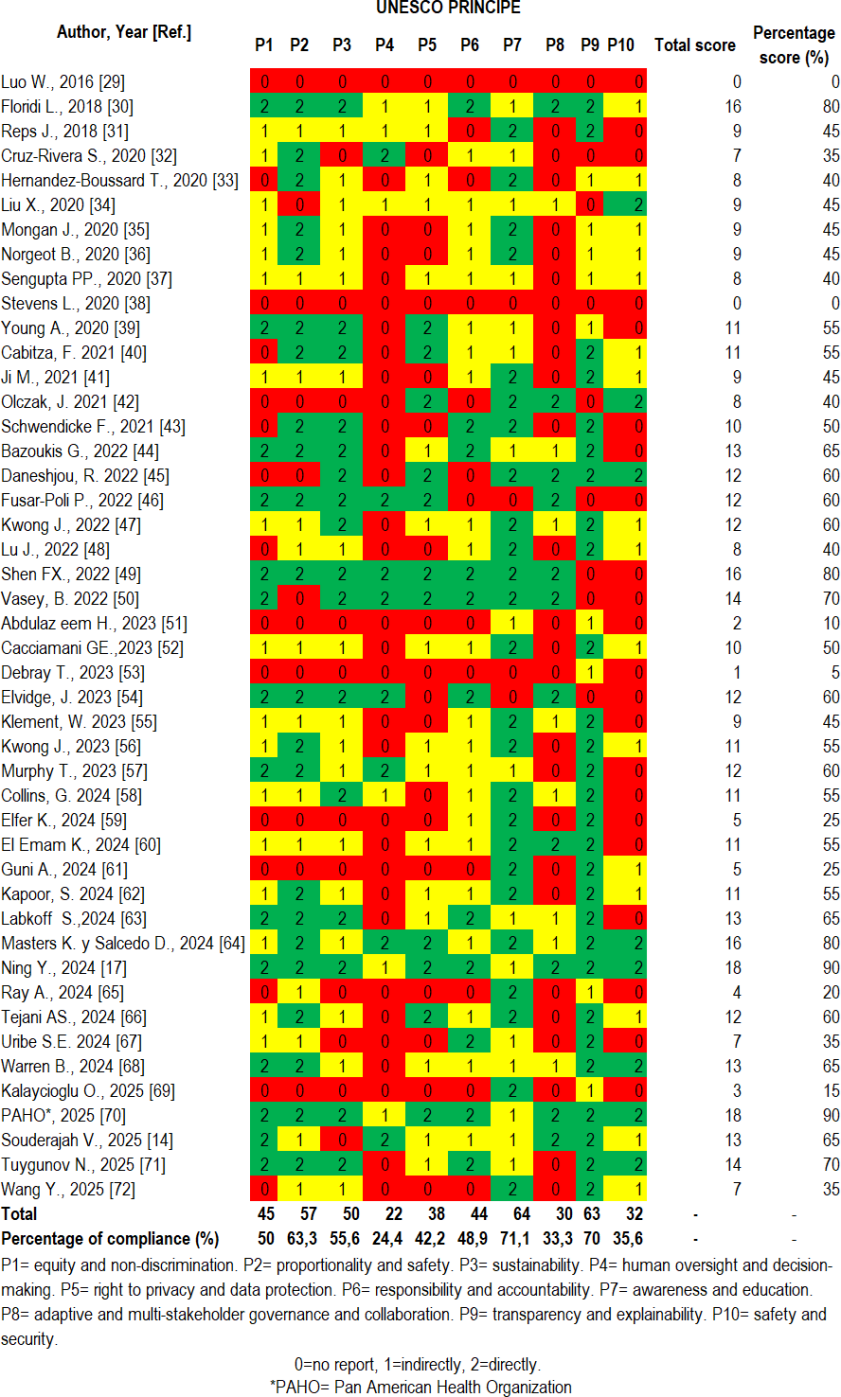
Percentage of compliance with United Nations Educational, Scientific and Cultural Organization ethical principles across AI evaluation frameworks [13-15,17,28-69]. P1: equity and nondiscrimination; P2: proportionality and safety; P3: sustainability; P4: human oversight and decision-making; P5: right to privacy and data protection; P6: responsibility and accountability; P7: awareness and education; P8: adaptive and multistakeholder governance and collaboration; P9: transparency and explainability; P10: safety and security; 0: no report; 1: indirectly; 2: directly; PAHO: Pan American Health Organization; UNESCO: United Nations Educational, Scientific and Cultural Organization.

Analysis of the ethical dimensions of the included frameworks revealed substantial variability in their alignment with the 10 UNESCO AI ethical principles. Overall compliance was modest, with only a small group of documents achieving high scores (≥80%), including works by Floridi et al [29], Shen et al [48], Masters and Salcedo [62], Ning et al [17], and the PAHO [67], each demonstrating broad and explicit ethical integration. In contrast, four documents (Luo et al [28], Stevens et al [37], Abdulazeem et al [50], and Debray et al [51]) showed no or minimal ethical reporting, scoring below 10%. Across principles, the highest levels of adherence were observed for awareness and education (P7, 71.1%), transparency and explainability (P9, 70%), and proportionality and safety (P2, 63.3%), indicating a stronger emphasis on communicability, system clarity, and risk mitigation. Conversely, human oversight (P4, 24.4%) and adaptive, multistakeholder governance (P8, 33.3%) were the least addressed, reflecting limited incorporation of human-in-the-loop safeguards and participatory governance structures. Only a minority of frameworks exhibited balanced coverage across all principles, revealing a fragmented ethical landscape where most documents address isolated ethical components rather than offering comprehensive, principle-wide guidance (Figure 2 [13-15,17,28-69]).

**Figure 3.**
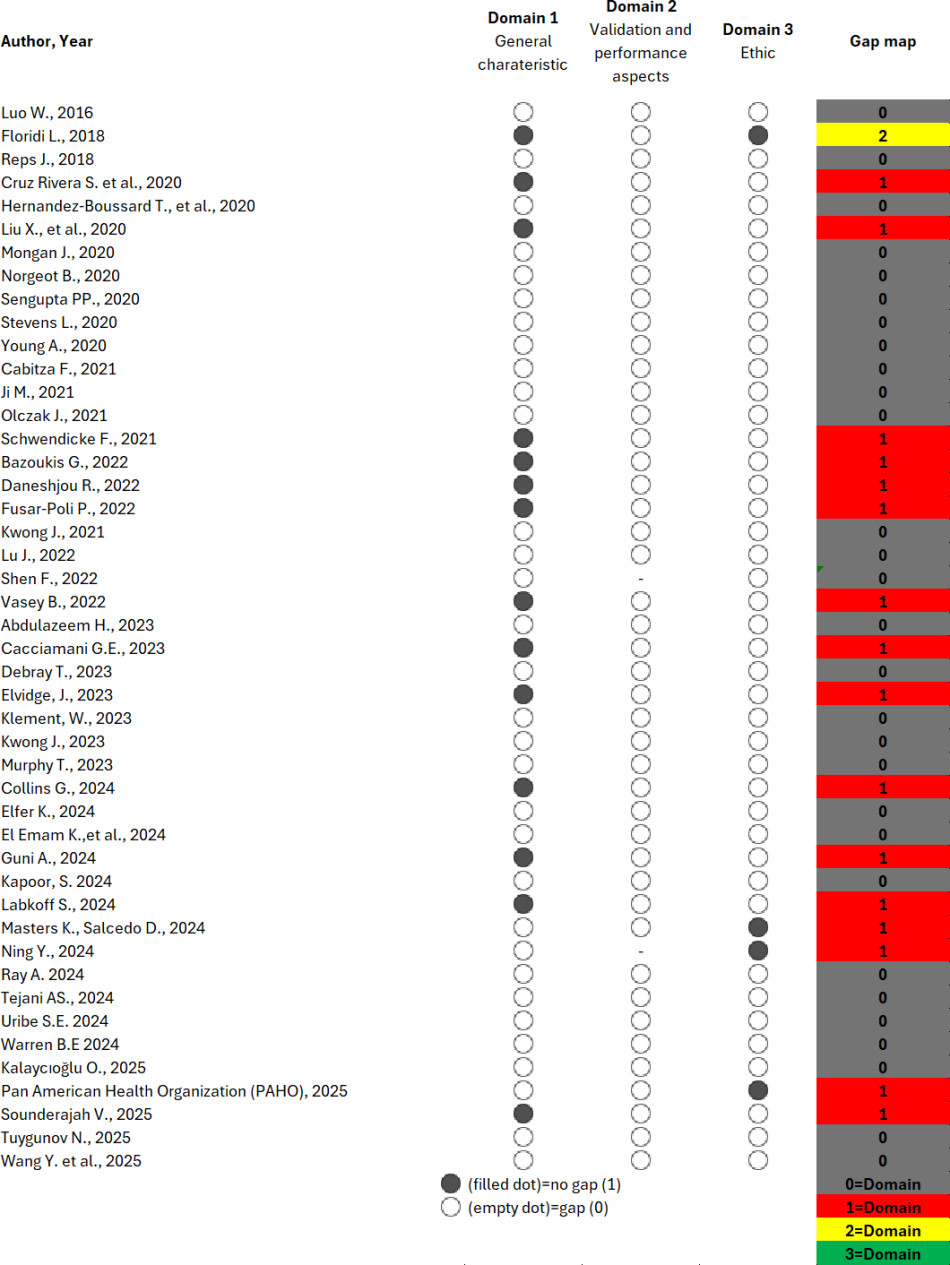
Gap map of evaluation frameworks for clinical AI across three domains: general characteristics, validation and performance, and ethical integration. Dot plot representation indicates the presence (1) or absence (0) of gaps according to predefined criteria [13-15,17,28-69].

The gap map presented in Figure 3 [13-15,17,28-69] shows that most existing AI evaluation frameworks primarily emphasize methodological and technical aspects of model performance. In contrast, comparatively less attention is given to clinical applicability and ethical considerations. The distribution of data points across the 3 evaluated domains suggests that these frameworks tend to prioritize reporting transparency and reproducibility, while aspects related to clinical utility, external validation, and ethical safeguards for real-world implementation remain insufficiently addressed.

Across the three domains evaluated (general characteristics, validations and performance aspects, and ethics), most frameworks presented substantial gaps. Specifically, 63% (29/46) of frameworks showed gaps across all 3 domains. In contrast, 34.8% (16/46) of the frameworks fully complied with the criteria in at least 1 domain. Among these, 3 frameworks met the criteria for the ethics domain (Floridi et al [29], Masters and Salcedo [62], and Ning et al [17]), while 13 complied with the general characteristics domain. Only 2.2% (1/46) of the frameworks met the high-quality criteria in 2 domains (general characteristics and ethics). Importantly, none of the evaluated frameworks met the high-quality criteria for the validation and performance aspects domain, and no framework fulfilled the criteria across all 3 domains simultaneously.

## Discussion

This scoping review identified a fragmented landscape of evaluation frameworks for clinical AI, characterized by heterogeneous methodological approaches, limited integration of external validation aligned with intended clinical use, and inconsistent incorporation of ethical principles. These findings directly address the study objectives by highlighting gaps in methodological rigor, real-world applicability, and ethical alignment. Ethical integration was inconsistently addressed, with most frameworks covering only a limited subset of UNESCO ethical domains.

These findings can be better understood in the context of the rapid expansion of methodological and reporting frameworks for clinical AI [6]. This pattern is consistent with the orientation of several influential frameworks included in this review, many of which were developed primarily to improve transparency, reproducibility, and completeness of reporting, while providing more limited coverage of implementation-related issues [72]. Collectively, this proliferation of frameworks reflects a broad consensus regarding the need to promote transparency, rigor, and reproducibility in medical AI research [29,34,49]. Minimum Information for Medical AI Reporting (MINIMAR), for example, was proposed to improve transparent reporting of the design, development, evaluation, and validation of medical AI models, with explicit emphasis on replication, external validation, and the identification of potential biases and unintended consequences [32]. Likewise, Minimum Information about Clinical Artificial Intelligence Modeling (MI-CLAIM) was designed to enable assessment of clinical impact, fairness, and bias while facilitating replication of the technical design process of clinical AI studies [35]. In the same direction, Klement and El Emam [53] showed that existing high-quality guidelines did not individually provide complete coverage, supporting the fragmented landscape identified in our gap map. However, our findings suggest that this expansion has occurred without corresponding consolidation of validation standards or clinical applicability.

The predominance of technical and reporting-oriented domains over broader clinical dimensions in our review also aligns with previous literature showing that strong model performance does not necessarily translate into clinical benefit. Lu et al [47] found inconsistent adherence to reporting recommendations in deployed clinical prediction models, particularly for reliability-related items such as external validation, uncertainty, monitoring, fairness, and transparency. This broader concern is also reflected in DECIDE-AI, which notes that strong performance in preclinical studies has not yet been matched by high-quality evidence of improved clinician performance or patient outcomes in clinical settings [49]. Taken together, these findings suggest that current evaluation of clinical AI remains more mature at the level of methodological description than at the level of demonstrated clinical usefulness [46,48].

A related implication is that methodological rigor in clinical AI should not be interpreted narrowly as internal validation or reporting compliance. SPIRIT-AI and CONSORT-AI both emphasize that AI interventions should be described in relation to their intended use, intended users, integration into the clinical pathway, required expertise, handling of input data, output interpretation, and the way outputs contribute to downstream clinical decision-making [31,33]. CONSORT-AI also requires reporting of performance-error analysis and highlights the importance of documenting access to the intervention or its code, while SPIRIT-AI recommends explicit planning for performance-error identification and description of implementation requirements in the trial setting [31,33]. Together, these frameworks support the view that robust evaluation of clinical AI requires attention not only to methodological transparency but also to usability, context of use, and conditions for safe implementation.

Our finding that real-world applicability was less consistently represented is also supported by frameworks explicitly designed to address translational stages of evaluation [73,74]. DECIDE-AI was developed as a stage-specific reporting guideline for early, small-scale, live clinical evaluation and focuses on proof of clinical utility, safety, human factors, and preparation for larger-scale studies [49]. Its rationale explicitly refers to the gap between mathematical performance and clinical utility and to the need to address challenges such as dataset shift, user variability, and implementation in live clinical settings [49]. More recently, Labkoff et al [61] argued that responsible AI-enabled clinical decision support requires a more comprehensive framework spanning model training, explainability, validation, certification, monitoring, continuous evaluation, privacy, fairness, and regulatory oversight. These proposals are consistent with the pattern observed in our review: implementation-relevant domains are increasingly recognized in the literature, but they remain unevenly operationalized across available frameworks.

The limited number of transdisciplinary studies in our review is also noteworthy. Some of the more mature frameworks were developed through broad multistakeholder processes. DECIDE-AI was produced through an international consensus process involving multiple stakeholder groups [49] and Labkoff et al [61] similarly reported a consensus process including clinicians, software developers, academics, ethicists, attorneys, policy experts, scientists, and patients. However, our mapping suggests that such breadth of stakeholder participation is not yet typical across the field. This matters because clinical AI is not only a technical intervention, but also a clinical, organizational, ethical, and regulatory one. Frameworks developed from narrower perspectives may therefore be less able to capture the full conditions needed for trustworthy implementation [75].

The ethical findings in this review are likewise consistent with the literature included in the scoping review. Floridi et al [29] synthesized major AI ethics initiatives around beneficence, nonmaleficence, autonomy, justice, and explicability, arguing that explicability combines intelligibility and accountability and is necessary to make the other principles actionable. Their discussion of justice also extends beyond discrimination to shared benefit, equal access, and protection of social structures such as health care systems [29]. In more applied health care literature, Shen et al [48] proposed an ethics checklist structured around informed consent, equity, diversity and access, privacy and partnerships, regulation and law, return of results, and duty to warn and report [48]. Ning et al [17] similarly proposed the TREGAI (Transparent Reporting of Ethics for Generative Artificial Intelligence) checklist for generative AI in health care, derived from a scoping review and intended to operationalize ethical evaluation within practical guidance. These studies support our interpretation that ethical concerns are clearly present in the literature, but are not yet incorporated with comparable depth or consistency across clinical AI evaluation frameworks. This limitation is particularly relevant for clinical implementation, as insufficient ethical integration may undermine patient safety, public trust, and regulatory acceptance of AI systems in health care.

A similar pattern can be seen in more implementation-oriented proposals. Bazoukis et al [43] describe a regulatory framework for augmented intelligence in medicine that includes accountability, liability, equity and inclusion, transparency, explainability, education, patient engagement, cybersecurity and privacy, ethics and fairness, as well as safety and postmarket surveillance. Labkoff et al [61] also emphasize transparency, documentation, training, validation, certification, monitoring, safety reporting, fairness, and privacy as necessary elements of responsible AI-enabled clinical decision support. In our review, however, these ethical- and governance-related elements were not uniformly represented across frameworks. This suggests that the current landscape remains fragmented: ethical principles are increasingly acknowledged, but their operational integration into evaluation standards is still incomplete.

Taken together, the three domains examined in this scoping review reveal a recurrent pattern: methodological rigor, clinical applicability, and ethical integration do not consistently converge within individual frameworks. Many frameworks are strong in reporting transparency or technical assessment, but are less explicit regarding external validation, implementation conditions, real-world use, or comprehensive ethical governance. This fragmentation appears to be a structural feature of the current evaluative landscape rather than a weakness of any single framework. It likely reflects the parallel evolution of technical reporting, clinical evaluation, and ethical guidance in AI, which have not yet been fully harmonized within a single evaluative model [29,31-35,43,49,53,61].

### Limitations

As with any scoping review, this study has inherent limitations. First, as a scoping review, its purpose was to map and characterize the available literature rather than to compare the superiority or effectiveness of individual frameworks [19,76]. Therefore, the findings should be interpreted as an overview of patterns, emphases, and gaps in the field, not as evidence that one framework performs better than another. Second, the included publications were heterogeneous in scope, purpose, and intended stage of use, ranging from reporting guidelines and consensus statements to conceptual ethics papers and implementation-oriented proposals [29,31,33,34,49,56]. Although this heterogeneity is informative for evidence mapping, it limits direct comparability across sources [15,34,40,45,46].

Third, the gap map required interpretive judgment when classifying whether a framework addressed specific methodological, clinical, or ethical domains, particularly when such issues were mentioned implicitly rather than operationalized explicitly [37,50,51]. For that reason, the categorizations presented here should be understood as a structured synthesis of the literature rather than a definitive ranking. Finally, many of the included publications propose how clinical AI should be evaluated, but do not prospectively test the effect of those frameworks in routine clinical settings [40,54,77]. Accordingly, this review is stronger in identifying what the literature currently prioritizes and omits than in determining how well these frameworks perform in practice [49,61].

### Conclusions

In conclusion, this scoping review indicates that current evaluation and reporting frameworks for clinical AI are strongest in technical and reporting-oriented domains, but less consistently address broader validation strategies, real-world applicability, and comprehensive ethical integration. This interpretation is consistent with the included literature, which has emphasized transparency, reproducibility, intended use, validation, fairness, and safety, while also recognizing the persistent gap between algorithm development and responsible implementation in clinical settings.

The broader implication is that advancing clinical AI will require a shift from fragmented, reporting-oriented approaches toward integrated evaluation frameworks that align methodological rigor, real-world validation, and ethical governance. Future frameworks should move beyond checklist-based reporting to support implementation, regulatory decision-making, and continuous monitoring in clinical settings. Without such integration, the translation of AI innovations into routine health care practice will remain limited.

## Supplementary material

10.2196/78168 Multimedia Appendix 1 Additional methodological materials and extracted data supporting the scoping review.

10.2196/78168 Multimedia Appendix 2 Excluded documents.

10.2196/78168 Checklist 1 PRISMA-ScR checklist.

10.2196/78168 Checklist 2 PRISMA 2020 Abstract checklist.

## Abbreviations

APPRAISE-AI: Assessment of Predictive Performance and Bias in Artificial Intelligence Studies
AUC: area under the curve
CHEERS-AI: Consolidated Health Economic Evaluation Reporting Standards for Interventions That Use Artificial Intelligence
CLAIM: Checklist for Artificial Intelligence in Medical Imaging
CONSORT-AI: Consolidated Standards of Reporting Trials for Artificial Intelligence
DECIDE-AI: Developmental and Evaluation Checklist for Decision Support Systems Driven by Artificial Intelligence
EQUATOR: Enhancing the Quality and Transparency of Health Research
FROC: free-response receiver operating characteristic
IMRD: Introduction, Methods, Results, and Discussion
MI-CLAIM: Minimum Information about Clinical Artificial Intelligence Modeling
MINIMAR: Minimum Information for Medical AI Reporting
PRISMA: Preferred Reporting Items for Systematic Reviews and Meta-Analyses
PRISMA-AI: Preferred Reporting Items for Systematic Reviews and Meta-Analyses extension for Artificial Intelligence
PRISMA-S: Preferred Reporting Items for Systematic reviews and Meta-Analyses literature search extension
PRISMA-ScR: Preferred Reporting Items for Systematic Reviews and Meta-Analyses extension for Scoping Reviews
ROC: receiver operating characteristic
SPIRIT-AI: Standard Protocol Items – Recommendations for Interventional Trials – Artificial Intelligence Extension
STARD-AI: Standards for Reporting of Diagnostic Accuracy – Artificial Intelligence
TREGAI: Transparent Reporting of Ethics for Generative Artificial Intelligence
TRIPOD+AI: Transparent Reporting of Multivariable Prediction Models with Artificial Intelligence
TRIPOD-Cluster: TRIPOD Extension for Clustered Data
UNESCO: United Nations Educational, Scientific and Cultural Organization
