# Supplementary material for: Evaluation Frameworks for Clinical AI Incorporating Validation Strategies, Real-World Applicability, and Ethical Principles: Scoping Review
Source: J Med Internet Res. 2026 Jul 22;28:e78168. doi: 10.2196/78168 (PMC13392654; doi:10.2196/78168)
Supplement: Multimedia Appendix 1 [file jmir-v28-e78168-s001.docx]

**Table S1.** Equivalent adaptations of this strategy.

| **Database** | **Date** | **Search strategy** | **Filters** | **Observation** |
| --- | --- | --- | --- | --- |
| Pubmed | 28/02/2026 | ("Artificial Intelligence"[Mesh] OR "Artificial Intelligence"[tiab] OR "Machine Learning"[Mesh] OR "Machine Learning"[tiab] OR "Deep Learning"[tiab] OR "Neural Networks, Computer"[Mesh] OR "Neural Network*"[tiab] OR "Natural Language Processing"[Mesh] OR "Natural Language Processing"[tiab] OR "Generative AI"[tiab] OR "Explainable AI"[tiab]) AND ("Validation Studies as Topic"[Mesh] OR "Validation"[tiab] OR "External Validation"[tiab] OR "Model Validation"[tiab] OR "Generalizability"[tiab] OR "Calibration"[tiab] OR "Performance Metrics"[tiab] OR "Reproducibility of Results"[Mesh] OR "Overfitting"[tiab]) AND ("Decision Support Systems, Clinical"[Mesh] OR "Clinical Decision Support"[tiab] OR "Clinical Decision-Making"[Mesh] OR "Predictive Models"[Mesh] OR "Prediction Model*"[tiab] OR "Prognostic Model*"[tiab] OR "Risk Prediction"[tiab] OR "Diagnostic Accuracy"[tiab] OR "Computer-Assisted Diagnosis"[Mesh]) AND ("Ethics, Medical"[Mesh] OR "Bioethics"[Mesh] OR "Ethic*"[tiab] OR "Equity"[tiab] OR "Transparency"[tiab] OR "Accountability"[tiab] OR "Informed Consent"[tiab] OR "Patient Autonomy"[tiab] OR "Privacy"[tiab] OR "Confidentiality"[tiab] OR "Responsible AI"[tiab] OR "Evaluation Framework*"[tiab] OR "Reporting Guideline*"[tiab] OR "CONSORT-AI"[tiab] OR "SPIRIT-AI"[tiab] OR "TRIPOD-AI"[tiab] OR "STARD-AI"[tiab] OR "CLAIM"[tiab]) | Humans | MeSH + keywords |
| EBSCOhost | 28/02/2026 | (TITLE-ABS-KEY("artificial intelligence" OR "machine learning" OR "deep learning" OR "neural network*" OR "natural language processing" OR "generative ai" OR "explainable ai")) AND (TITLE-ABS-KEY("validation" OR "external validation" OR "model validation" OR "generalizability" OR "calibration" OR "performance metrics" OR "reproducibility" OR "overfitting")) AND (TITLE-ABS-KEY("clinical decision support" OR "clinical decision support system*" OR "predictive model*" OR "prognostic model*" OR "risk prediction" OR "diagnostic accuracy" OR "computer assisted diagnosis")) AND (TITLE-ABS-KEY("ethics" OR "bioethics" OR "equity" OR "transparency" OR "accountability" OR "informed consent" OR "autonomy" OR "privacy" OR "confidentiality" OR "responsible ai" OR "evaluation framework*" OR "reporting guideline*" OR "consort ai" OR "spirit ai" OR "tripod ai" OR "stard ai" OR "claim")) |  | Keywords TITLE-ABS-KEY: |
| Sage | 28/02/2026 | (TITLE-ABS-KEY("artificial intelligence" OR "machine learning" OR "deep learning" OR "neural network*" OR "natural language processing" OR "generative ai" OR "explainable ai")) AND (TITLE-ABS-KEY("validation" OR "external validation" OR "model validation" OR "generalizability" OR "calibration" OR "performance metrics" OR "reproducibility" OR "overfitting")) AND (TITLE-ABS-KEY("clinical decision support" OR "clinical decision support system*" OR "predictive model*" OR "prognostic model*" OR "risk prediction" OR "diagnostic accuracy" OR "computer assisted diagnosis")) AND (TITLE-ABS-KEY("ethics" OR "bioethics" OR "equity" OR "transparency" OR "accountability" OR "informed consent" OR "autonomy" OR "privacy" OR "confidentiality" OR "responsible ai" OR "evaluation framework*" OR "reporting guideline*" OR "consort ai" OR "spirit ai" OR "tripod ai" OR "stard ai" OR "claim")) |  | Keywords TITLE-ABS-KEY: |
| ProQuest central | 28/02/2026 | (“Artificial Intelligence” OR “Machine Learning” OR “Deep Learning”) AND (“Validation” OR “External Validation” OR “Generalizability” OR “Calibration”) AND (“Clinical Decision Support” OR “Predictive Models” OR “Risk Prediction”) AND  (“Ethics” OR “Transparency” OR “Guidelines” OR “Evaluation Frameworks”) | Main article OR Article OR Evidence-based healthcare OR Working papers/preliminary edition OR Literature review OR Review | MeSH + keywords |

**Table S2.** The bioethical evaluation of the included frameworks followed the UNESCO ethical principles for artificial intelligence.

| **Author, Year** | **Ethical aspects considered** | **Additional aspects according to UNESCO criteria** | | **Missing aspects according to UNESCO criteria=0** |
| --- | --- | --- | --- | --- |
|  |  | **Directly=2** | **Indirectly=1** |  |
| Luo et al, 2016 [1] | No reported | Not Reported | Not Reported | Equity and non-discrimination  Proportionality and safety  Sustainability  Human oversight and decision-making  Right to privacy and data protection  Responsibility and accountability  Awareness and education  Adaptive and multi-stakeholder governance and collaboration  Transparency and explainability  Safety and security |
| Floridi et al, 2018 [2] | Directly and indirectly | Human Dignity  Benefit and Non-maleficence  Justice  Social responsibility  Ethical assessment  Transparency and communication | Privacy and confidentiality  Autonomy and informed consent  Scientific integrity  Caution with emerging technologies | None |
| Reps et al, 2018 [3] | Directly and indirectly | Scientific integrity  Transparency and communication | Human Dignity  Benefit and Non-maleficence  Justice  Privacy and confidentiality | Responsibility and accountability  Adaptive and multi-stakeholder governance and collaboration  Safety and security |
| Cruz Rivera et al, 2020 [4] | Directly and indirectly | Informed Consent  Nonmaleficence and Beneficence | Human Dignity  Social Responsibility  Scientific Integrity | Sustainability  Right to privacy and data protection  Adaptive and multi-stakeholder governance and collaboration  Transparency and explainability  Safety and security |
| Hernandez-Boussard et al, 2020 [5] | Directly and indirectly | Beneficence and non-maleficence  Scientific Integrity | Justice  Privacy and Confidentiality  Transparency and Communication  Caution with Emerging Technologies | Equity and non-discrimination  Human oversight and decision-making  Responsibility and accountability  Adaptive and multi-stakeholder governance and collaboration |
| Liu et al, 2020 [6] | Directly and indirectly | Safety and Security | Human Dignity  Autonomy and informed consent  Privacy and Confidentiality  Justice  Social responsibility  Scientific integrity  Ethical evaluation | Proportionality and safety  Transparency and explainability |
| Mongan et al, 2020 [7] | Directly and indirectly | Benefit and Non-maleficence  Scientific Integrity | Human Dignity  Justice  Social Responsibility  Transfer and Communication  Caution with Technology | Human oversight and decision-marking  Right to privacy and data protection  Adaptive and multi-stakeholder governance and collaboration |
| Norgeot et al, 2020 [8] | Directly and indirectly | Benefit and Non-maleficence  Scientific Integrity | Human Dignity  Justice  Social Responsibility  Transfer and Communication  Caution with Technology | Human oversight and decision-marking  Right to privacy and data protection  Adaptive and multi-stakeholder governance and collaboration |
| Sengupta et al, 2020 [9] | Indirectly | Not Reported | Human dignity  Beneficence and non-maleficence  Justice  Privacy and confidentiality  Social responsibility  Scientific integrity  Transparency and communication  Caution with emerging technologies | Human oversight and decision-marking  Adaptive and multi-stakeholder governance and collaboration |
| Stevens et al, 2020 [10] | Not Reported | Not Reported | Not Reported | Equity and non-discrimination  Proportionality and safety  Sustainability  Human oversight and decision-making  Right to privacy and data protection  Responsibility and accountability  Awareness and education  Adaptive and multi-stakeholder governance and collaboration  Transparency and explainability  Safety and security |
| Young et al, 2020 [11] | Directly and indirectly | Human Dignity  Benefit and Non-maleficence  Justice  Privacy and confidentiality | Social Responsibility  Scientific Integrity  Transparency and communication | Human oversight and decision-marking  Adaptive and multi-stakeholder governance and collaboration  Safety and security |
| Cabitza et al, 2021 [12] | Directly and indirectly | Beneficence and Nonmaleficence  Justice  Privacy and Confidentiality  Transparency and Communication | Caution with Technology | Equity and non-discrimination  Human oversight and decision-marking  Adaptive and multi-stakeholder governance and collaboration |
| Ji et al, 2021 [13] | Directly and indirectly | Scientific Integrity  Transparency and communication | Human Dignity  Benefit and Non-maleficence  Justice  Social Responsibility  Caution with emerging technologies | Human oversight and decision-marking  Right to privacy and data protection  Adaptive and multi-stakeholder governance and collaboration |
| Olczak et al, 2021 [14] | Directly | Privacy and Confidentiality  Ethical Assessment  Scientific Integrity  Caution with Emerging Technologies | Not Reported | Equity and non-discrimination  Proportionality and safety  Sustainability  Human oversight and decision-making  Responsibility and accountability  Transparency and explainability |
| Schwendicke et al, 2021 [15] | Directly | Benefit and Non-maleficence  Justice  Social Responsibility  Scientific Integrity  Transparency and communication | Not Reported | Equity and non-discrimination  Human oversight and decision-making  Right to privacy and data protection  Adaptive and multi-stakeholder governance and collaboration  Safety and security |
| Bazoukis et al, 2022 [16] | Directly and indirectly | Human Dignity  Benefit and Non-maleficence  Justice  Social Responsibility  Transparency and communication | Privacy and confidentiality  Scientific Integrity  Ethical assessment | Human oversight and decision-making  Safety and security |
| Daneshjou et al, 2022 [17] | Directly | Justice  Privacy and Confidentiality  Scientific Integrity  Ethical Assessment  Transparency and Communication  Caution with Emerging Technologies | Not Reported | Equity and non-discrimination  Proportionality and safety  Human oversight and decision-making  Responsibility and accountability |
| Fusar-Poli et al, 2022 [18] | Directly | Human Dignity  Benefit and Non-maleficence  Justice  Autonomy and informed consent  Privacy and confidentiality  Ethical assessment | Not Reported | Right to privacy and data protection  Responsibility and accountability  Awareness and education  Transparency and explainability  Safety and security |
| Kwong et al, 2021 [19] | Directly and indirectly | Justice  Scientific Integrity  Transparency and Communication | Human Dignity  Benefit and Non-Maleficence  Privacy and Confidentiality  Social Responsibility  Ethical Assessment  Caution with Emerging Technologies | Human oversight and decision-making |
| Lu et al, 2022 [20] | Directly and indirectly | Scientific Integrity  Transparency and communication | Benefit and Non-maleficence  Justice  Social Responsibility  Caution with emerging technologies | Equity and non-discrimination  Human oversight and decision-making  Right to privacy and data protection  Adaptive and multi-stakeholder governance and collaboration |
| Shen et al, 2022 [21] | Directly | Human dignity  Benefit and non-maleficence  Autonomy Informed consent  Justice  Privacy and confidentiality  Social responsibility  Ethical evaluation  Scientific integrity | Not Reported | Transparency and explainability  Safety and security |
| Vasey et al, 2022 [22] | Directly | Human dignity  Informed consent and autonomy  Justice  Privacy and confidentiality  Social responsibility  Ethical evaluation  Scientific integrity | Not Reported | Proportionality and safety  Transparency and explainability  Safety and security |
| Abdulazeem et al, 2023 [23] | Indirectly | Not Reported | Scientific Integrity  Transparency and communication | Equity and non-discrimination  Proportionality and safety  Sustainability  Human oversight and decision-making  Right to privacy and data protection  Responsibility and accountability  Adaptive and multi-stakeholder governance and collaboration  Safety and security |
| Cacciamani et al, 2023 [24] | Directly and indirectly | Scientific Integrity  Transparency and Communication | Human Dignity  Benefit and Non-Maleficence  Justice  Privacy and Confidentiality  Ethical Assessment  Social Responsibility  Caution with Emerging Technologies | Human oversight and decision-making  Adaptive and multi-stakeholder governance and collaboration |
| Debray et al, 2023 [25] | Indirectly | Not Reported | Transparency and Communication | Equity and non-discrimination  Proportionality and safety  Sustainability  Human oversight and decision-making  Right to privacy and data protection  Responsibility and accountability  Awareness and education  Adaptive and multi-stakeholder governance and collaboration  Safety and security |
| Elvidge et al, 2023 [26] | Directly | Human Dignity  Nonmaleficence and Beneficence  Informed Consent and Autonomy  Justice  Social Responsibility  Ethical Assessment | No report | Right to privacy and data protection  Awareness and education  Transparency and explainability  Safety and security |
| Klement et al, 2023 [27] | Directly and indirectly | Scientific integrity  Transparency and communication | Human dignity  Benefit and non-maleficence  Justice  Social responsibility  Ethical evaluation | Human oversight and decision-making  Right to privacy and data protection  Safety and security |
| Kwong et al, 2023 [28] | Directly and indirectly | Benefit and Non-maleficence  Scientific integrity  Transparency and communication | Human dignity  Justice  Privacy and confidentiality  Social responsibility  Caution with emerging technologies | Human oversight and decision-making  Adaptive and multi-stakeholder governance and collaboration |
| Murphy et al, 2023 [29] | Directly and indirectly | Human Dignity  Benefit and Non-maleficence  Autonomy and informed consent  Transparency and communication | Justice  Privacy and confidentiality  Scientific Integrity  Social Responsibility | Adaptive and multi-stakeholder governance and collaboration  Safety and security |
| Collins et al, 2024 [30] | Directly and indirectly | Justice  Scientific integrity  Transparency and communication | Human dignity  Benefit and Non-maleficence  Autonomy and informed consent  Social responsibility  Ethical evaluation | Right to privacy and data protection  Safety and security |
| Elfer et al, 2024 [31] | Directly and indirectly | Scientific Integrity  Transparency and communication | Social Responsibility | Equity and non-discrimination  Proportionality and safety  Sustainability  Human oversight and decision-making  Right to privacy and data protection  Adaptive and multi-stakeholder governance and collaboration  Safety and security |
| El Emam et al, 2024 [32] | Directly and indirectly | Scientific integrity  Ethical evaluation  Transparency and communication | Human dignity  Benefit and non-maleficence  Justice  Social responsibility  Privacy and confidentiality | Human oversight and decision-making  Safety and security |
| Guni et al, 2024 [33] | Directly and indirectly | Scientific Integrity  Transparency and communication | Caution with emerging technologies | Equity and non-discrimination  Proportionality and safety  Sustainability  Human oversight and decision-making  Right to privacy and data protection  Responsibility and accountability  Adaptive and multi-stakeholder governance and collaboration |
| Kapoor et al, 2024 [34] | Directly and indirectly | Benefit and non-maleficence  Scientific integrity  Transparency and communication | Human dignity  Justice  Privacy and confidentiality  Social responsibility  Caution with emerging technologies | Human oversight and decision-making  Adaptive and multi-stakeholder governance and collaboration |
| Labkoff et al, 2024 [35] | Directly and indirectly | Human Dignity  Benefit and Non-maleficence  Justice  Social Responsibility  Transparency and communication | Privacy and confidentiality  Scientific Integrity  Ethical assessment | Human oversight and decision-making  Safety and security |
| Masters and Salcedo, 2024 [36] | Directly and indirectly | Benefit and nonmaleficence  Privacy and confidentiality  Autonomy and informed consent  Scientific integrity  Transparency and communication  Caution with emerging technologies | Human dignity  Justice  Social responsibility  Ethical evaluation | None |
| Ning et al, 2024 [37] | Directly and indirectly | Human Dignity  Benefit and Non-maleficence  Justice  Privacy and confidentiality  Social Responsibility  Ethical assessment  Transparency and communication  Caution with emerging technologies | Autonomy and informed consent  Scientific Integrity | None |
| Ray et al, 2024 [38] | Directly and indirectly | Scientific Integrity | Benefit and Non-maleficence  Transparency and communication | Equity and non-discrimination  Sustainability  Human oversight and decision-making  Right to privacy and data protection  Responsibility and accountability  Adaptive and multi-stakeholder governance and collaboration  Safety and security |
| Tejani et al, 2024 [39] | Directly and indirectly | Benefit and non-maleficence  Privacy and confidentiality  Scientific integrity  Transparency and communication | Human dignity  Justice  Social responsibility  Caution with emerging technologies | Human oversight and decision-making  Adaptive and multi-stakeholder governance and collaboration |
| Uribe et al, 2024 [40] | Directly and indirectly | Social Responsibility  Transparency and communication | Human Dignity  Benefit and Non-maleficence  Scientific Integrity | Sustainability  Human oversight and decision-making  Right to privacy and data protection  Adaptive and multi-stakeholder governance and collaboration  Safety and security |
| Warren et al, 2024 [41] | Directly and indirectly | Human Dignity  Benefit and Non-maleficence  Transparency and communication  Caution with emerging technologies | Justice  Privacy and confidentiality  Social Responsibility  Scientific Integrity  Ethical assessment | Human oversight and decision-making |
| Kalaycioglu et al, 2025 [42] | Directly and indirectly | Scientific Integrity | Transparency and communication | Equity and non-discrimination  Proportionality and safety  Sustainability  Human oversight and decision-making  Right to privacy and data protection  Responsibility and accountability  Adaptive and multi-stakeholder governance and collaboration  Safety and security |
| Pan American Health Organization (PAHO), 2025 [43] | Directly and indirectly | Human Dignity  Benefit and Non-maleficence  Justice  Privacy and confidentiality  Social Responsibility  Ethical assessment  Transparency and communication  Caution with emerging technologies | Autonomy and informed consent  Scientific Integrity | None |
| Sounderajah et al, 2025 [44] | Directly and indirectly | Equity and non-discrimination  Human oversight and decision-making  Adaptive and multi-stakeholder governance and collaboration  Transparency and explainability | Proportionality and safety  Right to privacy and data protection  Responsibility and accountability  Awareness and education  Safety and security | Sustainability |
| Tuygunov et al, 2025 [45] | Directly and indirectly | Human Dignity  Benefit and Non-maleficence  Justice  Social Responsibility  Transparency and communication  Caution with emerging technologies | Privacy and confidentiality  Scientific Integrity | Human oversight and decision-making  Adaptive and multi-stakeholder governance and collaboration |
| Wang et al, 2025 [46] | Directly and indirectly | Scientific Integrity  Transparency and communication | Benefit and Non-maleficence  Justice  Caution with emerging technologies | Equity and non-discrimination  Human oversight and decision-making  Right to privacy and data protection  Responsibility and accountability  Adaptive and multi-stakeholder governance and collaboration |

### **References**

1. Luo W, Phung D, Tran T, Gupta S, Rana S, Karmakar C, et al. Guidelines for Developing and Reporting Machine Learning Predictive Models in Biomedical Research: A Multidisciplinary View. J Med Internet Res. 2016 Dec 16;18(12):e323. doi:10.2196/jmir.5870

2. Floridi L, Cowls J, Beltrametti M, Chatila R, Chazerand P, Dignum V, et al. AI4People—An Ethical Framework for a Good AI Society: Opportunities, Risks, Principles, and Recommendations. Minds & Machines. 2018 Dec;28(4):689–707. doi:10.1007/s11023-018-9482-5

3. Reps JM, Schuemie MJ, Suchard MA, Ryan PB, Rijnbeek PR. Design and implementation of a standardized framework to generate and evaluate patient-level prediction models using observational healthcare data. J Am Med Inform Assoc. 2018 Aug 1;25(8):969–75. doi:10.1093/jamia/ocy032 PubMed PMID: 29718407; PubMed Central PMCID: PMC6077830.

4. Cruz Rivera S, Liu X, Chan AW, Denniston AK, Calvert MJ. Guidelines for clinical trial protocols for interventions involving artificial intelligence: the SPIRIT-AI extension. Lancet Digit Health. 2020 Oct;2(10):e549–60. doi:10.1016/S2589-7500(20)30219-3 PubMed PMID: 33328049; PubMed Central PMCID: PMC8212701.

5. Hernandez-Boussard T, Bozkurt S, Ioannidis JPA, Shah NH. MINIMAR (MINimum Information for Medical AI Reporting): Developing reporting standards for artificial intelligence in health care. Journal of the American Medical Informatics Association. 2020 Dec 9;27(12):2011–5. doi:10.1093/jamia/ocaa088

6. Liu X, Cruz Rivera S, Moher D, Calvert MJ, Denniston AK. Reporting guidelines for clinical trial reports for interventions involving artificial intelligence: the CONSORT-AI extension. Lancet Digit Health. 2020 Oct;2(10):e537–48. doi:10.1016/S2589-7500(20)30218-1 PubMed PMID: 33328048; PubMed Central PMCID: PMC8183333.

7. Mongan J, Moy L, Kahn CE. Checklist for Artificial Intelligence in Medical Imaging (CLAIM): A Guide for Authors and Reviewers. Radiol Artif Intell. 2020 Mar;2(2):e200029. doi:10.1148/ryai.2020200029 PubMed PMID: 33937821; PubMed Central PMCID: PMC8017414.

8. Norgeot B, Quer G, Beaulieu-Jones BK, Torkamani A, Dias R, Gianfrancesco M, et al. Minimum information about clinical artificial intelligence modeling: the MI-CLAIM checklist. Nat Med. 2020 Sep;26(9):1320–4. doi:10.1038/s41591-020-1041-y

9. Sengupta PP, Shrestha S, Berthon B, Messas E, Donal E, Tison GH, et al. Proposed Requirements for Cardiovascular Imaging-Related Machine Learning Evaluation (PRIME): A Checklist. JACC: Cardiovascular Imaging. 2020 Sep;13(9):2017–35. doi:10.1016/j.jcmg.2020.07.015

10. Stevens LM, Mortazavi BJ, Deo RC, Curtis L, Kao DP. Recommendations for Reporting Machine Learning Analyses in Clinical Research. Circ: Cardiovascular Quality and Outcomes. 2020 Oct;13(10). doi:10.1161/CIRCOUTCOMES.120.006556

11. Young AT, Xiong M, Pfau J, Keiser MJ, Wei ML. Artificial Intelligence in Dermatology: A Primer. J Invest Dermatol. 2020 Aug;140(8):1504–12. doi:10.1016/j.jid.2020.02.026 PubMed PMID: 32229141.

12. Cabitza F, Campagner A. The need to separate the wheat from the chaff in medical informatics. International Journal of Medical Informatics. 2021 Sep;153:104510. doi:10.1016/j.ijmedinf.2021.104510

13. Ji M, Genchev GZ, Huang H, Xu T, Lu H, Yu G. Evaluation Framework for Successful Artificial Intelligence-Enabled Clinical Decision Support Systems: Mixed Methods Study. J Med Internet Res. 2021 Jun 2;23(6):e25929. doi:10.2196/25929 PubMed PMID: 34076581; PubMed Central PMCID: PMC8209524.

14. Olczak J, Pavlopoulos J, Prijs J, Ijpma FFA, Doornberg JN, Lundström C, et al. Presenting artificial intelligence, deep learning, and machine learning studies to clinicians and healthcare stakeholders: an introductory reference with a guideline and a Clinical AI Research (CAIR) checklist proposal. Acta Orthopaedica. 2021 Sep 3;92(5):513–25. doi:10.1080/17453674.2021.1918389

15. Schwendicke F, Singh T, Lee JH, Gaudin R, Chaurasia A, Wiegand T, et al. Artificial intelligence in dental research: Checklist for authors, reviewers, readers. Journal of Dentistry. 2021 Apr;107:103610. doi:10.1016/j.jdent.2021.103610

16. Bazoukis G, Hall J, Loscalzo J, Antman EM, Fuster V, Armoundas AA. The inclusion of augmented intelligence in medicine: A framework for successful implementation. Cell Rep Med. 2022 Jan 18;3(1):100485. doi:10.1016/j.xcrm.2021.100485 PubMed PMID: 35106506; PubMed Central PMCID: PMC8784713.

17. Daneshjou R, Barata C, Betz-Stablein B, Celebi ME, Codella N, Combalia M, et al. Checklist for Evaluation of Image-Based Artificial Intelligence Reports in Dermatology: CLEAR Derm Consensus Guidelines From the International Skin Imaging Collaboration Artificial Intelligence Working Group. JAMA Dermatol. 2022 Jan 1;158(1):90. doi:10.1001/jamadermatol.2021.4915

18. Fusar-Poli P, Manchia M, Koutsouleris N, Leslie D, Woopen C, Calkins ME, et al. Ethical considerations for precision psychiatry: A roadmap for research and clinical practice. Eur Neuropsychopharmacol. 2022 Oct;63:17–34. doi:10.1016/j.euroneuro.2022.08.001 PubMed PMID: 36041245.

19. Kwong JCC, McLoughlin LC, Haider M, Goldenberg MG, Erdman L, Rickard M, et al. Standardized Reporting of Machine Learning Applications in Urology: The STREAM-URO Framework. European Urology Focus. 2021 Jul;7(4):672–82. doi:10.1016/j.euf.2021.07.004

20. Lu JH, Callahan A, Patel BS, Morse KE, Dash D, Pfeffer MA, et al. Assessment of Adherence to Reporting Guidelines by Commonly Used Clinical Prediction Models From a Single Vendor: A Systematic Review. JAMA Netw Open. 2022 Aug 1;5(8):e2227779. doi:10.1001/jamanetworkopen.2022.27779 PubMed PMID: 35984654; PubMed Central PMCID: PMC9391954.

21. Shen FX, Silverman BC, Monette P, Kimble S, Rauch SL, Baker JT. An Ethics Checklist for Digital Health Research in Psychiatry: Viewpoint. J Med Internet Res. 2022 Feb 9;24(2):e31146. doi:10.2196/31146

22. Vasey B, Nagendran M, Campbell B, Clifton DA, Collins GS, Denaxas S, et al. Reporting guideline for the early stage clinical evaluation of decision support systems driven by artificial intelligence: DECIDE-AI. BMJ. 2022 May 18;377:e070904. doi:10.1136/bmj-2022-070904

23. Abdulazeem H, Whitelaw S, Schauberger G, Klug SJ. A systematic review of clinical health conditions predicted by machine learning diagnostic and prognostic models trained or validated using real-world primary health care data. PLoS One. 2023 Sep;18(9). Located at: ProQuest Central; 2862748910. doi:10.1371/journal.pone.0274276

24. Cacciamani GE, Chu TN, Sanford DI, Abreu A, Duddalwar V, Oberai A, et al. PRISMA AI reporting guidelines for systematic reviews and meta-analyses on AI in healthcare. Nat Med. 2023 Jan;29(1):14–5. doi:10.1038/s41591-022-02139-w

25. Debray TPA, Collins GS, Riley RD, Snell KIE, Van Calster B, Reitsma JB, et al. Transparent reporting of multivariable prediction models developed or validated using clustered data (TRIPOD-Cluster): explanation and elaboration. BMJ. 2023 Feb 7;380:e071058. doi:10.1136/bmj-2022-071058

26. Elvidge J, Hawksworth C, Avşar TS, Zemplenyi A, Chalkidou A, Petrou S, et al. Consolidated Health Economic Evaluation Reporting Standards for Interventions That Use Artificial Intelligence (CHEERS-AI). Value in Health. 2024 Sep;27(9):1196–205. doi:10.1016/j.jval.2024.05.006

27. Klement W, El Emam K. Consolidated Reporting Guidelines for Prognostic and Diagnostic Machine Learning Modeling Studies: Development and Validation. J Med Internet Res. 2023 Aug 31;25:e48763. doi:10.2196/48763

28. Kwong JCC, Khondker A, Lajkosz K, McDermott MBA, Frigola XB, McCradden MD, et al. APPRAISE-AI Tool for Quantitative Evaluation of AI Studies for Clinical Decision Support. JAMA Netw Open. 2023 Sep 25;6(9):e2335377. doi:10.1001/jamanetworkopen.2023.35377

29. Murphy TI, Armitage JA, van Wijngaarden P, Abel LA, Douglass AG. A guide to optometrists for appraising and using artificial intelligence in clinical practice. Clin Exp Optom. 2023 Aug;106(6):569–79. doi:10.1080/08164622.2023.2197578 PubMed PMID: 37078176.

30. Collins GS, Moons KGM, Dhiman P, Riley RD, Beam AL, Van Calster B, et al. TRIPOD+AI statement: updated guidance for reporting clinical prediction models that use regression or machine learning methods. BMJ. 2024 Apr 16;385:e078378. doi:10.1136/bmj-2023-078378

31. Elfer K, Gardecki E, Garcia V, Ly A, Hytopoulos E, Wen S, et al. Reproducible Reporting of the Collection and Evaluation of Annotations for Artificial Intelligence Models. Modern Pathology. 2024 Apr;37(4):100439. doi:10.1016/j.modpat.2024.100439

32. El Emam K, Leung TI, Malin B, Klement W, Eysenbach G. Consolidated Reporting Guidelines for Prognostic and Diagnostic Machine Learning Models (CREMLS). J Med Internet Res. 2024 May 2;26:e52508. doi:10.2196/52508

33. Guni A, Sounderajah V, Whiting P, Bossuyt P, Darzi A, Ashrafian H. Revised Tool for the Quality Assessment of Diagnostic Accuracy Studies Using AI (QUADAS-AI): Protocol for a Qualitative Study. JMIR Res Protoc. 2024 Sep 18;13:e58202. doi:10.2196/58202

34. Kapoor S, Cantrell EM, Peng K, Pham TH, Bail CA, Gundersen OE, et al. REFORMS: Consensus-based Recommendations for Machine-learning-based Science. Sci Adv. 2024 May 3;10(18):eadk3452. doi:10.1126/sciadv.adk3452

35. Labkoff S, Oladimeji B, Kannry J, Solomonides A, Leftwich R, Koski E, et al. Toward a responsible future: recommendations for AI-enabled clinical decision support. J Am Med Inform Assoc. 2024 Nov 1;31(11):2730–9. doi:10.1093/jamia/ocae209 PubMed PMID: 39325508; PubMed Central PMCID: PMC11491642.

36. Masters K, Salcedo D. A checklist for reporting, reading and evaluating Artificial Intelligence Technology Enhanced Learning (AITEL) research in medical education. Medical Teacher. 2024 Sep;46(9):1175–9. doi:10.1080/0142159X.2023.2298756

37. Ning Y, Teixayavong S, Shang Y, Savulescu J, Nagaraj V, Miao D, et al. Generative artificial intelligence and ethical considerations in health care: a scoping review and ethics checklist. Lancet Digit Health. 2024 Nov;6(11):e848–56. doi:10.1016/S2589-7500(24)00143-2 PubMed PMID: 39294061; PubMed Central PMCID: PMC11542614.

38. Ray A., Sarkar S., Schwenker F., Sarkar R. Decoding skin cancer classification: perspectives, insights, and advances through researchers’ lens. Sci Rep. 2024;14(1):30542. Located at: Ovid Emcare <2021 to 2025 Week 28>. doi:10.1038/s41598-024-81961-3

39. Tejani AS, Klontzas ME, Gatti AA, Mongan JT, Moy L, Park SH, et al. Checklist for Artificial Intelligence in Medical Imaging (CLAIM): 2024 Update. Radiology: Artificial Intelligence. 2024 Jul 1;6(4):e240300. doi:10.1148/ryai.240300

40. Uribe SE, Maldupa I, Schwendicke F. Integrating Generative AI in Dental Education: A Scoping Review of Current Practices and Recommendations. Eur J Dent Educ. 2025 May;29(2):341–55. doi:10.1111/eje.13074 PubMed PMID: 39891376; PubMed Central PMCID: PMC12006694.

41. Warren BE, Bilbily A, Gichoya JW, Chartier LB, Fawzy A, Barragán C, et al. An Introductory Guide to Artificial Intelligence in Interventional Radiology: Part 2: Implementation Considerations and Harms. Can Assoc Radiol J. 2024 Aug;75(3):568–74. doi:10.1177/08465371241236377 PubMed PMID: 38445517.

42. Kalaycıoğlu O, Pavlou M, Akhanlı SE, De Belder MA, Ambler G, Omar RZ. Evaluating the sample size requirements of tree-based ensemble machine learning techniques for clinical risk prediction. Stat Methods Med Res. 2025 Jul;34(7):1356–72. doi:10.1177/09622802251338983

43. Pan American Health Organization (PAHO). AI prompt design for public health: Using generative AI responsibly - OPS/OMS | Organización Panamericana de la Salud [Internet]. Pan American Health Organization,. Washington: Pan American Health Organization,; 2025 [cited 2025 Nov 28]. (PAHO/EIH/IS/25-0008). Available from: https://www.paho.org/es/documentos/ai-prompt-design-public-health-using-generative-ai-responsibly

44. Sounderajah V, Guni A, Liu X, Collins GS, Karthikesalingam A, Markar SR, et al. The STARD-AI reporting guideline for diagnostic accuracy studies using artificial intelligence. Nat Med. 2025 Oct;31(10):3283–9. doi:10.1038/s41591-025-03953-8

45. Tuygunov N, Samaranayake L, Khurshid Z, Rewthamrongsris P, Schwendicke F, Osathanon T, et al. The Transformative Role of Artificial Intelligence in Dentistry: A Comprehensive Overview Part 2: The Promise and Perils, and the International Dental Federation Communique. Int Dent J. 2025 Apr;75(2):397–404. doi:10.1016/j.identj.2025.02.006 PubMed PMID: 40011130; PubMed Central PMCID: PMC11976557.

46. Wang Y, Cheungpasitporn W, Ali H, Qing J, Thongprayoon C, Kaewput W, et al. A practical guide for nephrologist peer reviewers: evaluating artificial intelligence and machine learning research in nephrology. Ren Fail. 2025 Dec;47(1):2513002. doi:10.1080/0886022X.2025.2513002 PubMed PMID: 40620096; PubMed Central PMCID: PMC12239107.
