# Supplementary material for: Evaluation Frameworks for Clinical AI Incorporating Validation Strategies, Real-World Applicability, and Ethical Principles: Scoping Review
Source: J Med Internet Res. 2026 Jul 22;28:e78168. doi: 10.2196/78168 (PMC13392654; doi:10.2196/78168)
Supplement: Multimedia Appendix 2 [file jmir-v28-e78168-s002.docx]

**Table 2.** Excluded documents.

| **Author, Year (Ref.)** | **Title of Excluded Article** | | **Number** |
| --- | --- | --- | --- |
| **Methodological or development protocols without validation results or empirical testing (N = 18)** | | | |
| Andaur N., 2020 (1) | | Protocol for a systematic review on the methodological and reporting quality of prediction model studies using machine learning techniques. | 1 |
| Nagendrababu V., 2021 (2) | | Preferred Reporting Items for Diagnostic Accuracy Studies in Endodontics (PRIDASE) guidelines: a development protocol | 2 |
| Liu N., 2022 (3) | | Leveraging Large-Scale Electronic Health Records and Interpretable Machine Learning for Clinical Decision Making at the Emergency Department: Protocol for System Development and Validation. | 3 |
| Satchwell L., 2022 (4) | | Development of machine learning support for reading whole body diffusion-weighted MRI (WB-MRI) in myeloma for the detection and quantification of the extent of disease before and after treatment (MALIMAR): protocol for a cross-sectional diagnostic test accuracy study. | 4 |
| Liu V., 2023 (5) | | User-Initiated Symptom Assessment With an Electronic Symptom Checker: Protocol for a Mixed Methods Validation Study. | 5 |
| Fu H., 2024 (6) | | AI assisted reader evaluation in acute CT head interpretation (AI-REACT): protocol for a multireader multicase study | 6 |
| Hou Z., 2024 (7) | | Development, validation and economic evaluation of a machine learning algorithm for predicting the probability of kidney damage in patients with hyperuricaemia: protocol for a retrospective study | 7 |
| Howell F., 2024 (8) | | AI assisted reader evaluation in acute CT head interpretation (AI-REACT): protocol for a multireader multicase study. | 8 |
| Sguanci M., 2024 (9) | | Protocol for conducting a systematic review on diagnostic accuracy in clinical research. | 9 |
| Chitrapady S., 2025 (10) | | Machine-learning-based artificial intelligence tools for the diagnosis of tropical fevers: a systematic review and meta-analysis protocol of diagnostic test accuracy. | 10 |
| Olwendo K., 2025 (11) | | Development and validation of a predictive model for new HIV infection screening among persons 15 years and above in primary healthcare settings in Kenya: a study protocol. | 11 |
| Lazaridou A., 2025 (12) | | Predictive modelling of clinically significant depressive symptoms after coronary artery bypass graft surgery: protocol for a multicentre observational study in two Swiss hospitals (the PsyCor study). | 12 |
| Pereira O., 2025 (13) | | Protocol for evaluation of a virtual wheelchair simulator in assessing mobility skills and cognitive abilities in diverse populations: A multicentric mixed-methods pilot study. | 13 |
| Tonde B., 2025 (14) | | Predictive modelling methods of hospital readmission risks for patients with chronic obstructive pulmonary disease (COPD): a systematic review protocol | 14 |
| Wadia H., 2025 (15) | | Effectiveness of predictive scoring systems in predicting mortality in relation to baseline kidney function in adult intensive care unit patients: a systematic review protocol. | 15 |
| Alpern E., 2025 (16) | | Derivation and Validation of Predictive Models for Early Pediatric Sepsis | 16 |
| Gupta A., 2025 (17) | | Applications of artificial intelligence in abdominal imaging | 17 |
| Palermi S., 2025 (18) | | Artificial intelligence and the electrocardiogram: A modern renaissance | 18 |
| Hu Y., 2025 (19) | | Large language models in nephrology: applications and challenges in chronic kidney disease management | 19 |
| **Introductory series or conceptual papers without real-world application or empirical evaluation (N= 64)** | | | |
| Altman D.,  2005 (20) | Developing guidelines for reporting healthcare research: scientific rationale and procedures. | | 1 |
| Cleophas T,  2008 (21) | Validating diagnostic tests, correct and incorrect methods, new developments. | | 2 |
| Manchikanti L.,  2009 (22) | Evidence-based medicine, systematic reviews, and guidelines in interventional pain management: part 5. Diagnostic accuracy studies. | | 3 |
| Vandenbroucke J.,  2009 (23) | STREGA, STROBE, STARD, SQUIRE, MOOSE, PRISMA, GNOSIS, TREND, ORION, COREQ, QUOROM, REMARK... and CONSORT: for whom does the guideline toll? | | 4 |
| Simera I.,  2013 (24) | Get the content right: following reporting guidelines will make your research paper more complete, transparent and usable. | | 5 |
| Glasziou P.,  2014 (25) | Reducing waste from incomplete or unusable reports of biomedical research. | | 6 |
| Dinga R.,  2019 (26) | Beyond accuracy: Measures for assessing machine learning models, pitfalls and guidelines | | 7 |
| Grech V.,  2019 (27) | Write a Scientific Paper (WASP): Guidelines for reporting medical research. | | 8 |
| Itani S.,  2019 (28) | Specifics of medical data mining for diagnosis aid: A survey. Expert Systems with Applications. | | 9 |
| De Rooij, Mark.,  2020 (29) | Cross-Validation: A Method Every Psychologist Should Know. | | 10 |
| Morgenstern J.,  2020 (30) | Predicting population health with machine learning: a scoping review. BMJ Open | | 11 |
| Schwendicke F., 2020 (31) | Artificial {Intelligence} in {Dentistry}: {Chances} and {Challenges}. | | 12 |
| Young A., 2020 (32) | Artificial {Intelligence} in {Dermatology}: {A} {Primer}. | | 13 |
| Clausen C.,  2021 (33) | Clinical Decision Support Systems: An Innovative Approach to Enhancing Child and Adolescent Mental Health Services. | | 14 |
| Clement J., 2021 (34) | Augmenting the {Transplant} {Team} {With} {Artificial} {Intelligence}: {Toward} {Meaningful} {AI} {Use} in {Solid} {Organ} {Transplant}. | | 15 |
| Sitch A.,  2021 (35) | Introduction to diagnostic test accuracy studies. | | 16 |
| Bazoukis G., 2022 (36) | The inclusion of augmented intelligence in medicine: {A} framework for successful implementation. | | 17 |
| Cassinelli P., 2022 (37) | Machine {Learning} in {Differentiating} {Gliomas} from {Primary} {CNS} {Lymphomas}: {A} {Systematic} {Review}, {Reporting} {Quality}, and {Risk} of {Bias} {Assessment}. | | 18 |
| Collins J., 2022 (38) | Ethical implications of {AI} in robotic surgical training: {A} {Delphi} consensus statement. | | 19 |
| Crossnohere N., 2022 (39) | Guidelines for {Artificial} {Intelligence} in {Medicine}: {Literature} {Review} and {Content} {Analysis} of {Frameworks}. | | 20 |
| Fanciullo C.,  2022 (40) | Radiomics of Musculoskeletal Sarcomas: A Narrative Review. | | 21 |
| Khoury P.,  2022 (41) | A Framework for Augmented Intelligence in Allergy and Immunology Practice and Research-A Work Group Report of the AAAAI Health Informatics, Technology, and Education Committee. | | 22 |
| Lu J.,  2022 (42) | Considerations in the Reliability and Fairness Audits of Predictive Models for Advance Care Planning | | 23 |
| Misiak M.,  2022 (43) | Checklists for reporting research in Advances in Clinical and Experimental Medicine: How to choose a proper one for your manuscript? Advances in Clinical and Experimental Medicine | | 24 |
| Kumbhar U.,  2023 (44) | Explainable AI-Powered IoT Systems for Predictive and Preventive Healthcare - A Framework for Personalized Health Management and Wellness Optimization. | | 25 |
| Van Velzen M., 2023 (45) | 21st century (clinical) decision support in nursing and allied healthcare. {Developing} a learning health system: a reasoned design of a theoretical framework. | | 26 |
| Dirnfeld Ruth.,  2024 (46) | Integrating AI and DTs: challenges and opportunities in railway maintenance application and beyond | | 27 |
| Gatineau G., 2024 (47) | Development and reporting of artificial intelligence in osteoporosis management. | | 28 |
| Grech V.,  2024 (48) | STROBE, CONSORT, PRISMA, MOOSE, STARD, SPIRIT, and other guidelines - Overview and application. | | 29 |
| Hogg H., 2024 (49) | Clinical {Evaluation} of {Artificial} {Intelligence}-{Enabled} {Interventions}. | | 30 |
| Loftus T.,  2024 (50) | Longitudinal clinical decision support for assessing decisions over time: State-of-the-art and future directions. | | 31 |
| Piffer S.,  2024 (51) | Tackling the small data problem in medical image classification with artificial intelligence: a systematic review. | | 32 |
| Straus T., 2024 (52) | Artificial intelligence: a primer for pediatric radiologists | | 33 |
| Valentina T.,  2024 (53) | The development of early warning scores or alerting systems for the prediction of adverse events in psychiatric patients: a scoping review. | | 34 |
| Warren B., 2024 (54) | An Introductory Guide to Artificial Intelligence in Interventional Radiology: Part 1 Foundational Knowledge. | | 35 |
| Alderman J., 2025 (55) | Tackling algorithmic bias and promoting transparency in health datasets: the {STANDING} {Together} consensus recommendations. | | 36 |
| Bansal R.,  2025 (56) | Role of Artificial Intelligence and Machine Learning in Conservative Dentistry and Endodontics: A Review. Cureus | | 37 |
| Carlson D., 2025 (57) | The {NERVE}-{ML} (neural engineering reproducibility and validity essentials for machine learning) checklist: ensuring machine learning) checklist: ensuring machine learning advances neural engineering | | 38 |
| Coen H., 2025 (58) | Assessment of bias in scoring of {AI}-based radiotherapy segmentation and planning studies using modified {TRIPOD} and {PROBAST} guidelines as an example | | 39 |
| Critelli B., 2025 (59) | A systematic review of machine learning-based prognostic models for acute pancreatitis: {Towards} improving methods and reporting quality. | | 40 |
| Choi J., 2025 (60) | Artificial intelligence in surgery research: {Successfully} implementing {AI} clinical decision support models. | | 41 |
| Dogeun P.,  2025 (61) | Early warning score and feasible complementary approach using artificial intelligence-based bio-signal monitoring system: a review. | | 42 |
| Du H.,  2025 (62) | Big Data–Driven Health Portraits for Personalized Management in Noncommunicable Diseases: Scoping Review. | | 43 |
| El Arab R.,  2025 (63) | Integrating Artificial Intelligence into Perinatal Care Pathways: A Scoping Review of Reviews of Applications, Outcomes, and Equity. | | 44 |
| Gallifant J., 2025 (64) | The {TRIPOD}-{LLM} reporting guideline for studies using large language models | | 45 |
| Garcia Ch., 2025 (65) | Establishing a comprehensive artificial intelligence lifecycle framework for laboratory medicine and pathology: {A} series introduction. | | 46 |
| Kellerhuis B.,  2025 (66) | Expert panel as reference standard procedure in diagnostic accuracy studies: a systematic scoping review and methodological guidance. | | 47 |
| Kim D.,  2025 (67) | Personalized Medical Approach in Gastrointestinal Surgical Oncology: Current Trends and Future Perspectives. | | 48 |
| Kumar R.,  2025 (68) | Emerging Diagnostic Approaches for Musculoskeletal Disorders: Advances in Imaging, Biomarkers, and Clinical Assessment. | | 49 |
| Mann J.,  2025 (69) | Machine learning or traditional statistical methods for predictive modelling in perioperative medicine: A narrative review. | | 50 |
| Nivethitha V.,  2025 (70) | Empowering public health: Leveraging AI for early detection, treatment, and disease prevention in communities - A scoping review. | | 51 |
| Obeagu EI.,  2025 (71) | Big data analytics and machine learning in hematology: Transformative insights, applications and challenges. | | 52 |
| Radulescu R., 2025 (72) | The {Role} of {Artificial} {Intelligence} in {Personalized} {Medicine}: {A} {Computer} {Science} {Perspective} | | 53 |
| Ramwala OA.,  2025 (73) | A framework for developing Cloud-based infrastructures for the External Clinical Validation of AI in Medical Imaging. | | 54 |
| Samaranayake L., 2025 (74) | The Transformative Role of Artificial Intelligence in Dentistry: A Comprehensive Overview. Part 1: Fundamentals of AI, and its Contemporary Applications in Dentistry | | 55 |
| Vasileios L.,  2025 (75) | Artificial Intelligence in Thoracic Surgery: A Review Bridging Innovation and Clinical Practice for the Next Generation of Surgical Care. | | 56 |
| Vallée A.,  2025 (76) | Digital Twins for Personalized Medicine Require Epidemiological Data and Mathematical Modeling: Viewpoint. | | 57 |
| Abbara S., 2025 (77) | Artificial intelligence and infectious diseases: Scope and perspectives | | 58 |
| Opel N., 2025 (78) | Transforming mental health research and care through artificial intelligence | | 59 |
| Wiest I., 2025 (79) | Large language models for clinical decision support in gastroenterology and hepatology | | 60 |
| Du Y., 2025 (80) | Artificial intelligence in chronic disease self-management: current applications and future directions | | 61 |
| Sakamoto A., 2025 (81) | Artificial intelligence in echocardiography: current applications and future perspectives | | 62 |
| Maznyczka A., 2025 (82) | Artificial Intelligence in Valvular Heart Disease | | 63 |
| Gong E., 2025 (83) | Role of artificial intelligence in gastric diseases | | 64 |
| **Comparative or descriptive analyses of existing AI checklists (N= 48)** | | | |
| Bossuyt P.,  2003 (84) | Towards complete and accurate reporting of studies of diagnostic accuracy: the STARD initiative. Standards for Reporting of Diagnostic Accuracy. | | 1 |
| Bossuyt P.,  2003 (85) | Towards complete and accurate reporting of studies of diagnostic accuracy: the STARD initiative. | | 2 |
| Bossuyt P.,  2003 (86) | The STARD statement for reporting studies of diagnostic accuracy: explanation and elaboration. | | 3 |
| Ell P.,  2003 (87) | STARD and CONSORT: time for reflection. | | 4 |
| Bossuyt P.,  2004 (88) | Towards complete and accurate reporting of studies of diagnostic accuracy: the STARD initiative. | | 5 |
| Altman, D.,  2005 (89) | [Diagnostic (STARD) and prognostic (REMARK) studies]. | | 6 |
| Cobo E.,  2007 (90) | Statistical reviewers improve reporting in biomedical articles: a randomized trial. | | 7 |
| Mundi R.,  2008 (91) | Checklists to improve the quality of the orthopaedic literature. | | 8 |
| Verbeek J.,  2008 (92) | Moose Consort Strobe and Miame Stard Remark or how can we improve the quality of reporting studies. | | 9 |
| Meerpohl J.,  2009 (93) | Reporting guidelines are also useful for readers of medical research publications: CONSORT, STARD, STROBE and others. | | 10 |
| Areia M.,  2010 (94) | Quality reporting of endoscopic diagnostic studies in gastrointestinal journals: where do we stand on the use of the STARD and CONSORT statements? | | 11 |
| Perneger T.,  2010 (95) | Citation analysis of identical consensus statements revealed journal-related bias | | 12 |
| Costa L.,  2011 (96) | Transparent reporting of studies relevant to physical therapy practice. | | 13 |
| Meerpohl J.,  2011 (97) | Are pediatric Open Access journals promoting good publication practice? An analysis of author instructions. | | 14 |
| Selman T. 2011 (98) | The quality of reporting of primary test accuracy studies in obstetrics and gynaecology: application of the STARD criteria | | 15 |
| Hirst A.,  2012 (99) | Are peer reviewers encouraged to use reporting guidelines? A survey of 116 health research journals. | | 16 |
| Stevens A.,  2014 (100) | Relation of completeness of reporting of health research to journals’ endorsement of reporting guidelines: systematic review. | | 17 |
| Bossuyt P.,  2015 (101) | STARD 2015: An Updated List of Essential Items for Reporting Diagnostic Accuracy Studies. | | 18 |
| Moreno-Ramírez, D.,  2015 (102) | CONSORT, STROBE, and STARD. Tools to improve the reporting of research. | | 19 |
| Cohen J.,  2016 (103) | STARD 2015 guidelines for reporting diagnostic accuracy studies: explanation and elaboration. | | 20 |
| Johansen M.,  2016 (104) | Guidelines for Reporting Medical Research: A Critical Appraisal. International Scholarly Research Notices, | | 21 |
| Korevaar D.,  2016 (105) | Updating standards for reporting diagnostic accuracy: the development of STARD 2015. | | 22 |
| Sims M., 2016 (106) | Do emergency medicine journals promote trial registration and adherence to reporting guidelines? A survey of "Instructions for Authors". | | 23 |
| McGrath T.,  2017 (107) | Recommendations for reporting of systematic reviews and meta-analyses of diagnostic test accuracy: a systematic review. | | 24 |
| Toews I.,  2017 (108) | Guidance in author instructions of hematology and oncology journals: A cross sectional and longitudinal study. | | 25 |
| Wayant C.,  2017 (109) | Hematology journals do not sufficiently adhere to reporting guidelines: a systematic review. Journal of Thrombosis and Haemostasis | | 26 |
| Hong P.,  2018 (110) | Reporting of imaging diagnostic accuracy studies with focus on MRI subgroup: Adherence to STARD 2015. | | 27 |
| Jin Y.  2018 (111) | Does the medical literature remain inadequately described despite having reporting guidelines for 21 years? - A systematic review of reviews: an update. | | 28 |
| Mackinnon S.,  2018 (112) | Are methodological quality and completeness of reporting associated with citation-based measures of publication impact? A secondary analysis of a systematic review of dementia biomarker studies. | | 29 |
| Cooper C.,  2020 (113) | An Evaluation of Reporting Guidelines and Clinical Trial Registry Requirements Among Addiction Medicine Journals. | | 30 |
| Hogan K.,  2020 (114) | Compliance With Standards for STARD 2015 Reporting Recommendations in Pathology. | | 31 |
| Prager R.,  2020 (115) | Adherence to the Standards for Reporting of Diagnostic Accuracy (STARD) 2015 Guidelines in Acute Point-of-Care Ultrasound Research. | | 32 |
| Andaur Navarro CL.,  2022 (116) | Completeness of reporting of clinical prediction models developed using supervised machine learning: a systematic review. | | 33 |
| Kazi S.,  2022 (117) | Evaluating the Impact of Peer Review on the Completeness of Reporting in Imaging Diagnostic Test Accuracy Research. | | 34 |
| Lu JH.,  2022 (118) | Assessment of Adherence to Reporting Guidelines by Commonly Used Clinical Prediction Models From a Single Vendor: A Systematic Review. | | 35 |
| Zhou J.,  2022 (119) | The relationship between endorsing reporting guidelines or trial registration and the impact factor or total citations in surgical journals. | | 36 |
| Andaur Navarro CL.,  2023 (120) | Systematic review finds "spin" practices and poor reporting standards in studies on machine learning-based prediction models. | | 37 |
| Gorrepati P.,  2023 (121) | Evaluating dermatology journals’ use of reporting guidelines in “Author Guidelines.” | | 38 |
| Ozdag Y.,  2023 (122) | Adherence to Complication Reporting for Randomized Controlled Trials Contained in Clinical Practice Guidelines for the Management of Carpal Tunnel Syndrome. | | 39 |
| Zhong J.,  2023 (123) | The endorsement of general and artificial intelligence reporting guidelines in radiological journals: a meta-research study. | | 40 |
| Kashif Al-Ghita M.,  2025 (124) | Evaluation of Imaging Research Adherence to the STARD 2015 Reporting Guideline: Update 9 Years After Implementation and Baseline Assessment. | | 41 |
| Khalid S.,  2025 (125) | An Appraisal of the Quality of Development and Reporting of Predictive Models in Spine Surgery. | | 42 |
| Knez N.,  2023 (126) | Publications on the diagnostic accuracy of dermatopathology tests: A cross-sectional quality analysis. | | 43 |
| Kocak B.,  2024 (127) | Self-reported checklists and quality scoring tools in radiomics: a meta-research. | | 44 |
| Uribe S.,  2025 (128) | Evaluating dental AI research papers: Key considerations for editors and reviewers. | | 45 |
| Ko J., 2025 (129) | Adherence of {Studies} on {Large} {Language} {Models} for {Medical} {Applications} {Published} in {Leading} {Medical} {Journals} {According} to the {MI}-{CLEAR}-{LLM} {Checklist}. | | 46 |
| Koçak B., 2025 (130) | Adherence to the Checklist for Artificial Intelligence in Medical Imaging (CLAIM): an umbrella review with a comprehensive two-level analysis. | | 47 |
| Yang S., 2025 (131) | Machine learning-driven risk stratification for distant metastasis in gastric cancer: A comparative study of clinical features and composite indices integrated models | | 48 |
| **Out-of-scope domains, including and expert conference proceedings (N=89)** | | | |
| 1996 (132) | Animal Welfare and Ethics | | 1 |
| 1997 (133) | XVIII th International Symposium on Cerebral Blood Flow and Metabolism | | 2 |
| 1997 (134) | British Society for Dental Research The British Division of the IADR 45th Annual Meeting April 7–10, 1997, University of Sussex, Brighton, England | | 3 |
| Gunn S.,  2002 (135) | Structural Modelling with Sparse Kernels. | | 4 |
| 2005 (136) | Oral Presentations | | 5 |
| Hall S.,  2008 (137) | A review of the literature in applied and specialised kinesiology. | | 6 |
| 2010 (138) | The 9th International Congress on SLE June 24 – 27 2010, Vancouver, Canada | | 7 |
| 2010 (139) | Oral Presentations | | 8 |
| 2012 (140) | Poster I | | 9 |
| 2013 (141) | The 10th International Congress on SLE April 18–21 2013, Buenos Aires, Argentina Oral Presentations” | | 10 |
| 2013 (142) | UEG Week 2013 Poster Presentations | | 11 |
| 2013 (143) | ECTRIMS 2013: Posters I | | 12 |
| Chapman R., 2014 (144) | Ovary transcriptome profiling via artificial intelligence reveals a transcriptomic fingerprint predicting egg quality in striped bass, {Morone} saxatilis | | 13 |
| 2014 (145) | UEG Week 2014 Poster Presentations | | 14 |
| 2015 (146) | Poster Session 1. | | 15 |
| 2015 (147) | UEG Week 2015 Oral Presentations | | 16 |
| 2015 (148) | UEG Week 2015 Poster Presentations | | 17 |
| 2016 (149) | UEG Week 2016 Poster Presentations | | 18 |
| 2016 (150) | ePosters | | 19 |
| 2016 (151) | Poster Session 1 | | 20 |
| 2016 (152) | Poster Session 2. | | 21 |
| 2016 (153) | Oral Presentations | | 22 |
| 2017 (154) | Poster Session 1 | | 23 |
| 2017 (155) | UEG Week 2017 Poster Presentations | | 24 |
| 2017 (156) | ePosters | | 25 |
| 2017 (157) | Poster Session 2 | | 26 |
| 2017 (158) | UEG Week 2017 Oral Presentations | | 27 |
| 2017 (159) | E-Posters | | 28 |
| Hayashi Y.,  2018 (160) | High Accuracy-priority Rule Extraction for Reconciling Accuracy and Interpretability in Credit Scoring. | | 29 |
| Evans Barbara.,  2018 (161) | The Challenge of Regulating Clinical Decision Support Software After 21st Century Cures | | 30 |
| 2018 (162) | ePosters | | 31 |
| 2018 (163) | Oral presentations | | 32 |
| 2018 (164) | Poster Session 1 | | 33 |
| 2018 (165) | Poster Session 2 | | 34 |
| 2018 (166) | UEG Week 2018 Poster Presentations | | 35 |
| 2018 (167) | 2018 Southern Regional Meeting | | 36 |
| Scott J.,  2019 (168) | Reproductive immunology from the perspective of the clinician. | | 37 |
| 2019 (169) | UEG Week 2019 Poster Presentations | | 38 |
| 2019 (170) | ECTRIMS 2019 - Poster Session 2 | | 39 |
| 2019 (171) | E-Posters | | 40 |
| Bevilacqua  M.,  2020 (172) | Digital Twin Reference Model Development to Prevent Operators’ Risk in Process Plants. | | 41 |
| Pena H. P.,  2020 (173) | Accuracy of diagnostic tests for American tegumentary leishmaniasis: a systematic literature review with meta-analyses. Tropical Medicine & International Health | | 42 |
| 2020 (174) | UEG Week 2020 Poster Presentations | | 43 |
| 2020 (175) | Midwest Clinical and Translational Research Meeting of CSCTR and MWAFMR | | 44 |
| Arora, Sanjay K.,  2021 (176) | Building a Sample Frame of SMEs Using Patent, Search Engine, and Website Data | | 45 |
| Bukhsh Z.,  2021 (177) | Damage detection using in-domain and cross-domain transfer learning. | | 46 |
| Bjoersum-Meyer T.,  2021 (178) | Long-term Functional Urinary and Sexual Outcomes in Patients with Anorectal Malformations-A Systematic Review. | | 47 |
| Halder A.,  2021 (179) | AKT Inhibitors: The Road Ahead to Computational Modeling-Guided Discovery. | | 48 |
| Kochueva O.,  2021 (180) | Data Analysis and Symbolic Regression Models for Predicting CO and NOx Emissions from Gas Turbines. | | 49 |
| Li Yan.,  2021 (181) | Consistency of ranking was evaluated as new measure for prediction model stability: longitudinal cohort study.138, 168-177. | | 50 |
| Obeng-Gyasi E.,  2021 (182) | Lead Distribution in Urban Soil in a Medium-Sized City: Household-Scale Analysis. | | 51 |
| 2021 (183) | ECTRIMS 2021 – ePoster | | 52 |
| Czub N.,  2022 (184) | Do AutoML-Based QSAR Models Fulfill OECD Principles for Regulatory Assessment? A 5-HT1A Receptor Case. | | 53 |
| Pawar Prathmesh Vasant.,  2022 (185) | Big Data Analytics in Logistics and Supply Chain Management: A Review of Literature | | 54 |
| 2022 (186) | ECTRIMS 2022 – Poster | | 55 |
| 2022 (187) | ACTRIMS Forum 2022 – Poster Presentations | | 56 |
| 2022 (188) | ECTRIMS 2022 – Oral Presentations | | 57 |
| Endalie D., 2023 (189) | Deep learning-based idiomatic expression recognition for the {Amharic} language | | 58 |
| Garcia Valencia O.,  2023 (190) | Enhancing Kidney Transplant Care through the Integration of Chatbot. | | 59 |
| Sada Del Real K.., 2023 (191) | Discovering the mechanism of action of drugs with a sparse explainable network | | 60 |
| 2023 (192) | ACTRIMS Forum 2023 – Poster Presentations | | 61 |
| Aldousari Elham.,  2024 (193) | Artificial intelligence and health information: A bibliometric analysis of three decades of research | | 62 |
| Ghatage Swapnil V..2024 (194) | Development of modelling and digitalization tools for alumina refinery. | | 63 |
| Jibrin A.,  2024 (195) | Tracking the impact of heavy metals on human health and ecological environments in complex coastal aquifers using improved machine learning optimization. | | 64 |
| Nguyen V.-N., 2024 (196) | Using a hybrid neural network architecture for {DNA} sequence representation: {A} study on {N4}-methylcytosine sites | | 65 |
| Perroni Marcos Gonçalves.,  2024 (197) | Integrating Relative Efficiency Models with Machine Learning Algorithms for Performance Prediction | | 66 |
| Time Andrei.,  2024 (198) | Integrating technical and business aspects in Software as a Service: Systematic literature review, trends, and research directions | | 67 |
| 2024 (199) | 45th Annual North American Meeting of the Society for Medical Decision Making, Philadelphia, Pennsylvania, United States, 22-25 October 2023” | | 68 |
| 2024 (200) | ECTRIMS 2024 Late Breaking Poster | | 69 |
| 2024 (201) | E-Posters | | 70 |
| 2024 (202) | ECTRIMS 2024 – ePoster | | 71 |
| 2024 (203) | ECTRIMS 2024 – Poster | | 72 |
| 2024 (204) | ECTRIMS 2024 – Oral Presentations | | 73 |
| Alqaraleh M.,  2025 (205) | Exploring the impact of artificial intelligence integration on medication error reduction: A nursing perspective. | | 74 |
| Barreto Sandra.,  2025 (206) | Near-Death Experiences: A Bibliometric and Systematic Review of Published Literature From 1977 to 2025”. | | 75 |
| Bassil KC.,  2025 (207) | Hoop of hype? AI in mental healthcare: hope or hype? | | 76 |
| Hanum U.,  2025 (208) | Validation of new AI-based classification method for in silico cardiac safety assessment of drugs following the CiPA framework. | | 77 |
| Kalaycıoğlu Oya, Menelaos P.,  2025 (209) | Evaluating the sample size requirements of tree-based ensemble machine learning techniques for clinical risk prediction. | | 78 |
| Kun W.,  2025 (210) | Comparative analysis of the performance of the large language models ChatGPT-3.5, ChatGPT-4 and Open AI-o1 in the field of Programmed Cell Death in myeloma | | 79 |
| Löfström T.,  2025 (211) | Calibrated explanations for regression. | | 80 |
| Marín Díaz Gabriel.,  2025 (212) | Quality Management in Chemical Processes Through Fuzzy Analysis: A Fuzzy C-Means and Predictive Models Approach. | | 81 |
| Mohsin SS.,  2025 (213) | A real-time web-based telemedicine framework based on AI and IoMT for emergency triage and initial diagnostics: the TeleMedQuick solution | | 82 |
| Miedema J.,  2025 (214) | Balancing ethics and statistics: machine learning facilitates highly accurate classification of mice according to their trait anxiety with reduced sample sizes. | | 83 |
| Millán-Palacios S.,  2025 (215) | Investment Portfolios Optimization with Genetic Algorithm: An Approach Applied to the Spanish Market (IBEX 35). | | 84 |
| Martorell-Marugan J., 2025 (216) | Explainable deep neural networks for predicting sample phenotypes from single-cell transcriptomics | | 85 |
| Nikoloudi Maria.,  2025 (217) | Artificial Intelligence in Palliative Care: A Scoping Review of Current Applications, Challenges, and Future Directions | | 86 |
| 2025 (218) | Retraction Notice | | 87 |
| 2025 (219) | ESAO 2025 Innovations in (bio)artificial organs and organ models | | 88 |
| Vora S., 2025 (220) | Preface to the 10th Biennial COAST Conference: AI‐ and Biomedicine‐Driven Precision Orthodontics and Craniofacial Care | | 89 |
| **Letters to the editor, commentaries, or editorial responses lacking original data (N=8)** | | | |
| Ramesh, S. 2017 (221) | A checklist to protect human rights in artificial-intelligence research | | 1 |
| Collins G., 2019 (222) | Reporting of artificial intelligence prediction models | | 2 |
| Sounderajah, V. 2020 (223) | Developing specific reporting guidelines for diagnostic accuracy studies assessing AI interventions: The STARD-AI Steering Group | | 3 |
| Sounderajah V 2021 (224) | A quality assessment tool for artificial intelligence-centered diagnostic test accuracy studies: QUADAS-AI. | | 4 |
| Bartlett, Benjamin.,  2023 (225) | The possibility of AI-induced medical manslaughter: Unexplainable decisions, epistemic vices, and a new dimension of moral luck | | 5 |
| Bacchi S., 2024 (226) | Should this artificial intelligence algorithm be used in my practice now? {A} checklist approach | | 6 |
| Deroncele-Acosta, Angel.,  2024 (227) | Ten Essential Pillars in Artificial Intelligence for University Science Education: A Scoping Review | | 7 |
| Ye, H. 2024 (228) | Some suggestions for 'A checklist for reporting, reading and evaluating Artificial Intelligence Technology Enhanced Learning (AITEL) research in medical education' | | 8 |
| **OTHERS (N=118)** | | | |
| van Trijffel E.,  2005 (229) | Inter-examiner reliability of passive assessment of intervertebral motion in the cervical and lumbar spine: a systematic review. | | 1 |
| deSouza N M.,  2014 (230) | Diffusion-weighted MRI for detecting prostate tumour in men at increased genetic risk. | | 2 |
| Schwartz S A.,  2014 (231) | A double-blind, randomized study to assess the validity of applied kinesiology (AK) as a diagnostic tool and as a nonlocal proximity effect. | | 3 |
| van Loon K.,  2015 (232) | Non-Invasive Continuous Respiratory Monitoring on General Hospital Wards: A Systematic Review. | | 4 |
| Ferraro, P. M.,  2018 (233) | A STARD-compliant prediction model for diagnosing thrombotic microangiopathies. | | 5 |
| Sampieri C L.,  2018 (234) | Matrix metalloproteinases and tissue inhibitors of metalloproteinases in chronic kidney disease and acute kidney injury: a systematic review of the literature. | | 6 |
| Sun R.,  2018 (235) | Fall Risk Prediction in Multiple Sclerosis Using Postural Sway Measures, A Machine Learning Approach | | 7 |
| Gudmundsson H.,  2019 (236) | Clinical decision support system for the management of osteoporosis compared to NOGG guidelines and an osteology specialist: a validation pilot study. | | 8 |
| Shillan D.,  2019 (237) | Use of machine learning to analyse routinely collected intensive care unit data: a systematic review. | | 9 |
| Sun R.,  2019 (238) | Fall Risk Prediction in Multiple Sclerosis Using Postural Sway Measures: A Machine Learning Approach. | | 10 |
| Anderson A.,  2020 (239) | Can Predictive Modeling Tools Identify Patients at High Risk of Prolonged Opioid Use After ACL Reconstruction? | | 11 |
| Baldwin D.,  2020 (240) | External validation of a convolutional neural network artificial intelligence tool to predict malignancy in pulmonary nodules. | | 12 |
| Cresswell K., 2020 (241) | Investigating the use of data-driven artificial intelligence in computerised decision support systems for health and social care: {A} systematic review. | | 13 |
| Low D., 2020 (242) | Automated assessment of psychiatric disorders using speech: A systematic review | | 14 |
| Medina M M.,  2020 (243) | Diagnostic Accuracy of Intracochlear Test Electrode for Acoustic Nerve Monitoring in Vestibular Schwannoma Surgery. | | 15 |
| Miles J.,  2020 (244) | Using machine-learning risk prediction models to triage the acuity of undifferentiated patients entering the emergency care system: a systematic review. | | 16 |
| WongvibulsinS., 2020 (245) | Improving Clinical Translation of Machine Learning Approaches Through Clinician-Tailored Visual Displays of Black Box Algorithms: Development and Validation. | | 17 |
| Alim-MarvastiA.,  2021 (246) | Machine Learning for Localizing Epileptogenic-Zone in the Temporal Lobe: Quantifying the Value of Multimodal Clinical-Semiology and Imaging Concordance. | | 18 |
| Ferreira-Santos D.,  2021 (247) | Enhancing Obstructive Sleep Apnea Diagnosis With Screening Through Disease Phenotypes: Algorithm Development and Validation. Informatics, | | 19 |
| Hamidi F., 2021 (248) | Exploration of {Potential} {miRNA} {Biomarkers} and {Prediction} for {Ovarian} {Cancer} {Using} {Artificial} {Intelligence} | | 20 |
| Ibrahim W.,  2021 (249) | A systematic review of the diagnostic accuracy of volatile organic compounds in airway diseases and their relation to markers of type-2 inflammation. | | 21 |
| Kareemi H.,  2021 (250) | Machine Learning Versus Usual Care for Diagnostic and Prognostic Prediction in the Emergency Department: A Systematic Review. | | 22 |
| Mao-feng W.,  2021 (251) | Development and validation of a novel risk assessment model to estimate the probability of pulmonary embolism in postoperative patients. | | 23 |
| McGenity, C. 2021 (252) | Guidelines for clinical trials using artificial intelligence - SPIRIT-AI and CONSORT-AI | | 24 |
| Møller J.,  2021 (253) | Prediction of risk of acquiring urinary tract infection during hospital stay based on machine-learning: A retrospective cohort study. | | 25 |
| Knoop J.,  2022 (254) | Development and internal validation of a machine learning prediction model for low back pain non-recovery in patients with an acute episode consulting a physiotherapist in primary care. | | 26 |
| Labiste C. C.,  2022 (255) | Systematic review: investigating the added diagnostic value of gadolinium contrast agents for osteomyelitis in the appendicular skeleton. S | | 27 |
| O'Connor M., 2022 (256) | PRAME immunohistochemistry for melanoma diagnosis: A STARD-compliant diagnostic accuracy study | | 28 |
| Vasey B. 2022 (257) | Publisher Correction: Reporting guideline for the early-stage clinical evaluation of decision support systems driven by artificial intelligence: DECIDE-AI. | | 29 |
| Xu X.,  2022 (258) | Web-Based Risk Prediction Tool for an Individual's Risk of HIV and Sexually Transmitted Infections Using Machine Learning Algorithms: Development and External Validation Study. | | 30 |
| Abdulazeem H.,  2023 (259) | A systematic review of clinical health conditions predicted by machine learning diagnostic and prognostic models trained or validated using real‑world primary health care data | | 31 |
| Ajuwon BI.,  2023 (260) | Machine learning prediction models for clinical management of blood-borne viral infections: a systematic review of current applications and future impact. | | 32 |
| Alfieri F.  2023 (261) | Continuous and early prediction of future moderate and severe Acute Kidney Injury in critically ill patients: Development and multi-centric, multi-national external validation of a machine-learning model. | | 33 |
| Chan P. Z.,  2023 (262) | Diagnostic Test Accuracy of artificial intelligence-assisted detection of acute coronary syndrome: A systematic review and meta-analysis. | | 34 |
| da Silva A.,  2023 (263) | Drug-related fall risk in hospitals: a machine learning approach. | | 35 |
| Dosis A.,  2023 (264) | Estimating postoperative mortality in colorectal surgery- a systematic review of risk prediction models. | | 36 |
| Doudesis, D.,  2023 (265) | Machine learning for diagnosis of myocardial infarction using cardiac troponin concentrations. | | 37 |
| Fanni S.C., 2023 (266) | Natural language processing to convert unstructured {COVID}-19 chest-{CT} reports into structured reports | | 38 |
| Gedefaw L.,  2023 (267) | Artificial Intelligence-Assisted Diagnostic Cytology and Genomic Testing for Hematologic Disorders. | | 39 |
| Gokhale S., 2023 (268) | Hospital length of stay prediction for general surgery and total knee arthroplasty admissions: Systematic review and meta-analysis of published prediction models. | | 40 |
| Ishii E.,  2023 (269) | Development, validation, and feature extraction of a deep learning model predicting in- hospital mortality using Japan's largest national ICU database: a validation framework for transparent clinical Artificial Intelligence (cAI) development. | | 41 |
| Jiang D.,  2023 (270) | A prediction model for severe hematological toxicity of BTK inhibitors. | | 42 |
| Smith LA.,  2023 (271) | Machine learning and deep learning predictive models for long-term prognosis in patients with chronic obstructive pulmonary disease: a systematic review and meta-analysis. | | 43 |
| Wang J.,  2023 (272) | Prediction of Suicidal Behaviors in the Middle-aged Population: Machine Learning Analyses of UK Biobank. | | 44 |
| Zhou Y.,  2023 (273) | A systematic review of predictive models for hospital-acquired pressure | | 45 |
| Al-Dhubaibi, M S.,  2024 (274) | “High specificity of PCR in diagnosing mucocutaneous leshminiasis: a systematic review and meta analysis.” | | 46 |
| Chen H.,  2024 (275) | Machine Learning for Prediction of Postoperative Delirium in Adult Patients: A Systematic Review and Meta-analysis. | | 47 |
| Cascella M., 2024 (276) | {AI}-based cancer pain assessment through speech emotion recognition and video facial expressions classification | | 48 |
| Jawadi Z.,  2024 (277) | Predicting in‐hospital mortality among patients admitted with a diagnosis of heart failure: a machine learning approach. | | 49 |
| Ke J.,  2024 (278) | The effect of resampling techniques on the performances of machine learning clinical risk prediction models in the setting of severe class imbalance: development and internal validation in a retrospective cohort. | | 50 |
| Lampe D.,  2024 (279) | How intervention studies measure the effectiveness of medication safety-related clinical decision support systems in primary and long-term care: a systematic review. | | 51 |
| Li H.,  2024 (280) | Interpretable machine learning for the prediction of death risk in patients with acute diquat poisoning. | | 52 |
| Li Y.,  2024 (281) | Mixed-variable graphical modeling framework towards risk prediction of hospital-acquired pressure injury in spinal cord injury individuals. | | 53 |
| Marinkovic  M.,  2024 (282) | Performance and Dimensionality of Pretreatment MRI Radiomics in Rectal Carcinoma Chemoradiotherapy Prediction. | | 54 |
| Mäenpää S M.,  2024 (283) | Diagnostic test accuracy of externally validated convolutional neural network (CNN) artificial intelligence (AI) models for emergency head CT scans - A systematic review. | | 55 |
| Mendoza-Pinto C.,  2024 (284) | Machine learning in the prediction of treatment response in rheumatoid arthritis: A systematic review. | | 56 |
| Mohammadi S.,  2024 (285) | Artificial intelligence in osteoarthritis detection: A systematic review and meta-analysis. | | 57 |
| Restini, F.  2024 (286) | AI tool for predicting MGMT methylation in glioblastoma for clinical decision support in resource limited settings. | | 58 |
| Ruiz-TarrazoX.,  2024 (287) | Validity and reliability of ultrasonographic assessment of femoral and tibial torsion in children and adolescents: a systematic review. | | 59 |
| Shujaat S.,  2024 (288) | Emergence of artificial intelligence for automating cone-beam computed tomography-derived maxillary sinus imaging tasks. A systematic review. | | 60 |
| Tariq R.,  2024 (289) | SYSTEMATIC REVIEW OF MACHINE LEARNING-BASED PREDICTIVE MODELS FOR CLOSTRIDIOIDES DIFFICILE INFECTION. | | 61 |
| Vueghs C.,  2024 (290) | Development and Evaluation of a GPT4-Based Orofacial Pain Clinical Decision Support System. | | 62 |
| Abdelmoteleb S.,  2025 (291) | Evaluating the ability of artificial intelligence to predict suicide: A systematic review of reviews. | | 63 |
| Antunes M.E.., 2025 (292) | Machine learning models for predicting prostate cancer recurrence and identifying potential molecular biomarkers | | 64 |
| Al-Husaini N.,  2025 (293) | Characterizing low femoral neck BMD in Qatar Biobank participants using machine learning models. | | 65 |
| Apurva P.,  2025 (294) | The Efficacy of Artificial Intelligence in the Detection and Management of Atrial Fibrillation. | | 66 |
| Ari F.,  2025 (295) | Internal Validation of a Machine Learning-Based CDSS for Antimicrobial Stewardship. | | 67 |
| Ashfaq M.T.,  2025 (296) | An explainable AI based new deep learning solution for efficient heart disease prediction at early stages | | 68 |
| Bouktif S., 2025 (297) | Explainable {Predictive} {Model} for {Suicidal} {Ideation} {During} {COVID}-19: {Social} {Media} {Discourse} {Study} | | 69 |
| Chen Q.,  2025 (298) | Prediction models for treatment response in migraine: a systematic review and meta‑analysis | | 70 |
| Bayor A., 2025 (299) | Designing Clinical Decision Support Systems (CDSS)-A User-Centered Lens of the Design Characteristics, Challenges, and Implications: Systematic Review | | 71 |
| Constance A.,  2025 (300) | A Data-Driven Intelligent Methodology for Developing Explainable Diagnostic Model for Febrile Diseases. | | 72 |
| Ramwala O., 2025 (301) | ClinValAI: A framework for developing Cloud-based infrastructures for the External Clinical Validation of AI in Medical Imaging. | | 73 |
| da Silva-Filho J E.,  2025 (302) | Deep learning for detecting periapical bone rarefaction in panoramic radiographs: a systematic review and critical assessment. | | 74 |
| Deng L.,  2025 (303) | Relative Fat Mass and Physical Indices as Predictors of Gallstone Formation: Insights From Machine Learning and Logistic Regression. | | 75 |
| Espinoza-Vinces C.,  2025 (304) | Artificial intelligence in headache medicine: between automation and the doctor-patient relationship. A systematic review. | | 76 |
| Fass O Z.,  2025 (305) | Diagnostic Accuracy of Timed Barium Esophagram for Achalasia. | | 77 |
| Flores G. P.,  2025 (306) | Diagnostic Performance of a Computer-aided System for Tuberculosis Screening in Two Philippine Cities. | | 78 |
| Fu Y.,  2025 (307) | Artificial Intelligence in Lymphoma Histopathology: Systematic Review. | | 79 |
| Gao C.,  2025 (308) | Deep learning in pulmonary nodule detection and segmentation: a systematic review. | | 80 |
| Ge X.,  2025 (309) | Comparing machine learning models for predicting preoperative DVT incidence in elderly hypertensive patients with hip fractures: a retrospective analysis | | 81 |
| Haghighat S.,  2025 (310) | Diagnostic accuracy of artificial intelligence for obstructive sleep apnea detection: a systematic review. | | 82 |
| Huang H.,  2025 (311) | Risk prediction models for diabetic retinopathy: a systematic review. | | 83 |
| Jemimah S., 2025 (312) | c-{Triadem}: {A} constrained, explainable deep learning model to identify novel biomarkers in {Alzheimer}'s disease | | 84 |
| Jia J.,  2025 (313) | Risk Factors and Predictive Model for Ischemic Complications in Endovascular Treatment of Intracranial Aneurysms: Insights From a Large Patient Cohort. | | 85 |
| Khaja S., 2025 (314) | Artificial intelligence-powered advancements in atrial fibrillation diagnostics: a systematic review | | 86 |
| Khan Z.,  2025 (315) | Diagnostic Accuracy of IDX-DR for Detecting Diabetic Retinopathy: A Systematic Review and Meta- Analysis. | | 87 |
| Kuang-Ming Kuo.,  2025 (316) | A meta-analysis of the diagnostic test accuracy of artificial intelligence predicting emergency department dispositions. | | 88 |
| Leitão B N.,  2025 (317) | Enhancing Prognostic Signatures in Glioblastoma with Feature Selection and Regularised Cox Regression. | | 89 |
| Liu W.,  2025 (318) | Development and validation of interpretable machine learning models for predicting AKI risk in patients treated with PD-1/PD-L1: a retrospective study. | | 90 |
| Medani M.,  2025 (319) | Leveraging explainable artificial intelligence with ensemble of deep learning model for dementia prediction to enhance clinical decision support systems | | 91 |
| Meiklejohn K.,  2025 (320) | Network-based biomarkers in background electroencephalography in childhood epilepsies-A scoping review and narrative synthesis. | | 92 |
| Mohamed Ahmed, H.  2025 (321) | The Role of Artificial Intelligence in the Prediction of Bariatric Surgery Complications: A Systematic Review. | | 93 |
| Mohamed Dkeen N.,  2025 (322) | Artificial Intelligence Applications in Obstetric Risk Prediction: A Systematic Review of Machine Learning Models for Preeclampsia. | | 94 |
| Tun H., 2025 (323) | Trust in Artificial Intelligence-Based Clinical Decision Support Systems Among Health Care Workers: Systematic Review. | | 95 |
| Muhammad Mohsin Zafar.,  2025 (324) | Enhancing clinical decision support with explainable deep learning framework for C-section forecasting. | | 96 |
| Nguyen R N.,  2025 (325) | Machine Learning Nomogram for Predicting Dengue Shock Syndrome in Pediatric Patients With Dengue Fever in Vietnam. | | 97 |
| Oluwatobi, I. D.,  2025 (326) | Machine Learning in Schizophrenia: A Systematic Review and Meta-Analysis of Diagnostic and Predictive Models. | | 98 |
| Petsiou DP.,  2025 (327) | Effectiveness of Artificial Intelligence in detecting sinonasal pathology using clinical imaging modalities: a systematic review. | | 99 |
| Rao A.,  2025 (328) | A Simple, Interpretable Machine Learning Model Based on Clinical Factors Accurately Predicts Incident Dysplasia or Malignancy in Barrett’s Esophagus. | | 100 |
| Ren B.,  2025 (329) | Interpretable prediction of hospital mortality in bleeding critically ill patients based on machine learning and SHAP. | | 101 |
| Roison A N.,  2025 (330) | Artificial intelligence in symptom management and clinical decision support for palliative care. | | 102 |
| Sharma G.,  2025 (331) | Use of Artificial Intelligence in Adolescents' Mental Health Care: Systematic Scoping Review of Current Applications and Future Directions. | | 103 |
| Shrikrishna, B H.,  2025 (332) | The Application and Diagnostic Accuracy of Artificial Intelligence in Rhinology: A Review. | | 104 |
| Soriano-Arandes Antoni.,  2025 (333) | Implementing Symptom-Based Predictive Models for Early Diagnosis of Pediatric Respiratory Viral Infections. | | 105 |
| Stuke H.,  2025 (334) | Peer Relationships Are a Direct Cause of the Adolescent Mental Health Crisis: Interpretable Machine Learning Analysis of 2 Large Cohort Studies. | | 106 |
| Tomassini S.,  2025 (335) | Multi-Branch CNN-LSTM Fusion Network-Driven System With BERT Semantic Evaluator for Radiology Reporting in Emergency Head CTs. | | 107 |
| Vali M.,  2025 (336) | Machine learning algorithms for predicting PTSD: a systematic review and meta-analysis. | | 108 |
| Wang L.,  2025 (337) | Construction and validation of a risk prediction model for chronic obstructive pulmonary disease (COPD): a cross-sectional study based on the NHANES database from 2009 to 2018. | | 109 |
| Wang X.,  2025 (338) | Risk prediction models for dental caries in children and adolescents: a systematic review and meta-analysis. | | 110 |
| Xiong X.,  2025 (339) | Ten Machine Learning Models for Predicting Preoperative and Postoperative Coagulopathy in Patients With Trauma: Multicenter Cohort Study. | | 111 |
| Yang X.,  2025 (340) | Performance of Artificial Intelligence in Diagnosing Lumbar Spinal Stenosis: A Systematic Review and Meta-Analysis. | | 112 |
| Yuan S.,  2025 (341) | AI-Powered early warning systems for clinical deterioration significantly improve patient outcomes: a meta-analysis. | | 113 |
| Zhang J.,  2025 (342) | In-depth analysis of the risk factors for persistent severe acute respiratory syndrome coronavirus 2 infection and construction of predictive models: an exploratory research study. | | 114 |
| Zmudzki F.,  2025 (343) | Machine Learning Clinical Decision Support for Interdisciplinary Multimodal Chronic Musculoskeletal Pain Treatment: Prospective Pilot Study of Patient Assessment and Prognostic Profile Validation. | | 115 |
| Lekadir K., 2025 (344) | FUTURE-AI: international consensus guideline for trustworthy and deployable artificial intelligence in healthcare | | 116 |
| Raghunathan K., 2025 (345) | Using artificial intelligence to improve healthcare delivery in select allied health disciplines: a scoping review protocol | | 117 |
| Petrella F., 2025 (346) | Artificial Intelligence in Oncologic Thoracic Surgery: Clinical Decision Support and Emerging Applications | | 118 |

**References**

1. Andaur Navarro CL, Damen JAAG, Takada T, Nijman SWJ, Dhiman P, Ma J, et al. Protocol for a systematic review on the methodological and reporting quality of prediction model studies using machine learning techniques. BMJ Open. 2020 Nov;10(11):e038832. doi:10.1136/bmjopen-2020-038832

2. Nagendrababu V, Abbott P, Duncan HF, Fouad AF, Kruse C, Patel S, et al. Preferred Reporting Items for Diagnostic Accuracy Studies in Endodontics (PRIDASE) guidelines: a development protocol. Int Endodontic J. 2021 Jul;54(7):1051–5. doi:10.1111/iej.13497

3. Liu N, Xie F, Fahad Javaid Siddiqui, Ho AFW, Chakraborty B, Gayathri Devi Nadarajan, et al. Leveraging Large-Scale Electronic Health Records and Interpretable Machine Learning for Clinical Decision Making at the Emergency Department: Protocol for System Development and Validation. JMIR Research Protocols. 2022 Mar;11(3). Located at: Coronavirus Research Database; ProQuest Central; 2645679977. doi:10.2196/34201

4. Satchwell L, Wedlake L, Greenlay E, Li X, Messiou C, Glocker B, et al. Development of machine learning support for reading whole body diffusion-weighted MRI (WB-MRI) in myeloma for the detection and quantification of the extent of disease before and after treatment (MALIMAR): protocol for a cross-sectional diagnostic test accuracy study. BMJ Open. 2022 Oct 5;12(10):e067140. doi:10.1136/bmjopen-2022-067140 PubMed PMID: 36198471; PubMed Central PMCID: PMC9535185.

5. Liu V, Koskela TH, Kaila M. User-Initiated Symptom Assessment With an Electronic Symptom Checker: Protocol for a Mixed Methods Validation Study. JMIR Research Protocols. 2023;12. Located at: Coronavirus Research Database; ProQuest Central; 2918516786. doi:10.2196/41423

6. Fu H, Novak A, Robert D, Kumar S, Tanamala S, Oke J, et al. AI assisted reader evaluation in acute CT head interpretation (AI-REACT): protocol for a multireader multicase study. BMJ Open. 2024 Feb;14(2):e079824. doi:10.1136/bmjopen-2023-079824

7. Hou Z, Yang Y, Deng B, Gao G, Li M, Liu X, et al. Development, validation and economic evaluation of a machine learning algorithm for predicting the probability of kidney damage in patients with hyperuricaemia: protocol for a retrospective study. BMJ Open. 2024 Nov 28;14(11):e086032. doi:10.1136/bmjopen-2024-086032 PubMed PMID: 39613447; PubMed Central PMCID: PMC11605815.

8. Howell F, Novak A, Dennis R, Kumar S, Tanamala S, Oke J, et al. AI assisted reader evaluation in acute CT head interpretation (AI-REACT): protocol for a multireader multicase study. BMJ Open. 2024;14(2). Located at: ProQuest Central; 2925253968. doi:10.1136/bmjopen-2023-079824

9. Sguanci M, Mancin S, Piredda M, De Marinis MG. Protocol for conducting a systematic review on diagnostic accuracy in clinical research. MethodsX. 2024 Jun;12:102569. doi:10.1016/j.mex.2024.102569

10. Chitrapady S, Rajendran R, K H, M U T, Rashid M, Poojari PG, et al. Machine-learning-based artificial intelligence tools for the diagnosis of tropical fevers: a systematic review and meta-analysis protocol of diagnostic test accuracy. BMJ Open. 2025 Aug 25;15(8):e102158. doi:10.1136/bmjopen-2025-102158 PubMed PMID: 40854843; PubMed Central PMCID: PMC12382579.

11. Olwendo AO, Kikuvi G, Karanja S. Development and validation of a predictive model for new HIV infection screening among persons 15 years and above in primary healthcare settings in Kenya: a study protocol. BMJ Health Care Inform. 2025 Aug 22;32(1). doi:10.1136/bmjhci-2024-101419 PubMed PMID: 40846507; PubMed Central PMCID: PMC12374640.

12. Lazaridou A, Sivakumar S, Rodriguez Cetina Biefer H, Weilenmann S, Princip M, Zuccarella-Hackl C, et al. Predictive modelling of clinically significant depressive symptoms after coronary artery bypass graft surgery: protocol for a multicentre observational study in two Swiss hospitals (the PsyCor study). BMJ Open. 2025 Sep 5;15(9):e108061. doi:10.1136/bmjopen-2025-108061 PubMed PMID: 40912718; PubMed Central PMCID: PMC12414220.

13. Pereira Salgado D, De Queiroz CV, Naves ELM, Qiao Y, Fallon S. Protocol for evaluation of a virtual wheelchair simulator in assessing mobility skills and cognitive abilities in diverse populations: A multicentric mixed-methods pilot study. Gomes Costa RR, editor. PLoS One. 2025 Jun 6;20(6):e0325186. doi:10.1371/journal.pone.0325186

14. Tonde B, Metogara Mohamed Traore, Landa P, Côté A, Laberge M. Predictive modelling methods of hospital readmission risks for patients with chronic obstructive pulmonary disease (COPD): a systematic review protocol. BMJ Open. 2025;15(4). Located at: Coronavirus Research Database; ProQuest Central; 3200387263. doi:10.1136/bmjopen-2024-093771

15. El Wadia H, Buh A, Kabli AO, Karim M, Biyani N, Shorr R, et al. Effectiveness of predictive scoring systems in predicting mortality in relation to baseline kidney function in adult intensive care unit patients: a systematic review protocol. BMJ Open. 2025 Jul 17;15(7):e098192. doi:10.1136/bmjopen-2024-098192 PubMed PMID: 40675642; PubMed Central PMCID: PMC12273111.

16. Alpern ER, Scott HF, Balamuth F, Chamberlain JM, Depinet H, Bajaj L, et al. Derivation and Validation of Predictive Models for Early Pediatric Sepsis. JAMA Pediatr. 2025 Dec 1;179(12):1318. doi:10.1001/jamapediatrics.2025.3892

17. Gupta A, Rajamohan N, Bansal B, Chaudhri S, Chandarana H, Bagga B. Applications of artificial intelligence in abdominal imaging. Abdom Radiol. 2025 May 26;50(12):6172–91. doi:10.1007/s00261-025-04990-0

18. Palermi S, Vecchiato M, Ng FS, Attia Z, Cho Y, Anselmino M, et al. Artificial intelligence and the electrocardiogram: A modern renaissance. European Journal of Internal Medicine. 2025 Oct;140:106329. doi:10.1016/j.ejim.2025.04.036

19. Hu Y, Liu J, Jiang W. Large language models in nephrology: applications and challenges in chronic kidney disease management. Renal Failure. 2025 Dec 31;47(1):2555686. doi:10.1080/0886022X.2025.2555686

20. Altman DG, Moher D. Elaboración de directrices para la publicación de investigación biomédica: proceso y fundamento científico. Medicina Clínica. 2005 Dec;125:8–13. doi:10.1016/S0025-7753(05)72203-X

21. Cleophas T, Droogendijk J, Van Ouwerkerk B. Validating Diagnostic Tests, Correct and Incorrect Methods, New Developments. CCP. 2008 May 1;3(2):70–6. doi:10.2174/157488408784293697

22. Manchikanti L. Evidence-Based Medicine, SystematicReviews, and Guidelines in InterventionalPain Management: Part 5. DiagnosticAccuracy Studies. Pain Phys. 2009 May 14;3;12(3;5):517–40. doi:10.36076/ppj.2009/12/517

23. Vandenbroucke JP. STREGA, STROBE, STARD, SQUIRE, MOOSE, PRISMA, GNOSIS, TREND, ORION, COREQ, QUOROM, REMARK… and CONSORT: for whom does the guideline toll? Journal of Clinical Epidemiology. 2009 Jun;62(6):594–6. doi:10.1016/j.jclinepi.2008.12.003

24. Simera I. Get the content right: following reporting guidelines will make your research paper more complete, transparent and usable. J Pak Med Assoc. 2013 Feb;63(2):283–5. PubMed PMID: 23894916.

25. Glasziou P, Altman DG, Bossuyt P, Boutron I, Clarke M, Julious S, et al. Reducing waste from incomplete or unusable reports of biomedical research. The Lancet. 2014 Jan;383(9913):267–76. doi:10.1016/S0140-6736(13)62228-X

26. Dinga R, Penninx BWJH, Veltman DJ, Schmaal L, Marquand AF. Beyond accuracy: Measures for assessing machine learning models, pitfalls and guidelines [Internet]. Neuroscience; 2019 [cited 2025 Nov 25]. Available from: http://biorxiv.org/lookup/doi/10.1101/743138 doi:10.1101/743138

27. Grech V. Write a Scientific Paper (WASP): Guidelines for reporting medical research. Early Human Development. 2019 Jul;134:55–7. doi:10.1016/j.earlhumdev.2019.05.014

28. Itani S, Lecron F, Fortemps P. Specifics of medical data mining for diagnosis aid: A survey. Expert Systems with Applications. 2019 Mar;118:300–14. doi:10.1016/j.eswa.2018.09.056

29. De Rooij M, Weeda W. Cross-Validation: A Method Every Psychologist Should Know. Advances in Methods and Practices in Psychological Science. 2020 Jun;3(2):248–63. doi:10.1177/2515245919898466

30. Morgenstern JD, Buajitti E, Meghan O’Neill, Piggott T, Goel V, Fridman D, et al. Predicting population health with machine learning: a scoping review. BMJ Open. 2020;10(10). Located at: ProQuest Central; 2454560127. doi:10.1136/bmjopen-2020-037860

31. Schwendicke F, Samek W, Krois J. Artificial Intelligence in Dentistry: Chances and Challenges. J Dent Res. 2020 Jul;99(7):769–74. doi:10.1177/0022034520915714 PubMed PMID: 32315260; PubMed Central PMCID: PMC7309354.

32. Young AT, Xiong M, Pfau J, Keiser MJ, Wei ML. Artificial Intelligence in Dermatology: A Primer. J Invest Dermatol. 2020 Aug;140(8):1504–12. doi:10.1016/j.jid.2020.02.026 PubMed PMID: 32229141.

33. Clausen CE, Leventhal BL, Nytrø Ø, Koposov R, Westbye OS, Thomas Brox Røst, et al. Clinical Decision Support Systems: An Innovative Approach to Enhancing Child and Adolescent Mental Health Services. Journal of the American Academy of Child and Adolescent Psychiatry. 2021 May;60(5):562–5. Located at: ProQuest Central; 2848907834. doi:10.1016/j.jaac.2020.09.018

34. Clement J, Maldonado AQ. Augmenting the Transplant Team With Artificial Intelligence: Toward Meaningful AI Use in Solid Organ Transplant. Front Immunol. 2021;12:694222. doi:10.3389/fimmu.2021.694222 PubMed PMID: 34177958; PubMed Central PMCID: PMC8226178.

35. Sitch AJ, Dekkers OM, Scholefield BR, Takwoingi Y. Introduction to diagnostic test accuracy studies. European Journal of Endocrinology. 2021 Feb;184(2):E5–9. doi:10.1530/EJE-20-1239

36. Bazoukis G, Hall J, Loscalzo J, Antman EM, Fuster V, Armoundas AA. The inclusion of augmented intelligence in medicine: A framework for successful implementation. Cell Rep Med. 2022 Jan 18;3(1):100485. doi:10.1016/j.xcrm.2021.100485 PubMed PMID: 35106506; PubMed Central PMCID: PMC8784713.

37. Cassinelli Petersen GI, Shatalov J, Verma T, Brim WR, Subramanian H, Brackett A, et al. Machine Learning in Differentiating Gliomas from Primary CNS Lymphomas: A Systematic Review, Reporting Quality, and Risk of Bias Assessment. AJNR Am J Neuroradiol. 2022 Apr;43(4):526–33. doi:10.3174/ajnr.A7473 PubMed PMID: 35361577; PubMed Central PMCID: PMC8993193.

38. Collins JW, Marcus HJ, Ghazi A, Sridhar A, Hashimoto D, Hager G, et al. Ethical implications of AI in robotic surgical training: A Delphi consensus statement. Eur Urol Focus. 2022 Mar;8(2):613–22. doi:10.1016/j.euf.2021.04.006 PubMed PMID: 33941503.

39. Crossnohere NL, Elsaid M, Paskett J, Bose-Brill S, Bridges JFP. Guidelines for Artificial Intelligence in Medicine: Literature Review and Content Analysis of Frameworks. J Med Internet Res. 2022 Aug 25;24(8):e36823. doi:10.2196/36823 PubMed PMID: 36006692; PubMed Central PMCID: PMC9459836.

40. Fanciullo C, Gitto S, Carlicchi E, Albano D, Messina C, Sconfienza LM. Radiomics of Musculoskeletal Sarcomas: A Narrative Review. Journal of Imaging. 2022;8(2):45. Located at: ProQuest Central; 2632816606. doi:10.3390/jimaging8020045

41. Khoury P, Srinivasan R, Kakumanu S, Ochoa S, Keswani A, Sparks R, et al. A Framework for Augmented Intelligence in Allergy and Immunology Practice and Research-A Work Group Report of the AAAAI Health Informatics, Technology, and Education Committee. J Allergy Clin Immunol Pract. 2022 May;10(5):1178–88. doi:10.1016/j.jaip.2022.01.047 PubMed PMID: 35300959; PubMed Central PMCID: PMC9205719.

42. Lu J, Sattler A, Wang S, Khaki AR, Callahan A, Fleming S, et al. Considerations in the Reliability and Fairness Audits of Predictive Models for Advance Care Planning [Internet]. 2022 [cited 2025 Sep 25]. Available from: http://medrxiv.org/lookup/doi/10.1101/2022.07.10.22275967 doi:10.1101/2022.07.10.22275967

43. Misiak M, Kurpas D. Checklists for reporting research in Advances in Clinical and Experimental Medicine: How to choose a proper one for your manuscript? Adv Clin Exp Med. 2022 Oct 24;31(10):1065–72. doi:10.17219/acem/155921

44. Uddhav T. Kumbhar EAl. Explainable AI-Powered IoT Systems for Predictive and Preventive Healthcare - A Framework for Personalized Health Management and Wellness Optimization. jes. 2024 Jan 25;19(3):23–31. doi:10.52783/jes.648

45. van Velzen M, de Graaf-Waar HI, Ubert T, van der Willigen RF, Muilwijk L, Schmitt MA, et al. 21st century (clinical) decision support in nursing and allied healthcare. Developing a learning health system: a reasoned design of a theoretical framework. BMC Med Inform Decis Mak. 2023 Dec 5;23(1):279. doi:10.1186/s12911-023-02372-4 PubMed PMID: 38053104; PubMed Central PMCID: PMC10699040.

46. Dirnfeld R, De Donato L, Somma A, Azari MS, Marrone S, Flammini F, et al. Integrating AI and DTs: challenges and opportunities in railway maintenance application and beyond. SIMULATION. 2024 Sep;100(9):903–17. doi:10.1177/00375497241229756

47. Gatineau G, Shevroja E, Vendrami C, Gonzalez-Rodriguez E, Leslie WD, Lamy O, et al. Development and reporting of artificial intelligence in osteoporosis management. J Bone Miner Res. 2024 Oct 29;39(11):1553–73. doi:10.1093/jbmr/zjae131 PubMed PMID: 39163489; PubMed Central PMCID: PMC11523092.

48. Grech V, Eldawlatly AA. STROBE, CONSORT, PRISMA, MOOSE, STARD, SPIRIT, and other guidelines – Overview and application. Saudi Journal of Anaesthesia. 2024 Jan;18(1):137–41. doi:10.4103/sja.sja_545_23

49. Hogg HDJ, Martindale APL, Liu X, Denniston AK. Clinical Evaluation of Artificial Intelligence-Enabled Interventions. Invest Ophthalmol Vis Sci. 2024 Aug 1;65(10):10. doi:10.1167/iovs.65.10.10 PubMed PMID: 39106058; PubMed Central PMCID: PMC11309043.

50. Loftus TJ, Balch JA, Marquard JL, Ray JM, Alper BS, Ojha N, et al. Longitudinal clinical decision support for assessing decisions over time: State-of-the-art and future directions. Digital Health. 2024 Jan;10. Located at: ProQuest Central; 3149773673. doi:10.1177/20552076241249925

51. Piffer S, Ubaldi L, Tangaro S, Retico A, Talamonti C. Tackling the small data problem in medical image classification with artificial intelligence: a systematic review. Prog Biomed Eng. 2024 Jul 1;6(3):032001. doi:10.1088/2516-1091/ad525b

52. Straus Takahashi M., Donnelly L.F., Siala S. Artificial intelligence: a primer for pediatric radiologists. Pediatr Radiol. 2024;54(13):2127 EP – 2142. Located at: Ovid Emcare <2021 to 2025 Week 28>. doi:10.1007/s00247-024-06098-x

53. Valentina Tamayo Velasquez, Chang J, Waddell A. The development of early warning scores or alerting systems for the prediction of adverse events in psychiatric patients: a scoping review. BMC Psychiatry. 2024;24:1–9. Located at: ProQuest Central; 3126415988. doi:10.1186/s12888-024-06052-z

54. Warren BE, Bilbily A, Gichoya JW, Conway A, Li B, Fawzy A, et al. An Introductory Guide to Artificial Intelligence in Interventional Radiology: Part 1 Foundational Knowledge. Can Assoc Radiol J. 2024 Aug;75(3):558–67. doi:10.1177/08465371241236376

55. Alderman JE, Palmer J, Laws E, McCradden MD, Ordish J, Ghassemi M, et al. Tackling algorithmic bias and promoting transparency in health datasets: the STANDING Together consensus recommendations. Lancet Digit Health. 2025 Jan;7(1):e64–88. doi:10.1016/S2589-7500(24)00224-3 PubMed PMID: 39701919; PubMed Central PMCID: PMC11668905.

56. Bansal RK, Arya Ashtha, Singh Birmohan, Singla Mamta, Gupta S. Role of Artificial Intelligence and Machine Learning in Conservative Dentistry and Endodontics: A Review. Cureus. 2025;17(7):11. Located at: ProQuest Central; 3244982377. doi:10.7759/cureus.88515

57. Carlson DE, Chavarriaga R, Liu Y, Lotte F, Lu BL. The NERVE-ML (neural engineering reproducibility and validity essentials for machine learning) checklist: ensuring machine learning advances neural engineering(). J Neural Eng. 2025 Mar 27;22(2). doi:10.1088/1741-2552/adbfbd PubMed PMID: 40073450; PubMed Central PMCID: PMC11948487.

58. Hurkmans C, Bibault JE, Clementel E, Dhont J, van Elmpt W, Kantidakis G, et al. Assessment of bias in scoring of AI-based radiotherapy segmentation and planning studies using modified TRIPOD and PROBAST guidelines as an example. Radiother Oncol. 2024 May;194:110196. doi:10.1016/j.radonc.2024.110196 PubMed PMID: 38432311.

59. Critelli B, Hassan A, Lahooti I, Noh L, Park JS, Tong K, et al. A systematic review of machine learning-based prognostic models for acute pancreatitis: Towards improving methods and reporting quality. Singal A, editor. PLoS Med. 2025 Feb 24;22(2):e1004432. doi:10.1371/journal.pmed.1004432

60. Choi J. Artificial intelligence in surgery research: Successfully implementing AI clinical decision support models. J Trauma Acute Care Surg. 2025 Oct 1;99(4):518–21. doi:10.1097/TA.0000000000004725 PubMed PMID: 40604360.

61. Park Dogeun, So Kwangsub, Prabhakar SK, Kim C, Lee Jae Jun, Sohn Jong-Hee, et al. Early warning score and feasible complementary approach using artificial intelligence-based bio-signal monitoring system: a review. Biomedical Engineering Letters. 2025;15(4):717–34. Located at: ProQuest Central; 3227041277. doi:10.1007/s13534-025-00486-4

62. Du H, Yu J, Chen D, Wu J, Xue E, Zhou Y, et al. Big Data-Driven Health Portraits for Personalized Management in Noncommunicable Diseases: Scoping Review. J Med Internet Res. 2025 Jun 5;27:e72636. doi:10.2196/72636 PubMed PMID: 40472355; PubMed Central PMCID: PMC12179573.

63. El Arab Rabie Adel, Al Moosa Omayma Abdulaziz, Albahrani Zahraa, Alkhalil Israa, Somerville J, Abuadas Fuad. Integrating Artificial Intelligence into Perinatal Care Pathways: A Scoping Review of Reviews of Applications, Outcomes, and Equity. Nursing Reports. 2025;15(8):281. Located at: ProQuest Central; 3244048151. doi:10.3390/nursrep15080281

64. Gallifant J, Afshar M, Ameen S, Aphinyanaphongs Y, Chen S, Cacciamani G, et al. The TRIPOD-LLM reporting guideline for studies using large language models. Nat Med. 2025 Jan;31(1):60–9. doi:10.1038/s41591-024-03425-5 PubMed PMID: 39779929; PubMed Central PMCID: PMC12104976.

65. Garcia CA, Reed KA, Lantz E, Day P, Zarella MD, Hart SN, et al. Establishing a comprehensive artificial intelligence lifecycle framework for laboratory medicine and pathology: A series introduction. Am J Clin Pathol. 2025 Sep 9;164(3):424–37. doi:10.1093/ajcp/aqaf069 PubMed PMID: 40650598.

66. Kellerhuis BE, Jenniskens K, Kusters MPT, Schuit E, Hooft L, Moons KGM, et al. Expert panel as reference standard procedure in diagnostic accuracy studies: a systematic scoping review and methodological guidance. Diagn Progn Res. 2025 May 13;9(1):12. doi:10.1186/s41512-025-00195-7

67. Kim DH. Personalized Medical Approach in Gastrointestinal Surgical Oncology: Current Trends and Future Perspectives. Journal of Personalized Medicine. 2025;15(5):175. Located at: Coronavirus Research Database; ProQuest Central; 3212027286. doi:10.3390/jpm15050175

68. Kumar R, Kiran M, Sporn K, Paladugu Phani, Khanna Akshay, Gowda Chirag, et al. Emerging Diagnostic Approaches for Musculoskeletal Disorders: Advances in Imaging, Biomarkers, and Clinical Assessment. Diagnostics. 2025;15(13):1648. Located at: ProQuest Central; 3229142330. doi:10.3390/diagnostics15131648

69. Mann J, Lyons M, O’Rourke J, Davies S. Machine learning or traditional statistical methods for predictive modelling in perioperative medicine: A narrative review. Journal of Clinical Anesthesia. 2025 Mar;102. Located at: ProQuest Central; 3172161979. doi:10.1016/j.jclinane.2025.111782

70. Nivethitha V, Daniel RA, Surya BN, Logeswari G. Empowering public health: Leveraging AI for early detection, treatment, and disease prevention in communities - A scoping review. Journal of Postgraduate Medicine. 2025 Jun;71(2):74–81. Located at: ProQuest Central; 3221018000. doi:10.4103/jpgm.jpgm_634_24

71. Obeagu EI, Ezeanya CU, Ogenyi FC, Ifu DD. Big data analytics and machine learning in hematology: Transformative insights, applications and challenges. Medicine. 2025 Mar 7;104(10):e41766. doi:10.1097/MD.0000000000041766

72. Radulescu R mihai. The Role of Artificial Intelligence in Personalized Medicine: A Computer Science Perspective. Applied Medical Informatics [Internet]. 2025 [cited 2025 Nov 25];47(1):S86. Available from: https://www.proquest.com/docview/3218517845/abstract/38D6741CAE146C7PQ/1

73. Ramwala OA, Lowry KP, Hippe DS, Unrath MPN, Nyflot MJ, Mooney SD, et al. ClinValAI: A framework for developing Cloud-based infrastructures for the External Clinical Validation of AI in Medical Imaging. Pac Symp Biocomput. 2025;30:215–28. doi:10.1142/9789819807024_0016 PubMed PMID: 39670372; PubMed Central PMCID: PMC12240695.

74. Samaranayake L, Tuygunov N, Schwendicke F, Osathanon T, Khurshid Z, Boymuradov SA, et al. The Transformative Role of Artificial Intelligence in Dentistry: A Comprehensive Overview. Part 1: Fundamentals of AI, and its Contemporary Applications in Dentistry. Int Dent J. 2025 Apr;75(2):383–96. doi:10.1016/j.identj.2025.02.005 PubMed PMID: 40074616; PubMed Central PMCID: PMC11976540.

75. Leivaditis Vasileios, Maniatopoulos Andreas Antonios, Lausberg Henning, Francesk M, Papatriantafyllou Athanasios, Liolis Elias, et al. Artificial Intelligence in Thoracic Surgery: A Review Bridging Innovation and Clinical Practice for the Next Generation of Surgical Care. Journal of Clinical Medicine. 2025;14(8):2729. Located at: ProQuest Central; 3194615852. doi:10.3390/jcm14082729

76. Vallée A. Digital Twins for Personalized Medicine Require Epidemiological Data and Mathematical Modeling: Viewpoint. J Med Internet Res. 2025 Aug 5;27:e72411. doi:10.2196/72411

77. Abbara S, Crabol Y, De Bouillé JG, Dinh A, Morquin D. Artificial intelligence and infectious diseases: Scope and perspectives. Infectious Diseases Now. 2025 Nov;55(7):105131. doi:10.1016/j.idnow.2025.105131

78. Opel N, Breakspear M. Transforming mental health research and care through artificial intelligence. Science. 2026 Jan 15;391(6782):249–58. Located at: ProQuest Central; 3293411057. doi:10.1126/science.adz9193

79. Wiest IC, Bhat M, Clusmann J, Schneider CV, Jiang X, Kather JN. Large language models for clinical decision support in gastroenterology and hepatology. Nature Reviews Gastroenterology & Hepatology. 2025 Nov;22(11):773–87. Located at: ProQuest Central; 3265686352. doi:10.1038/s41575-025-01108-1

80. Du Y, Yang P, Liu Y, Deng C, Li X. Artificial intelligence in chronic disease self-management: current applications and future directions. Front Public Health. 2025 Nov 20;13:1689911. doi:10.3389/fpubh.2025.1689911

81. Sakamoto A, Kaneko T, Sato E, Fujita W, Nakamura Y, Yokotsuka N, et al. Artificial intelligence in echocardiography: current applications and future perspectives. J Echocardiogr. 2025 Dec;23(4):231–40. doi:10.1007/s12574-025-00703-0

82. Maznyczka A, Nuis RJ, Shiri I, Ternacle J, Garot P, Van Den Dorpel MMP, et al. Artificial Intelligence in Valvular Heart Disease. JACC: Cardiovascular Interventions. 2025 Oct;18(20):2439–57. doi:10.1016/j.jcin.2025.08.031

83. Gong EJ, Woo J, Lee JJ, Bang CS. Role of artificial intelligence in gastric diseases. World J Gastroenterol. 2025 Oct 7;31(37). doi:10.3748/wjg.v31.i37.111327

84. Bossuyt PM, Reitsma JB, Bruns DE, Gatsonis CA, Glasziou PP, Irwig LM, et al. Towards Complete and Accurate Reporting of Studies of Diagnostic Accuracy: The STARD Initiative. Clinical Chemistry. 2003 Jan 1;49(1):1–6. doi:10.1373/49.1.1

85. Bossuyt PM, Reitsma JB, Bruns DE, Gatsonis CA, Glasziou PP, Irwig LM, et al. Towards complete and accurate reporting of studies of diagnostic accuracy: the STARD initiative. Ann Clin Biochem. 2003 Jul 1;40(4):357–63. doi:10.1258/000456303766476986

86. Bossuyt PM, Reitsma JB, Bruns DE, Gatsonis CA, Glasziou PP, Irwig LM, et al. The STARD Statement for Reporting Studies of Diagnostic Accuracy: Explanation and Elaboration. Ann Intern Med. 2003 Jan 7;138(1):W1-12. doi:10.7326/0003-4819-138-1-200301070-00012-w1

87. Ell PJ. STARD and CONSORT: time for reflection. Eur J Nucl Med Mol Imaging. 2003 Jun;30(6):803–4. doi:10.1007/s00259-003-1218-x

88. Bossuyt PM. Towards complete and accurate reporting of studies of diagnostic accuracy: the STARD initiative. Family Practice. 2004 Feb 1;21(1):4–10. doi:10.1093/fampra/cmh103

89. Altman DG, Bossuyt PMM. Estudios de precisión diagnóstica (STARD) y pronóstica (REMARK). Medicina Clínica. 2005 Dec;125:49–55. doi:10.1016/S0025-7753(05)72210-7

90. Cobo E, Selva-O’Callagham A, Ribera JM, Cardellach F, Dominguez R, Vilardell M. Statistical Reviewers Improve Reporting in Biomedical Articles: A Randomized Trial. Scherer R, editor. PLoS ONE. 2007 Mar 28;2(3):e332. doi:10.1371/journal.pone.0000332

91. Mundi R, Chaudhry H, Singh I, Bhandari M. Checklists to improve the quality of the orthopaedic literature. Indian J Orthop. 2008;42(2):150. doi:10.4103/0019-5413.40251

92. Verbeek J. Moose Consort Strobe and Miame Stard Remark or how can we improve the quality of reporting studies. Scand J Work Environ Health. 2008 Jun;34(3):165–7. doi:10.5271/sjweh.1239

93. Meerpohl J, Blümle A, Antes G, Elm EV. Leitlinien für Forschungsberichte sind auch für Leser medizinischer Fachartikel hilfreich. Dtsch med Wochenschr. 2009 Oct;134(41):2078–83. doi:10.1055/s-0029-1237560

94. Areia M, Soares M, Dinis-Ribeiro M. Quality reporting of endoscopic diagnostic studies in gastrointestinal journals: where do we stand on the use of the STARD and CONSORT statements? Endoscopy. 2010 Feb 5;42(02):138–47. doi:10.1055/s-0029-1243846

95. Perneger TV. Citation analysis of identical consensus statements revealed journal-related bias. Journal of Clinical Epidemiology. 2010 Jun;63(6):660–4. doi:10.1016/j.jclinepi.2009.09.012

96. Costa LOP, Maher CG, Lopes AD, Noronha MAD, Costa LCM. Transparent reporting of studies relevant to physical therapy practice. Rev bras fisioter. 2011 Aug;15(4):267–71. doi:10.1590/S1413-35552011005000009

97. Meerpohl JJ, Wolff RF, Antes G, Von Elm E. Are pediatric Open Access journals promoting good publication practice? An analysis of author instructions. BMC Pediatr. 2011 Dec;11(1):27. doi:10.1186/1471-2431-11-27

98. Selman TJ, Morris RK, Zamora J, Khan KS. The quality of reporting of primary test accuracy studies in obstetrics and gynaecology: application of the STARD criteria. BMC Women’s Health. 2011 Dec;11(1):8. doi:10.1186/1472-6874-11-8

99. Hirst A, Altman DG. Are Peer Reviewers Encouraged to Use Reporting Guidelines? A Survey of 116 Health Research Journals. Cameron DW, editor. PLoS ONE. 2012 Apr 27;7(4):e35621. doi:10.1371/journal.pone.0035621

100. Stevens A, Shamseer L, Weinstein E, Yazdi F, Turner L, Thielman J, et al. Relation of completeness of reporting of health research to journals’ endorsement of reporting guidelines: systematic review. BMJ. 2014 Jun 25;348(jun25 2):g3804–g3804. doi:10.1136/bmj.g3804

101. Bossuyt PM, Reitsma JB, Bruns DE, Gatsonis CA, Glasziou PP, Irwig L, et al. STARD 2015: An Updated List of Essential Items for Reporting Diagnostic Accuracy Studies. Radiology. 2015 Dec;277(3):826–32. doi:10.1148/radiol.2015151516

102. Moreno-Ramírez D, Arias-Santiago S, Nagore E, Gilaberte Y. CONSORT, STROBE y STARD. Instrumentos de ayuda para la publicación de resultados de la investigación. Actas Dermo-Sifiliográficas. 2015 Mar;106(2):79–81. doi:10.1016/j.ad.2014.11.003

103. Cohen JF, Korevaar DA, Altman DG, Bruns DE, Gatsonis CA, Hooft L, et al. STARD 2015 guidelines for reporting diagnostic accuracy studies: explanation and elaboration. BMJ Open. 2016 Nov;6(11):e012799. doi:10.1136/bmjopen-2016-012799

104. Johansen M, Thomsen SF. Guidelines for Reporting Medical Research: A Critical Appraisal. International Scholarly Research Notices. 2016 Mar 22;2016:1–7. doi:10.1155/2016/1346026

105. Korevaar DA, Cohen JF, Reitsma JB, Bruns DE, Gatsonis CA, Glasziou PP, et al. Updating standards for reporting diagnostic accuracy: the development of STARD 2015. Res Integr Peer Rev. 2016 Dec;1(1):7. doi:10.1186/s41073-016-0014-7

106. Sims MT, Henning NM, Wayant CC, Vassar M. Do emergency medicine journals promote trial registration and adherence to reporting guidelines? A survey of “Instructions for Authors.” Scand J Trauma Resusc Emerg Med. 2016 Dec;24(1):137. doi:10.1186/s13049-016-0331-3

107. McGrath TA, Alabousi M, Skidmore B, Korevaar DA, Bossuyt PMM, Moher D, et al. Recommendations for reporting of systematic reviews and meta-analyses of diagnostic test accuracy: a systematic review. Syst Rev. 2017 Dec;6(1):194. doi:10.1186/s13643-017-0590-8

108. Toews I, Binder N, Wolff RF, Toprak G, Von Elm E, Meerpohl JJ. Guidance in author instructions of hematology and oncology journals: A cross sectional and longitudinal study. Scherer RW, editor. PLoS ONE. 2017 Apr 28;12(4):e0176489. doi:10.1371/journal.pone.0176489

109. Wayant C, Smith C, Sims M, Vassar M. Hematology journals do not sufficiently adhere to reporting guidelines: a systematic review. Journal of Thrombosis and Haemostasis. 2017 Apr;15(4):608–17. doi:10.1111/jth.13637

110. Hong PJ, Korevaar DA, McGrath TA, Ziai H, Frank R, Alabousi M, et al. Reporting of imaging diagnostic accuracy studies with focus on MRI subgroup: Adherence to STARD 2015. Magnetic Resonance Imaging. 2018 Feb;47(2):523–44. doi:10.1002/jmri.25797

111. Jin Y, Sanger N, Shams I, Luo C, Shahid H, Li G, et al. Does the medical literature remain inadequately described despite having reporting guidelines for 21 years? &ndash; A systematic review of reviews: an update. JMDH. 2018 Sep;Volume 11:495–510. doi:10.2147/JMDH.S155103

112. Mackinnon S, Drozdowska BA, Hamilton M, Noel-Storr AH, McShane R, Quinn T. Are methodological quality and completeness of reporting associated with citation-based measures of publication impact? A secondary analysis of a systematic review of dementia biomarker studies. BMJ Open. 2018 Mar;8(3):e020331. doi:10.1136/bmjopen-2017-020331

113. Cooper CM, Gray H, Barcenas L, Torgerson T, Checketts JX, Vassar M. An Evaluation of Reporting Guidelines and Clinical Trial Registry Requirements Among Addiction Medicine Journals. Journal of Osteopathic Medicine. 2020 Dec 1;120(12):823–30. doi:10.7556/jaoa.2020.148

114. Hogan KO, Fraga GR. Compliance With Standards for STARD 2015 Reporting Recommendations in Pathology. American Journal of Clinical Pathology. 2020 Nov 4;154(6):828–36. doi:10.1093/ajcp/aqaa103

115. Prager R, Bowdridge J, Kareemi H, Wright C, McGrath TA, McInnes MDF. Adherence to the Standards for Reporting of Diagnostic Accuracy (STARD) 2015 Guidelines in Acute Point-of-Care Ultrasound Research. JAMA Netw Open. 2020 May 1;3(5):e203871. doi:10.1001/jamanetworkopen.2020.3871

116. Andaur Navarro CL, Damen JAA, Takada T, Nijman SWJ, Dhiman P, Ma J, et al. Completeness of reporting of clinical prediction models developed using supervised machine learning: a systematic review. BMC Med Res Methodol. 2022 Jan 13;22(1):12. doi:10.1186/s12874-021-01469-6 PubMed PMID: 35026997; PubMed Central PMCID: PMC8759172.

117. Kazi S, Frank RA, Salameh J, Fabiano N, Absi M, Pozdnyakov A, et al. Evaluating the Impact of Peer Review on the Completeness of Reporting in Imaging Diagnostic Test Accuracy Research. Magnetic Resonance Imaging. 2022 Sep;56(3):680–90. doi:10.1002/jmri.28116

118. Lu JH, Callahan A, Patel BS, Morse KE, Dash D, Pfeffer MA, et al. Assessment of Adherence to Reporting Guidelines by Commonly Used Clinical Prediction Models From a Single Vendor: A Systematic Review. JAMA Network Open. 2022;5(8). Located at: ProQuest Central; 2736878028. doi:10.1001/jamanetworkopen.2022.27779

119. Zhou J, Li J, Zhang J, Geng B, Chen Y, Zhou X. The relationship between endorsing reporting guidelines or trial registration and the impact factor or total citations in surgical journals. PeerJ. 2022 Jan 25;10:e12837. doi:10.7717/peerj.12837

120. Andaur Navarro CL, Damen JAA, Takada T, Nijman SWJ, Dhiman P, Ma J, et al. Systematic review finds “spin” practices and poor reporting standards in studies on machine learning-based prediction models. J Clin Epidemiol. 2023 Jun;158:99–110. doi:10.1016/j.jclinepi.2023.03.024 PubMed PMID: 37024020.

121. Gorrepati PL, Smith GP. Evaluating dermatology journals’ use of reporting guidelines in “Author Guidelines.” Arch Dermatol Res. 2022 Nov 19. doi:10.1007/s00403-022-02427-y

122. Ozdag Y, Hayes DS, Callahan C, El Koussaify J, Warnick EP, Foster BK, et al. Adherence to Complication Reporting for Randomized Controlled Trials Contained in Clinical Practice Guidelines for the Management of Carpal Tunnel Syndrome. Journal of Hand Surgery Global Online. 2023 Nov;5(6):779–83. doi:10.1016/j.jhsg.2023.06.004

123. Zhong J, Xing Y, Lu J, Zhang G, Mao S, Chen H, et al. The endorsement of general and artificial intelligence reporting guidelines in radiological journals: a meta-research study. BMC Med Res Methodol. 2023 Dec 13;23(1):292. doi:10.1186/s12874-023-02117-x

124. Kashif Al-Ghita M, Dawit H, Kazi S, Adamo RG, Islam N, Karpinski S, et al. Evaluation of Imaging Research Adherence to the STARD 2015 Reporting Guideline: Update 9 Years After Implementation and Baseline Assessment. Can Assoc Radiol J. 2025 Nov;76(4):631–45. doi:10.1177/08465371251324090

125. Khalid SI, Roy JM, Massaad E, Thomson K, Mirpuri P, Patel A, et al. An Appraisal of the Quality of Development and Reporting of Predictive Models in Spine Surgery. Global Spine Journal. 2025 Sep;15(7):3457–64. doi:10.1177/21925682251335880

126. Knez N, Kroflin K, Fraga GR. Publications on the diagnostic accuracy of dermatopathology tests: A cross‐sectional quality analysis. J Cutan Pathol. 2023 Nov;50(11):1020–6. doi:10.1111/cup.14504

127. Kocak B, Akinci D’Antonoli T, Ates Kus E, Keles A, Kala A, Kose F, et al. Self-reported checklists and quality scoring tools in radiomics: a meta-research. Eur Radiol. 2024 Jan 5;34(8):5028–40. doi:10.1007/s00330-023-10487-5

128. Uribe SE, Hamdan MH, Valente NA, Yamaguchi S, Umer F, Tichy A, et al. Evaluating dental AI research papers: Key considerations for editors and reviewers. Journal of Dentistry. 2025 Sep;160:105867. doi:10.1016/j.jdent.2025.105867

129. Ko JS, Heo H, Suh CH, Yi J, Shim WH. Adherence of Studies on Large Language Models for Medical Applications Published in Leading Medical Journals According to the MI-CLEAR-LLM Checklist. Korean J Radiol. 2025 Apr;26(4):304–12. doi:10.3348/kjr.2024.1161 PubMed PMID: 40015560; PubMed Central PMCID: PMC11955383.

130. Koçak B, Köse F, Keleş A, Şendur A, Meşe İ, Karagülle M. Adherence to the Checklist for Artificial Intelligence in Medical Imaging (CLAIM): an umbrella review with a comprehensive two-level analysis. dir. 2025 Feb 10. doi:10.4274/dir.2025.243182

131. Yang S, Han L. Machine learning-driven risk stratification for distant metastasis in gastric cancer: A comparative study of clinical features and composite indices integrated models. PLoS One. 2025 Oct;20(10):19. Located at: ProQuest Central; 3267302919. doi:10.1371/journal.pone.0335258

132. Animal Welfare and Ethics. Altern Lab Anim. 1996 Oct;24(1_suppl):65–180. doi:10.1177/026119299602401s14

133. XVIIIth International Symposium on Cerebral Blood Flow and Metabolism. J Cereb Blood Flow Metab. 1997 Jun;17(1_suppl):S1–806. doi:10.1177/0271678X9701701s01

134. British Society for Dental Research The British Division of the IADR 45th Annual Meeting April 7-10,1997 University of Sussex, Brighton, England. J Dent Res. 1997 May;76(5):1014–83. doi:10.1177/00220345970760050101

135. Gunn S, Kandola J. Structural Modelling with Sparse Kernels. Machine Learning. 2002 Jul;48(1–3):137–63. Located at: ProQuest Central; 758924950. doi:10.1023/A:1013903804720

136. Oral Presentations. Mult Scler. 2005 Sep;11(5_suppl):S1–182. doi:10.1191/1352458505ms1258xx

137. Hall S, Lewith G, Brien S, Little P. A Review of the Literature in Applied and Specialised Kinesiology. Forsch Komplementärmed. 2008;15(1):40–6. doi:10.1159/000112820

138. The 9th International Congress on SLE June 24 – 27 2010, Vancouver, Canada. Lupus. 2010 Jun;19(1_suppl):1–185. doi:10.1177/09612033100190010101

139. Oral Presentations. Mult Scler. 2010 Oct;16(10_suppl):7–366. doi:10.1177/1352458510383204

140. Poster I. Mult Scler. 2012 Oct;18(4_suppl):55–277. doi:10.1177/1352458512459019

141. The 10th International Congress on SLE April 18–21 2013, Buenos Aires, Argentina Oral Presentations. Lupus. 2013 Mar;22(1_suppl):1–196. doi:10.1177/0961203313476777

142. UEG Week 2013 Poster Presentations. United European Gastroenterology Journal. 2013 Oct;1(1_suppl):A135–587. doi:10.1177/2050640613502900

143. ECTRIMS 2013: Posters I. Mult Scler. 2013 Oct;19(11_suppl):74–558. doi:10.1177/1352458513502429

144. Chapman R.W., Reading B.J., Sullivan C.V. Ovary transcriptome profiling via artificial intelligence reveals a transcriptomic fingerprint predicting egg quality in striped bass, Morone saxatilis. PLoS ONE. 2014;9(5):e96818. Located at: Ovid Emcare <2010 to 2014>. doi:10.1371/journal.pone.0096818

145. UEG Week 2014 Poster Presentations. United European Gastroenterology Journal. 2014 Oct;2(1_suppl):A132–605. doi:10.1177/2050640614548980

146. Poster Session 1. Mult Scler. 2015 Sep;21(11_suppl):76–653. doi:10.1177/1352458515602642

147. UEG Week 2015 Oral Presentations. United European Gastroenterology Journal. 2015 Oct;3(5_suppl):1–145. doi:10.1177/2050640615601611

148. UEG Week 2015 Poster Presentations. United European Gastroenterology Journal. 2015 Oct;3(5_suppl):146–687. doi:10.1177/2050640615601623

149. UEG Week 2016 Poster Presentations. United European Gastroenterology Journal. 2016 Oct;4(5_suppl):A157–720. doi:10.1177/2050640616663689

150. ePosters. Mult Scler. 2016 Sep;22(3_suppl):706–827. doi:10.1177/1352458516663067

151. Poster Session 1. Mult Scler. 2016 Sep;22(3_suppl):88–399. doi:10.1177/1352458516663081

152. Poster Session 2. Mult Scler. 2016 Sep;22(3_suppl):400–705. doi:10.1177/1352458516663086

153. Oral Presentations. Mult Scler. 2016 Sep;22(3_suppl):7–87. doi:10.1177/1352458516663068

154. Poster Session 1. Mult Scler. 2017 Oct;23(3_suppl):85–426. doi:10.1177/1352458517731404

155. UEG Week 2017 Poster Presentations. United European Gastroenterology Journal. 2017 Oct;5(5_suppl):A161–836. doi:10.1177/2050640617725676

156. ePosters. Mult Scler. 2017 Oct;23(3_suppl):680–975. doi:10.1177/1352458517731285

157. Poster Session 2. Mult Scler. 2017 Oct;23(3_suppl):427–679. doi:10.1177/1352458517731406

158. UEG Week 2017 Oral Presentations. United European Gastroenterology Journal. 2017 Oct;5(5_suppl):A1–160. doi:10.1177/2050640617725668

159. E-Posters. Global Spine Journal. 2017 May;7(2_suppl):190S-373S. doi:10.1177/2192568217708189

160. Hayashi Y, Oishi T. High Accuracy-priority Rule Extraction for Reconciling Accuracy and Interpretability in Credit Scoring. New Generation Computing. 2018;36(4):393–418. Located at: ProQuest Central; 2113615696. doi:10.1007/s00354-018-0043-5

161. Evans B, Ossorio P. The Challenge of Regulating Clinical Decision Support Software After 21^st^ Century Cures. Am J Law Med. 2018 May;44(2–3):237–51. doi:10.1177/0098858818789418

162. ePosters. Mult Scler. 2018 Oct;24(2_suppl):738–980. doi:10.1177/1352458518798592

163. Oral presentations. Mult Scler. 2018 Oct;24(2_suppl):8–120. doi:10.1177/1352458518798579

164. Poster Session 1. Mult Scler. 2018 Oct;24(2_suppl):121–327. doi:10.1177/1352458518798582

165. Poster Session 2. Mult Scler. 2018 Oct;24(2_suppl):328–529. doi:10.1177/1352458518798590

166. UEG Week 2018 Poster Presentations. UEG Journal. 2018 Oct;6(S8). doi:10.1177/2050640618792819

167. 2018 Southern Regional Meeting. Journal of Investigative Medicine. 2018 Feb;66(2):354–554. doi:10.1136/jim-2017-000697.1

168. Scott JR. Reproductive immunology from the perspective of the clinician. Journal of Reproductive Immunology. 2019 Jun;133:27–9. doi:10.1016/j.jri.2019.05.002

169. UEG Week 2019 Poster Presentations. UEG Journal. 2019 Oct;7(S8):189–1030. doi:10.1177/2050640619854671

170. ECTRIMS 2019 - Poster Session 2. Mult Scler. 2019 Sep;25(2_suppl):357–580. doi:10.1177/1352458519868080

171. E-Posters. Global Spine Journal. 2019 May;9(2_suppl):188S-533S. doi:10.1177/2192568219839731

172. Bevilacqua M, Bottani E, Ciarapica FE, Costantino F, Di Donato L, Ferraro A, et al. Digital Twin Reference Model Development to Prevent Operators’ Risk in Process Plants. Sustainability. 2020 Feb 4;12(3):1088. doi:10.3390/su12031088

173. Pena HP, Belo VS, Xavier‐Junior JCC, Teixeira‐Neto RG, Melo SN, Pereira DA, et al. Accuracy of diagnostic tests for American tegumentary leishmaniasis: a systematic literature review with meta‐analyses. Tropical Med Int Health. 2020 Oct;25(10):1168–81. doi:10.1111/tmi.13465

174. **Ueg** Week 2020 Poster Presentations. UEG Journal. 2020 Oct;8(S8):144–887. doi:10.1177/2050640620927345

175. Midwest Clinical and Translational Research Meeting of Csctr and Mwafmr. Journal of Investigative Medicine. 2020 Jun;68(5):1026–121. doi:10.1136/jim-2020-MW

176. Arora SK, Kelley S, Madhavan S. Building a Sample Frame of SMEs Using Patent, Search Engine, and Website Data. Journal of Official Statistics. 2021 Mar 1;37(1):1–30. doi:10.2478/jos-2021-0001

177. Bukhsh ZA, Jansen N, Aaqib S. Damage detection using in-domain and cross-domain transfer learning. Neural Computing & Applications. 2021 Dec;33(24):16921–36. Located at: ProQuest Central; 2593746454. doi:10.1007/s00521-021-06279-x

178. Bjoersum-Meyer T, Kaalby L, Lund L, Christensen P, Jakobsen MS, Baatrup G, et al. Long-term Functional Urinary and Sexual Outcomes in Patients with Anorectal Malformations—A Systematic Review. European Urology Open Science. 2021 Mar;25:29–38. doi:10.1016/j.euros.2021.01.007

179. Halder AK. AKT Inhibitors: The Road Ahead to Computational Modeling-Guided Discovery. International Journal of Molecular Sciences. 2021;22(8):3944. Located at: ProQuest Central; 2548735578. doi:10.3390/ijms22083944

180. Kochueva O, Nikolskii K. Data Analysis and Symbolic Regression Models for Predicting CO and NOx Emissions from Gas Turbines. Computation. 2021;9(12):139. Located at: ProQuest Central; 2612753744. doi:10.3390/computation9120139

181. Consistency of ranking was evaluated as new measure for prediction model stability: longitudinal cohort study. Journal of Clinical Epidemiology. 2021 Oct;138:168–77. Located at: ProQuest Central; 2593602218. doi:10.1016/j.jclinepi.2021.06.026

182. Obeng-Gyasi E, Roostaei J, Gibson JM. Lead Distribution in Urban Soil in a Medium-Sized City: Household-Scale Analysis. Environmental Science & Technology. 2021 Mar 16;55(6):3696. Located at: ProQuest Central; 2507171442. doi:10.1021/acs.est.0c07317

183. ECTRIMS 2021 – ePoster. Mult Scler. 2021 Oct;27(2_suppl):134–740. doi:10.1177/13524585211044667

184. Czub N, Pacławski A, Szlęk J, Mendyk A. Do AutoML-Based QSAR Models Fulfill OECD Principles for Regulatory Assessment? A 5-HT1A Receptor Case. Pharmaceutics. 2022;14(7):1415. Located at: ProQuest Central; 2694055308. doi:10.3390/pharmaceutics14071415

185. Pawar PV, Paluri RA. Big Data Analytics in Logistics and Supply Chain Management: A Review of Literature. Vision: The Journal of Business Perspective. 2022 May 16;09722629221091655. doi:10.1177/09722629221091655

186. ECTRIMS 2022 – Poster. Mult Scler. 2022 Oct;28(3_suppl):130–691. doi:10.1177/13524585221123687

187. ACTRIMS Forum 2022- Poster Presentations. Mult Scler. 2022 May;28(1_suppl):20–214. doi:10.1177/13524585221094745

188. ECTRIMS 2022 – Oral Presentations. Mult Scler. 2022 Oct;28(3_suppl):3–129. doi:10.1177/13524585221123685

189. Endalie D., Haile G., Taye W. Deep learning-based idiomatic expression recognition for the Amharic language. PLoS ONE. 2023;18(12 December):e0295339. Located at: Ovid Emcare <2021 to 2025 Week 28>. doi:10.1371/journal.pone.0295339

190. Garcia Valencia OA, Thongprayoon C, Jadlowiec CC, Mao SA, Miao J, Cheungpasitporn W. Enhancing Kidney Transplant Care through the Integration of Chatbot. Healthcare. 2023;11(18):2518. Located at: ProQuest Central; 2869340033. doi:10.3390/healthcare11182518

191. Sada Del Real K., Rubio A. Discovering the mechanism of action of drugs with a sparse explainable network. eBioMedicine. 2023;95((Sada Del Real, Rubio) Departamento de Ingenieria Biomedica y Ciencias, TECNUN, Universidad de Navarra, San Sebastian 20018, Spain):104767. Located at: Ovid Emcare <2021 to 2025 Week 28>. doi:10.1016/j.ebiom.2023.104767

192. ACTRIMS Forum 2023 - Poster Presentations. Mult Scler. 2023 May;29(2_suppl):18–242. doi:10.1177/13524585231169437

193. Aldousari E, Kithinji D. Artificial intelligence and health information: A bibliometric analysis of three decades of research. Health Informatics J. 2024 Jul;30(3):14604582241283969. doi:10.1177/14604582241283969

194. Development of modelling and digitalization tools for alumina refinery. CSI Transactions on ICT. 2024;12(1):39–55. Located at: ProQuest Central; 3169149153. doi:10.1007/s40012-024-00394-5

195. Jibrin AM, Abba SI, Usman J, Al-Suwaiyan M, Aldrees A, Dan’azumi S, et al. Tracking the impact of heavy metals on human health and ecological environments in complex coastal aquifers using improved machine learning optimization. Environmental Science and Pollution Research. 2024 Aug;31(40):53219–36. Located at: ProQuest Central; 3101375457. doi:10.1007/s11356-024-34716-6

196. Nguyen V.-N., Ho T.-T., Doan T.-D., Le N.Q.K. Using a hybrid neural network architecture for DNA sequence representation: A study on N4-methylcytosine sites. Comput Biol Med. 2024;178((Nguyen) University of Information and Communication Technology, Thai Nguyen University, Thai Nguyen, Vietnam):108664. Located at: Ovid Emcare <2021 to 2025 Week 28>. doi:10.1016/j.compbiomed.2024.108664

197. Perroni MG, Veiga CPD, Forteski E, Marconatto DAB, Da Silva WV, Senff CO, et al. Integrating Relative Efficiency Models with Machine Learning Algorithms for Performance Prediction. Sage Open. 2024 Apr;14(2):21582440241257800. doi:10.1177/21582440241257800

198. Time A, Pacheco DADJ, Ionescu SC. Integrating technical and business aspects in Software as a Service: Systematic literature review, trends, and research directions. Information Development. 2024 Oct 15;02666669241287499. doi:10.1177/02666669241287499

199. 45^th^ Annual North American Meeting of the Society for Medical Decision Making, Philadelphia, Pennsylvania, United States, 22-25 October 2023. Med Decis Making. 2024 Apr;44(3):NP1–375. doi:10.1177/0272989X241236400

200. ECTRIMS 2024 Late Breaking Poster. Mult Scler. 2024 Sep;30(3_suppl):1148–211. doi:10.1177/13524585241269220

201. E-Posters. Global Spine Journal. 2024 May;14(4_suppl):363S-689S. doi:10.1177/21925682241239483

202. ECTRIMS 2024 – ePoster. Mult Scler. 2024 Sep;30(3_suppl):681–1137. doi:10.1177/13524585241269221

203. ECTRIMS 2024 – Poster. Mult Scler. 2024 Sep;30(3_suppl):125–680. doi:10.1177/13524585241269219

204. ECTRIMS 2024 – Oral Presentations. Mult Scler. 2024 Sep;30(3_suppl):4–124. doi:10.1177/13524585241269218

205. Alqaraleh M, Almagharbeh WT, Ahmad MW. Exploring the impact of artificial intelligence integration on medication error reduction: A nursing perspective. Nurse Education in Practice. 2025 Jul;86:104438. doi:10.1016/j.nepr.2025.104438

206. Barreto S. Near-Death Experiences: A Bibliometric and Systematic Review of Published Literature From 1977 to 2025. Omega (Westport). 2025 Jul 30;00302228251363788. doi:10.1177/00302228251363788

207. Bassil KC, van Dellen E. [AI in mental healthcare: hope or hype?]. Ned Tijdschr Geneeskd. 2025 May 22;169:D8469. PubMed PMID: 40391869.

208. Hanum UL, Qauli AI, Fuadah YN, Izza RN, Lim KM. Validation of new AI-based classification method for in silico cardiac safety assessment of drugs following the CiPA framework. Archives of Toxicology Archiv für Toxikologie. 2025 Sep;99(9):3735–49. Located at: ProQuest Central; 3246233725. doi:10.1007/s00204-025-04079-z

209. Kalaycıoğlu O, Pavlou M, Akhanlı SE, De Belder MA, Ambler G, Omar RZ. Evaluating the sample size requirements of tree-based ensemble machine learning techniques for clinical risk prediction. Stat Methods Med Res. 2025 Jul;34(7):1356–72. doi:10.1177/09622802251338983

210. Comparative analysis of the performance of the large language models ChatGPT-3.5, ChatGPT-4 and Open AI-o1 in the field of Programmed Cell Death in myeloma. Discover Oncology. 2025 Dec;16(1):870. Located at: ProQuest Central; 3207965280. doi:10.1007/s12672-025-02648-3

211. Calibrated explanations for regression. Machine Learning. 2025 Apr;114(4):100. Located at: ProQuest Central; 3169289715. doi:10.1007/s10994-024-06642-8

212. Marín Díaz Gabriel. Quality Management in Chemical Processes Through Fuzzy Analysis: A Fuzzy C-Means and Predictive Models Approach. ChemEngineering. 2025;9(3):45. Located at: ProQuest Central; 3223882278. doi:10.3390/chemengineering9030045

213. Mohsin SS, Salman OH, Jasim AA, Yahya MZ, Alwindawi H. A real-time web-based telemedicine framework based on AI and IoMT for emergency triage and initial diagnostics: the TeleMedQuick solution. International Journal of Medical Informatics. 2025 Dec;204:106074. doi:10.1016/j.ijmedinf.2025.106074

214. Miedema J, Lutz B, Gerber S, Kovlyagina I, Todorov H. Balancing ethics and statistics: machine learning facilitates highly accurate classification of mice according to their trait anxiety with reduced sample sizes. Translational Psychiatry. 2025;15(1):304. Located at: ProQuest Central; 3241758600. doi:10.1038/s41398-025-03546-6

215. Millán-Palacios S, Sánchez-Soriano J. Investment Portfolios Optimization with Genetic Algorithm: An Approach Applied to the Spanish Market (IBEX 35). Electronics. 2025;14(13):2559. Located at: ProQuest Central; 3229142575. doi:10.3390/electronics14132559

216. Martorell-Marugan J., Lopez-Dominguez R., Villatoro-Garcia J.A., Toro-Dominguez D., Chierici M., Jurman G., et al. Explainable deep neural networks for predicting sample phenotypes from single-cell transcriptomics. Brief Bioinform. 2025;26(1):bbae673. Located at: Ovid Emcare <2021 to 2025 Week 28>. doi:10.1093/bib/bbae673

217. Nikoloudi M, Mystakidou K. Artificial Intelligence in Palliative Care: A Scoping Review of Current Applications, Challenges, and Future Directions. Am J Hosp Palliat Care. 2025 Jul;10499091251358379. doi:10.1177/10499091251358379

218. Retraction Notice. Journal of Intelligent & Fuzzy Systems: Applications in Engineering and Technology. 2025 Apr 17;10641246251331509. doi:10.1177/10641246251331509

219. ESAO 2025 Innovations in (bio)artificial organs and organ models. Int J Artif Organs. 2025 Jul;48(7):439–544. doi:10.1177/03913988251342624

220. Vora SR, Bianchi J, Frazier‐Bowers SA, Lamani E, Akyalcin S, Kapila S. Preface to the 10th Biennial COAST Conference: AI‐ and Biomedicine‐Driven Precision Orthodontics and Craniofacial Care. Orthodontics & Craniofacial Research. 2025 Dec;28:S4–9. Located at: ProQuest Central; 3306233701. doi:10.1111/ocr.70079

221. Ramesh S. A checklist to protect human rights in artificial-intelligence research. Nature. 2017 Dec 21;552(7685):334–334. doi:10.1038/d41586-017-08875-1

222. Collins GS, Moons KGM. Reporting of artificial intelligence prediction models. The Lancet. 2019 Apr;393(10181):1577–9. doi:10.1016/S0140-6736(19)30037-6

223. Sounderajah V, Ashrafian H, Aggarwal R, De Fauw J, Denniston AK, Greaves F, et al. Developing specific reporting guidelines for diagnostic accuracy studies assessing AI interventions: The STARD-AI Steering Group. Nat Med. 2020 Jun;26(6):807–8. doi:10.1038/s41591-020-0941-1

224. Sounderajah V, Ashrafian H, Rose S, Shah NH, Ghassemi M, Golub R, et al. A quality assessment tool for artificial intelligence-centered diagnostic test accuracy studies: QUADAS-AI. Nat Med. 2021 Oct;27(10):1663–5. doi:10.1038/s41591-021-01517-0

225. Bartlett B. The possibility of AI-induced medical manslaughter: Unexplainable decisions, epistemic vices, and a new dimension of moral luck. Medical Law International. 2023 Sep;23(3):241–70. doi:10.1177/09685332231193944

226. Bacchi S, Kovoor J, Gupta A, Chan W. Should this artificial intelligence algorithm be used in my practice now? A checklist approach. Clinical Exper Ophthalmology. 2024 Jan;52(1):123–5. doi:10.1111/ceo.14307

227. Deroncele-Acosta A, Bellido-Valdiviezo O, Sánchez-Trujillo MDLÁ, Palacios-Núñez ML, Rueda-Garcés H, Brito-Garcías JG. Ten Essential Pillars in Artificial Intelligence for University Science Education: A Scoping Review. Sage Open. 2024 Jul;14(3):21582440241272016. doi:10.1177/21582440241272016

228. Ye H. Some suggestions for 'A checklist for reporting, reading and evaluating Artificial Intelligence Technology Enhanced Learning (AITEL) research in medical education’. Medical Teacher. 2024 Jun 2;46(6):859–60. doi:10.1080/0142159X.2024.2317916

229. Van Trijffel E, Anderegg Q, Bossuyt PMM, Lucas C. Inter-examiner reliability of passive assessment of intervertebral motion in the cervical and lumbar spine: A systematic review. Manual Therapy. 2005 Nov;10(4):256–69. doi:10.1016/j.math.2005.04.008

230. deSouza NM, Morgan VA, Bancroft E, Sohaib SA, Giles SL, Kote-Jarai Z, et al. Diffusion-weighted MRI for detecting prostate tumour in men at increased genetic risk. European Journal of Radiology Open. 2014;1:22–7. doi:10.1016/j.ejro.2014.08.002

231. Schwartz SA, Utts J, Spottiswoode SJP, Shade CW, Tully L, Morris WF, et al. A Double-Blind, Randomized Study to Assess the Validity of Applied Kinesiology (AK) as a Diagnostic Tool and as a Nonlocal Proximity Effect. EXPLORE. 2014 Mar;10(2):99–108. doi:10.1016/j.explore.2013.12.002

232. Van Loon K, Van Zaane B, Bosch EJ, Kalkman CJ, Peelen LM. Non-Invasive Continuous Respiratory Monitoring on General Hospital Wards: A Systematic Review. Rosenberger P, editor. PLoS ONE. 2015 Dec 14;10(12):e0144626. doi:10.1371/journal.pone.0144626

233. Ferraro PM, Lombardi G, Naticchia A, Sturniolo A, Zuppi C, De Stefano V, et al. A STARD-compliant prediction model for diagnosing thrombotic microangiopathies. J Nephrol. 2018 Jun;31(3):405–10. doi:10.1007/s40620-018-0468-4

234. Sampieri CL, Orozco-Ortega RA. Matrix metalloproteinases and tissue inhibitors of metalloproteinases in chronic kidney disease and acute kidney injury: a systematic review of the literature. Hippokratia. 2018;22(3):99–104. PubMed PMID: 31641330; PubMed Central PMCID: PMC6801125.

235. Sun R, Hsieh KL, Sosnoff JJ. Fall Risk Prediction in Multiple Sclerosis Using Postural Sway Measures: A Machine Learning Approach [Internet]. Bioengineering; 2018 [cited 2025 Nov 26]. Available from: http://biorxiv.org/lookup/doi/10.1101/410704 doi:10.1101/410704

236. Gudmundsson HT, Hansen KE, Halldorsson BV, Ludviksson BR, Gudbjornsson B. Clinical decision support system for the management of osteoporosis compared to NOGG guidelines and an osteology specialist: a validation pilot study. BMC Medical Informatics and Decision Making. 2019;19. Located at: ProQuest Central; 2183188182. doi:10.1186/s12911-019-0749-4

237. Shillan D, Sterne JAC, Champneys A, Gibbison B. Use of machine learning to analyse routinely collected intensive care unit data: a systematic review. Crit Care. 2019 Aug 22;23(1):284. doi:10.1186/s13054-019-2564-9 PubMed PMID: 31439010; PubMed Central PMCID: PMC6704673.

238. Sun R, Hsieh KL, Sosnoff JJ. Fall Risk Prediction in Multiple Sclerosis Using Postural Sway Measures: A Machine Learning Approach. Scientific Reports (Nature Publisher Group). 2019 Nov;9:1–7. Located at: ProQuest Central; 2312546989. doi:10.1038/s41598-019-52697-2

239. Anderson AB, Grazal CF, Balazs GC, Potter BK, Dickens JF, Forsberg JA. Can Predictive Modeling Tools Identify Patients at High Risk of Prolonged Opioid Use After ACL Reconstruction? Clinical Orthopaedics and Related Research. 2020 Jul;478(7). Located at: ProQuest Central; 2459613056. doi:10.1097/CORR.0000000000001251

240. Baldwin DR, Gustafson J, Pickup L, Arteta C, Novotny P, Declerck J, et al. External validation of a convolutional neural network artificial intelligence tool to predict malignancy in pulmonary nodules. Thorax. 2020 Apr;75(4):306–12. Located at: ProQuest Central; 2383731149. doi:10.1136/thoraxjnl-2019-214104

241. Cresswell K, Callaghan M, Khan S, Sheikh Z, Mozaffar H, Sheikh A. Investigating the use of data-driven artificial intelligence in computerised decision support systems for health and social care: A systematic review. Health Informatics J. 2020 Sep;26(3):2138–47. doi:10.1177/1460458219900452 PubMed PMID: 31964204.

242. Low DM, Bentley KH, Ghosh SS. Automated assessment of psychiatric disorders using speech: A systematic review. Laryngoscope Investigative Otolaryngology. 2020 Feb;5(1):96–116. Located at: ProQuest Central; 2363674592. doi:10.1002/lio2.354

243. Medina MM, Polo R, Amilibia E, Roca-Ribas F, Díaz M, Pérez M, et al. Diagnostic Accuracy of Intracochlear Test Electrode for Acoustic Nerve Monitoring in Vestibular Schwannoma Surgery. Ear & Hearing. 2020 Nov;41(6):1648–59. doi:10.1097/AUD.0000000000000883

244. Miles J, Turner J, Jacques R, Williams J, Mason S. Using machine-learning risk prediction models to triage the acuity of undifferentiated patients entering the emergency care system: a systematic review. Diagnostic and Prognostic Research. 2020;4:1–12. Located at: ProQuest Central; 2546774053. doi:10.1186/s41512-020-00084-1

245. Wongvibulsin S, Wu KC, Zeger SL. Improving Clinical Translation of Machine Learning Approaches Through Clinician-Tailored Visual Displays of Black Box Algorithms: Development and Validation. JMIR Med Inform. 2020 Jun 9;8(6):e15791. doi:10.2196/15791

246. Alim-Marvasti A, Pérez-García F, Dahele K, Romagnoli G, Diehl B, Sparks R, et al. Machine Learning for Localizing Epileptogenic-Zone in the Temporal Lobe: Quantifying the Value of Multimodal Clinical-Semiology and Imaging Concordance. Front Digit Health. 2021 Feb 10;3:559103. doi:10.3389/fdgth.2021.559103

247. Ferreira-Santos D, Rodrigues PP. Enhancing Obstructive Sleep Apnea Diagnosis With Screening Through Disease Phenotypes: Algorithm Development and Validation. JMIR Med Inform. 2021 Jun 22;9(6):e25124. doi:10.2196/25124

248. Hamidi F., Gilani N., Belaghi R.A., Sarbakhsh P., Edgunlu T., Santaguida P. Exploration of Potential miRNA Biomarkers and Prediction for Ovarian Cancer Using Artificial Intelligence. Front Genet. 2021;12((Hamidi, Gilani, Sarbakhsh) Department of Statistics and Epidemiology, Faculty of Health, Tabriz University of Medical Sciences, Tabriz, Iran, Islamic Republic of):724785. Located at: Ovid Emcare <2021 to 2025 Week 28>. doi:10.3389/fgene.2021.724785

249. Ibrahim W, Natarajan S, Wilde M, Cordell R, Monks PS, Greening N, et al. A systematic review of the diagnostic accuracy of volatile organic compounds in airway diseases and their relation to markers of type-2 inflammation. ERJ Open Res. 2021 Jul;7(3):00030–2021. doi:10.1183/23120541.00030-2021

250. Kareemi H, Vaillancourt C, Rosenberg H, Fournier K, Yadav K. Machine Learning Versus Usual Care for Diagnostic and Prognostic Prediction in the Emergency Department: A Systematic Review. Acad Emerg Med. 2021 Feb;28(2):184–96. doi:10.1111/acem.14190 PubMed PMID: 33277724.

251. Mao-feng W, Fei-xiang L, Lan-fang F, Chao-nan Z, Shuang-yan F, Cai-min S, et al. Development and validation of a novel risk assessment model to estimate the probability of pulmonary embolism in postoperative patients. Scientific Reports (Nature Publisher Group). 2021;11(1). Located at: ProQuest Central; 2571040542. doi:10.1038/s41598-021-97638-0

252. Rivera SC, Liu X, Chan AW, Denniston AK, Calvert MJ. Guidelines for clinical trial protocols for interventions involving artificial intelligence: the SPIRIT-AI Extension. BMJ. 2020 Sep 9;m3210. doi:10.1136/bmj.m3210

253. Møller JK, Sørensen M, Hardahl C. Prediction of risk of acquiring urinary tract infection during hospital stay based on machine-learning: A retrospective cohort study. PLoS One. 2021 Mar;16(3). Located at: ProQuest Central; 2507667303. doi:10.1371/journal.pone.0248636

254. Knoop J, W. van Lankveld, Beijer L, Geerdink FJB, Heymans MW, Hoogeboom TJ, et al. Development and internal validation of a machine learning prediction model for low back pain non-recovery in patients with an acute episode consulting a physiotherapist in primary care. BMC Musculoskeletal Disorders. 2022;23:1–14. Located at: ProQuest Central; 2715389184. doi:10.1186/s12891-022-05718-7

255. Labiste CC, McElroy E, Subhawong TK, Banks JS. Systematic review: investigating the added diagnostic value of gadolinium contrast agents for osteomyelitis in the appendicular skeleton. Skeletal Radiol. 2022 Jun;51(6):1285–96. doi:10.1007/s00256-021-03915-4

256. O’Connor MK, Dai H, Fraga GR. PRAME immunohistochemistry for melanoma diagnosis: A STARD ‐compliant diagnostic accuracy study. J Cutan Pathol. 2022 Sep;49(9):780–6. doi:10.1111/cup.14267

257. Vasey B, Novak A, Ather S, Ibrahim M, McCulloch P. DECIDE-AI: a new reporting guideline and its relevance to artificial intelligence studies in radiology. Clin Radiol. 2023 Feb;78(2):130–6. doi:10.1016/j.crad.2022.09.131 PubMed PMID: 36639172.

258. Xu X, Yu Z, Ge Z, Chow EPF, Bao Y, Ong JJ, et al. Web-Based Risk Prediction Tool for an Individual’s Risk of HIV and Sexually Transmitted Infections Using Machine Learning Algorithms: Development and External Validation Study. Journal of Medical Internet Research. 2022 Aug. Located at: Coronavirus Research Database; ProQuest Central; 2708674557. doi:10.2196/37850

259. Abdulazeem H, Whitelaw S, Schauberger G, Klug SJ. A systematic review of clinical health conditions predicted by machine learning diagnostic and prognostic models trained or validated using real-world primary health care data. PLoS One. 2023 Sep;18(9). Located at: ProQuest Central; 2862748910. doi:10.1371/journal.pone.0274276

260. Ajuwon BI, Awotundun ON, Richardson A, Roper K, Sheel M, Rahman N, et al. Machine learning prediction models for clinical management of blood-borne viral infections: a systematic review of current applications and future impact. International Journal of Medical Informatics. 2023 Nov;179:105244. doi:10.1016/j.ijmedinf.2023.105244

261. Alfieri F, Ancona A, Tripepi G, Rubeis A, Arjoldi N, Finazzi S, et al. Continuous and early prediction of future moderate and severe Acute Kidney Injury in critically ill patients: Development and multi-centric, multi-national external validation of a machine-learning model. PLoS One. 2023 Jul;18(7). Located at: Coronavirus Research Database; ProQuest Central; 2841858669. doi:10.1371/journal.pone.0287398

262. Chan PZ, Ramli MAIB, Chew HSJ. Diagnostic Test Accuracy of artificial intelligence-assisted detection of acute coronary syndrome: A systematic review and meta-analysis. Computers in Biology and Medicine. 2023 Dec;167:107636. doi:10.1016/j.compbiomed.2023.107636

263. da Silva AP, dos Santos HDP, Rotta ALO, Baiocco GG, Vieira R, Urbanetto J de S. Drug-related fall risk in hospitals: a machine learning approach. Acta Paulista de Enfermagem. 2023;36:1-7,7A-7G. Located at: ProQuest Central; 3165450177. doi:10.37689/acta-ape/2023AO007711

264. Dosis A, Helliwell J, Syversen A, Tiernan J, Zhiqiang Z, Jayne D. O085 Estimating postoperative mortality in colorectal surgery- a systematic review of risk prediction models. British Journal of Surgery, suppl 3. 2023 May;110. Located at: ProQuest Central; 3240882773. doi:10.1093/bjs/znad101.085

265. Doudesis D, Lee KK, Boeddinghaus J, Bularga A, Ferry AV, Tuck C, et al. Machine learning for diagnosis of myocardial infarction using cardiac troponin concentrations. Nature Medicine. 2023 May;29(5):1201–10. Located at: ProQuest Central; 2817275061. doi:10.1038/s41591-023-02325-4

266. Fanni S.C., Romei C., Ferrando G., Volpi F., D’Amore C.A., Bedini C., et al. Natural language processing to convert unstructured COVID-19 chest-CT reports into structured reports. Eur J Radiol Open. 2023;11((Fanni, Volpi, D’Amore, Neri) Department of Translational Research, Academic Radiology, University of Pisa, Pisa, Italy):100512. Located at: Ovid Emcare <2021 to 2025 Week 28>. doi:10.1016/j.ejro.2023.100512

267. Gedefaw L, Liu CF, Ip RKL, Tse HF, Yeung MHY, Yip SP, et al. Artificial Intelligence-Assisted Diagnostic Cytology and Genomic Testing for Hematologic Disorders. Cells. 2023 Jun 30;12(13). doi:10.3390/cells12131755 PubMed PMID: 37443789; PubMed Central PMCID: PMC10340428.

268. Gokhale S, Taylor D, Gill J, Hu Y, Zeps N, Lequertier V, et al. Hospital length of stay prediction for general surgery and total knee arthroplasty admissions: Systematic review and meta-analysis of published prediction models. Digital Health. 2023 Jan;9. Located at: ProQuest Central; 2926456882. doi:10.1177/20552076231177497

269. Ishii E, Nawa N, Hashimoto S, Shigemitsu H, Fujiwara T. Development, validation, and feature extraction of a deep learning model predicting in-hospital mortality using Japan’s largest national ICU database: a validation framework for transparent clinical Artificial Intelligence (cAI) development. Anaesth Crit Care Pain Med. 2023 Apr;42(2):101167. doi:10.1016/j.accpm.2022.101167 PubMed PMID: 36302489.

270. Jiang D, Song Z, Liu P, Wang Z, Zhao R. A prediction model for severe hematological toxicity of BTK inhibitors. Ann Hematol. 2023 Oct;102(10):2765–77. doi:10.1007/s00277-023-05371-7 PubMed PMID: 37491631.

271. Smith LA, Oakden-Rayner L, Bird A, Zeng M, To MS, Mukherjee S, et al. Machine learning and deep learning predictive models for long-term prognosis in patients with chronic obstructive pulmonary disease: a systematic review and meta-analysis. Lancet Digit Health. 2023 Dec;5(12):e872–81. doi:10.1016/S2589-7500(23)00177-2 PubMed PMID: 38000872.

272. Wang J, Qiu J, Zhu T, Zeng Y, Yang H, Shang Y, et al. Prediction of Suicidal Behaviors in the Middle-aged Population: Machine Learning Analyses of UK Biobank. JMIR Public Health and Surveillance. 2023;9. Located at: ProQuest Central; 2917612504. doi:10.2196/43419

273. Zhou Y, Yang X, Ma S, Yuan Y, Yan M. A systematic review of predictive models for hospital-acquired pressure injury using machine learning. Nursing Open. 2023 Mar;10(3):1234–46. Located at: ProQuest Central; 2774800209. doi:10.1002/nop2.1429

274. Al-Dhubaibi MS, Bahaj SS, Noman A, Alkasser WY, AbdElneam AI, Mohammed GF, et al. “High specificity of PCR in diagnosing mucocutaneous leshminiasis: a systematic review and meta analysis.” BMC Infect Dis. 2024 Dec 28;24(1):1476. doi:10.1186/s12879-024-10349-5

275. Chen H, Yu D, Zhang J, Li J. Machine Learning for Prediction of Postoperative Delirium in Adult Patients: A Systematic Review and Meta-analysis. Clin Ther. 2024 Dec;46(12):1069–81. doi:10.1016/j.clinthera.2024.09.013 PubMed PMID: 39395856.

276. Cascella M., Cutugno F., Mariani F., Vitale V.N., Iuorio M., Cuomo A., et al. AI-based cancer pain assessment through speech emotion recognition and video facial expressions classification. Signa Vitae. 2024;20(12):28 EP – 38. Located at: Ovid Emcare <2021 to 2025 Week 28>. doi:10.22514/sv.2024.153

277. Jawadi Z, He R, Srivastava PK, Fonarow GC, Khalil SO, Krishnan S, et al. Predicting in‐hospital mortality among patients admitted with a diagnosis of heart failure: a machine learning approach. ESC Heart Failure. 2024 Oct 1;11(5):2490–8. Located at: ProQuest Central; 3109515825. doi:10.1002/ehf2.14796

278. Ke JXC, DhakshinaMurthy A, George RB, Branco P. The effect of resampling techniques on the performances of machine learning clinical risk prediction models in the setting of severe class imbalance: development and internal validation in a retrospective cohort. Discov Artif Intell. 2024 Nov 26;4(1):91. doi:10.1007/s44163-024-00199-0

279. Lampe D, Grosser J, Grothe D, Aufenberg B, Gensorowsky D, Witte J, et al. How intervention studies measure the effectiveness of medication safety-related clinical decision support systems in primary and long-term care: a systematic review. BMC Medical Informatics and Decision Making. 2024;24:1–18. Located at: ProQuest Central; 3079155148. doi:10.1186/s12911-024-02596-y

280. Li H, Liu Z, Sun W, Li T, Dong X. Interpretable machine learning for the prediction of death risk in patients with acute diquat poisoning. Scientific Reports (Nature Publisher Group). 2024;14(1):16101. Located at: ProQuest Central; 3079613081. doi:10.1038/s41598-024-67257-6

281. Li Y, Scheel-Sailer A, Riener R, Paez-Granados D. Mixed-variable graphical modeling framework towards risk prediction of hospital-acquired pressure injury in spinal cord injury individuals. Scientific Reports (Nature Publisher Group). 2024;14(1):25067. Located at: ProQuest Central; 3119848700. doi:10.1038/s41598-024-75691-9

282. Marinkovic M, Stojanovic-Rundic S, Stanojevic A, Tomasevic A, Jankovic R, Zoidakis J, et al. Performance and Dimensionality of Pretreatment MRI Radiomics in Rectal Carcinoma Chemoradiotherapy Prediction. Journal of Clinical Medicine. 2024;13(2):421. Located at: ProQuest Central; 2918773456. doi:10.3390/jcm13020421

283. Mäenpää SM, Korja M. Diagnostic test accuracy of externally validated convolutional neural network (CNN) artificial intelligence (AI) models for emergency head CT scans – A systematic review. International Journal of Medical Informatics. 2024 Sep;189:105523. doi:10.1016/j.ijmedinf.2024.105523

284. Mendoza-Pinto C, Sánchez-Tecuatl M, Berra-Romani R, Maya-Castro ID, Etchegaray-Morales I, Munguía-Realpozo P, et al. Machine learning in the prediction of treatment response in rheumatoid arthritis: A systematic review. Semin Arthritis Rheum. 2024 Oct;68:152501. doi:10.1016/j.semarthrit.2024.152501 PubMed PMID: 39226650.

285. Mohammadi S, Salehi MA, Jahanshahi A, Shahrabi Farahani M, Zakavi SS, Behrouzieh S, et al. Artificial intelligence in osteoarthritis detection: A systematic review and meta-analysis. Osteoarthritis Cartilage. 2024 Mar;32(3):241–53. doi:10.1016/j.joca.2023.09.011 PubMed PMID: 37863421.

286. Restini FCF, Torfeh T, Aouadi S, Hammoud R, Al-Hammadi N, Starling MTM, et al. AI tool for predicting MGMT methylation in glioblastoma for clinical decision support in resource limited settings. Scientific Reports (Nature Publisher Group). 2024;14(1):27995. Located at: ProQuest Central; 3128469215. doi:10.1038/s41598-024-78189-6

287. Ruiz-Tarrazo X, Escalona-Marfil C, Pla-Campas G, Coda A. Validity and reliability of ultrasonographic assessment of femoral and tibial torsion in children and adolescents: a systematic review. Eur J Pediatr. 2024 Jun 3;183(8):3159–71. doi:10.1007/s00431-024-05619-y

288. Shujaat S, Alfadley A, Morgan N, Jamleh A, Riaz M, Aboalela AA, et al. Emergence of artificial intelligence for automating cone-beam computed tomography-derived maxillary sinus imaging tasks. A systematic review. Clin Implant Dent Relat Res. 2024 Oct;26(5):899–912. doi:10.1111/cid.13352 PubMed PMID: 38863306.

289. Tariq R, Malik S, Khanna S. A180 SYSTEMATIC REVIEW OF MACHINE LEARNING-BASED PREDICTIVE MODELS FOR CLOSTRIDIOIDES DIFFICILE INFECTION. Journal of the Canadian Association of Gastroenterology, suppl 1. 2024 Mar;7:141–2. Located at: ProQuest Central; 3168660753. doi:10.1093/jcag/gwad061.180

290. Vueghs C, Shakeri H, Renton T, Van der Cruyssen F. Development and Evaluation of a GPT4-Based Orofacial Pain Clinical Decision Support System. Diagnostics. 2024;14(24):2835. Located at: ProQuest Central; 3149568210. doi:10.3390/diagnostics14242835

291. Abdelmoteleb S, Ghallab M, IsHak WW. Evaluating the ability of artificial intelligence to predict suicide: A systematic review of reviews. Journal of Affective Disorders. 2025 Aug;382:525–39. doi:10.1016/j.jad.2025.04.078

292. Antunes M.E., Araujo T.G., Till T.M., Pantaleao E., Mancera P.F.A., Oliveira M.H.D. Machine learning models for predicting prostate cancer recurrence and identifying potential molecular biomarkers. Front Oncol. 2025;15((Antunes) Graduate Program in Biometrics, Instituto de Biociencias de Botucatu (IBB), Universidade Estadual Paulista (UNESP), Sao Paulo, Botucatu, Brazil):1535091. Located at: Ovid Emcare <2021 to 2025 Week 28>. doi:10.3389/fonc.2025.1535091

293. Al-Husaini N, Razali R, Al-Haidose A, Al-Hamdani M, Abdallah AM. Characterizing low femoral neck BMD in Qatar Biobank participants using machine learning models. BMC Musculoskeletal Disorders. 2025;26:1–9. Located at: ProQuest Central; 3216562836. doi:10.1186/s12891-025-08726-5

294. Popat Apurva, Yadav Sweta, Obholz Jacob, Hwang EA, Rehman AU, Sharma P. The Efficacy of Artificial Intelligence in the Detection and Management of Atrial Fibrillation. Cureus. 2025;17(1). Located at: ProQuest Central; 3203886276. doi:10.7759/cureus.77135

295. Frenkel Ari, Rendon A, Chavez-Lencinas C, De la Torre Juan Carlos Gomez, MacDermott Jen, Gross Collen, et al. Internal Validation of a Machine Learning-Based CDSS for Antimicrobial Stewardship. Life. 2025;15(7):1123. Located at: Coronavirus Research Database; ProQuest Central; 3233228319. doi:10.3390/life15071123

296. Ashfaq MT, Javaid N, Alrajeh N, Ali SS. An explainable AI based new deep learning solution for efficient heart disease prediction at early stages. Evolving Systems. 2025 Feb;16(1):33. doi:10.1007/s12530-025-09664-2

297. Bouktif S., Khanday A.M.U.D., Ouni A. Explainable Predictive Model for Suicidal Ideation During COVID-19: Social Media Discourse Study. J Med Internet Res. 2025;27((Bouktif, Khanday) Department of Computer Science and Software Engineering, College of Information Technology, United Arab Emirates University, Abu Dhabi, Al Ain, United Arab Emirates):e65434. Located at: Ovid Emcare <2021 to 2025 Week 28>. doi:10.2196/65434

298. Chen Q, Zhang J, Cao B, Hu Y, Kong Y, Li B, et al. Prediction models for treatment response in migraine: a systematic review and meta-analysis. J Headache Pain. 2025 Feb 12;26(1):32. doi:10.1186/s10194-025-01972-x PubMed PMID: 39939885; PubMed Central PMCID: PMC11817351.

299. Bayor AA, Li J, Yang IA, Varnfield M. Correction: Designing Clinical Decision Support Systems (CDSS)—A User-Centered Lens of the Design Characteristics, Challenges, and Implications: Systematic Review. J Med Internet Res. 2025 Sep 25;27:e84380–e84380. doi:10.2196/84380

300. Amannah Constance, Attai Kingsley Friday, Faith-Michael U. A Data-Driven Intelligent Methodology for Developing Explainable Diagnostic Model for Febrile Diseases. Algorithms. 2025;18(4):190. Located at: ProQuest Central; 3194485343. doi:10.3390/a18040190

301. Ramwala OA, Lowry KP, Hippe DS, Unrath MPN, Nyflot MJ, Mooney SD, et al. ClinValAI: A framework for developing Cloud-based infrastructures for the External Clinical Validation of AI in Medical Imaging. In: Biocomputing 2025 [Internet]. Kohala Coast, Hawaii, USA: WORLD SCIENTIFIC; 2024 [cited 2025 Sep 25]. p. 215–28. Available from: https://www.worldscientific.com/doi/10.1142/9789819807024_0016 doi:10.1142/9789819807024_0016

302. Da Silva-Filho JE, Sousa ZDS, Caracas-de-Araújo AP, Fornagero LDS, Machado MP, De Aguiar AWO, et al. Deep learning for detecting periapical bone rarefaction in panoramic radiographs: a systematic review and critical assessment. Dentomaxillofacial Radiology. 2025 Sep 1;54(6):405–19. doi:10.1093/dmfr/twaf044

303. Deng L, Wang S, Wan D, Zhang Q, Shen W, Liu X, et al. Relative Fat Mass and Physical Indices as Predictors of Gallstone Formation: Insights From Machine Learning and Logistic Regression. International Journal of General Medicine. 2025;18:509–27. Located at: ProQuest Central; 3167163840. doi:10.2147/IJGM.S507013

304. Espinoza-Vinces C, Martínez MC, Atorrasagasti-Villar A, Rodríguez MDMG, Ezpeleta D, Irimia P. Artificial intelligence in headache medicine: between automation and the doctor-patient relationship. A systematic review. J Headache Pain. 2025 Sep 2;26(1):192. doi:10.1186/s10194-025-02143-8

305. Fass OZ, Pandolfino JE, Schauer JM, Ganesh N, Farina DA, Lat A, et al. Diagnostic Accuracy of Timed Barium Esophagram for Achalasia. Gastroenterology. 2025 Jul;169(1):63–72. doi:10.1053/j.gastro.2025.02.013

306. Diagnostic Performance of a Computer-aided System for Tuberculosis Screening in Two Philippine Cities. Acta Med Philipp. 2024. doi:10.47895/amp.vi0.8950

307. Fu Y, Huang Z, Deng X, Xu L, Liu Y, Zhang M, et al. Artificial Intelligence in Lymphoma Histopathology: Systematic Review. J Med Internet Res. 2025 Feb 14;27:e62851. doi:10.2196/62851

308. Gao C, Wu L, Wu W, Huang Y, Wang X, Sun Z, et al. Deep learning in pulmonary nodule detection and segmentation: a systematic review. Eur Radiol. 2025 Jan;35(1):255–66. doi:10.1007/s00330-024-10907-0 PubMed PMID: 38985185; PubMed Central PMCID: PMC11632000.

309. Comparing machine learning models for predicting preoperative DVT incidence in elderly hypertensive patients with hip fractures: a retrospective analysis. Scientific Reports (Nature Publisher Group). 2025;15(1):13206. Located at: ProQuest Central; 3190960696. doi:10.1038/s41598-025-97880-w

310. Haghighat S, Joghatayi M, Issa J, Azimian S, Brinz J, Ashkan A, et al. Diagnostic accuracy of artificial intelligence for obstructive sleep apnea detection: a systematic review. BMC Med Inform Decis Mak. 2025 Jul 28;25(1):278. doi:10.1186/s12911-025-03129-x PubMed PMID: 40722158; PubMed Central PMCID: PMC12306116.

311. Huang H, Wu Y, Ye H, Li J, Chen L, Huang X. Risk prediction models for diabetic retinopathy: a systematic review. Front Endocrinol (Lausanne). 2025;16:1556049. doi:10.3389/fendo.2025.1556049 PubMed PMID: 40717809; PubMed Central PMCID: PMC12291684.

312. Jemimah S., Abuhantash F., AlShehhi A. c-Triadem: A constrained, explainable deep learning model to identify novel biomarkers in Alzheimer’s disease. PLoS ONE. 2025;20(4 April):e0320360. Located at: Ovid Emcare <2021 to 2025 Week 28>. doi:10.1371/journal.pone.0320360

313. Jia J, Jin Z, Turhon M, Lin Y, Yang X, Wang Y, et al. Risk Factors and Predictive Model for Ischemic Complications in Endovascular Treatment of Intracranial Aneurysms: Insights From a Large Patient Cohort. Aging Medicine. 2025 Apr 1;8(2):126–36. Located at: ProQuest Central; 3202296459. doi:10.1002/agm2.70021

314. Khaja S, Baijoo K, Aziz R. Artificial intelligence-powered advancements in atrial fibrillation diagnostics: a systematic review. Egypt Heart J. 2025 Jul 23;77(1):73. doi:10.1186/s43044-025-00670-y

315. Khan Z, Gaidhane AM, Singh M, Ganesan S, Kaur M, Sharma GC, et al. Diagnostic Accuracy of IDX-DR for Detecting Diabetic Retinopathy: A Systematic Review and Meta-Analysis. Am J Ophthalmol. 2025 May;273:192–204. doi:10.1016/j.ajo.2025.02.022 PubMed PMID: 39986640.

316. Kuang-Ming Kuo, Chao Sheng Chang. A meta-analysis of the diagnostic test accuracy of artificial intelligence predicting emergency department dispositions. BMC Medical Informatics and Decision Making. 2025;25:1–25. Located at: Coronavirus Research Database; ProQuest Central; 3216557931. doi:10.1186/s12911-025-03010-x

317. Leitão BN, Veríssimo André, Carvalho AM, Vinga Susana. Enhancing Prognostic Signatures in Glioblastoma with Feature Selection and Regularised Cox Regression. Genes. 2025;16(5):473. Located at: ProQuest Central; 3211971210. doi:10.3390/genes16050473

318. Liu W, Ji K, Tang Q, Xia W, Zhang W, Shao L, et al. Development and validation of interpretable machine learning models for predicting AKI risk in patients treated with PD-1/PD-L1: a retrospective study. BMC Medical Informatics and Decision Making. 2025;25:1–13. Located at: ProQuest Central; 3247097977. doi:10.1186/s12911-025-03142-0

319. Medani M, Elhessewi GMS, Alqahtani M, Asklany SA, Alamro S, Albalawneh D, et al. Leveraging explainable artificial intelligence with ensemble of deep learning model for dementia prediction to enhance clinical decision support systems. Sci Rep. 2025 May 13;15(1):16639. doi:10.1038/s41598-025-97102-3 PubMed PMID: 40360623; PubMed Central PMCID: PMC12075694.

320. Meiklejohn K, Junges L, Terry JR, Whight A, Shankar R, Woldman W. Network-based biomarkers in background electroencephalography in childhood epilepsies—A scoping review and narrative synthesis. Seizure: European Journal of Epilepsy. 2025 Jan;124:89–106. doi:10.1016/j.seizure.2024.11.011

321. Mohamed Ahmed HM, Babiker Ahmed Ahmed Umballi, Siddig Mohammed Mohammed Awad, Ibrahim Omer Nasereldeen Omer, Altom DS, Elnour Mohey Aldien A. The Role of Artificial Intelligence in the Prediction of Bariatric Surgery Complications: A Systematic Review. Cureus. 2025;17(4). Located at: ProQuest Central; 3214251833. doi:10.7759/cureus.82461

322. Mohamed Dkeen Nagla Osman, Dawelbait Radwan Madina Eltayeb, Alnaw Zumam Israa Ali, Abd Elfrag Mohamed Nihal Ahmed, Abbashar Abdelmahmoud Eman Mohammed, Elfadel Magboul Nisrin Magboul. Artificial Intelligence Applications in Obstetric Risk Prediction: A Systematic Review of Machine Learning Models for Preeclampsia. Cureus. 2025;17(5):12. Located at: ProQuest Central; 3225671464. doi:10.7759/cureus.83961

323. Tun HM, Rahman HA, Naing L, Malik OA. Trust in Artificial Intelligence-Based Clinical Decision Support Systems Among Health Care Workers: Systematic Review. J Med Internet Res. 2025 Jul 29;27:e69678. doi:10.2196/69678 PubMed PMID: 40772775; PubMed Central PMCID: PMC12440830.

324. Enhancing clinical decision support with explainable deep learning framework for C-section forecasting. Computing Archives for Informatics and Numerical Computation. 2025 Jan;107(1):20. Located at: ProQuest Central; 3141981787. doi:10.1007/s00607-024-01354-2

325. Nguyen RN, Lam HT, Phan HV, Bui NQ. Machine Learning Nomogram for Predicting Dengue Shock Syndrome in Pediatric Patients With Dengue Fever in Vietnam. Cureus. 2025;17(4). Located at: ProQuest Central; 3204699633. doi:10.7759/cureus.81819

326. Oluwatobi Idowu Dr, Aderinto N Dr, Gbolahan Olatunji Dr, Emmanuel Kokori Dr. Machine Learning in Schizophrenia: A Systematic Review and Meta-Analysis of Diagnostic and Predictive Models. BJPsych Open. 2025 Jun;11(S1):S44. Located at: ProQuest Central; 3232683921. doi:10.1192/bjo.2025.10148

327. Petsiou DP, Spinos D, Martinos A, Muzaffar J, Garas G, Georgalas C. Effectiveness of Artificial Intelligence in detecting sinonasal pathology using clinical imaging modalities: a systematic review. Rhinology. 2025 Aug 1;63(4):448–62. doi:10.4193/Rhin25.044 PubMed PMID: 40388840.

328. Rao A, Haydel J, Ma S, Thrift AP, Nguyen-Wenker T, El-Serag HB. A Simple, Interpretable Machine Learning Model Based on Clinical Factors Accurately Predicts Incident Dysplasia or Malignancy in Barrett’s Esophagus. Digestive Diseases and Sciences. 2025 Aug;70(8):2739–49. Located at: ProQuest Central; 3241416291. doi:10.1007/s10620-025-09069-w

329. Ren B, Zhang Y, Chen S, Dai J, Chong J, Zhong Y, et al. Interpretable prediction of hospital mortality in bleeding critically ill patients based on machine learning and SHAP. BMC Medical Informatics and Decision Making. 2025;25:1–12. Located at: ProQuest Central; 3236995390. doi:10.1186/s12911-025-03101-9

330. Narvaez RA, Ferrer M, Peco RA, Mejilla J. Artificial intelligence in symptom management and clinical decision support for palliative care. Int J Palliat Nurs. 2025 Jun 2;31(6):294–306. doi:10.12968/ijpn.2025.0041

331. Sharma G, Yaffe MJ, Ghadiri P, Gandhi R, Pinkham L, Gore G, et al. Use of Artificial Intelligence in Adolescents’ Mental Health Care: Systematic Scoping Review of Current Applications and Future Directions. JMIR Ment Health. 2025 Jun 6;12:e70438–e70438. doi:10.2196/70438

332. Shrikrishna BH, Deepa G. The Application and Diagnostic Accuracy of Artificial Intelligence in Rhinology: A Review. Cureus. 2025;17(7):10. Located at: ProQuest Central; 3244982505. doi:10.7759/cureus.87966

333. Soriano-Arandes Antoni, Andrés C, Perramon-Malavez Aida, Creus-Costa A, Gatell A, Martín-Martín Ramona, et al. Implementing Symptom-Based Predictive Models for Early Diagnosis of Pediatric Respiratory Viral Infections. Viruses. 2025;17(4):546. Located at: Coronavirus Research Database; ProQuest Central; 3194648518. doi:10.3390/v17040546

334. Stuke H, Schlack R, Erhart M, Kaman A, Ravens-Sieberer U, Irrgang C. Peer Relationships Are a Direct Cause of the Adolescent Mental Health Crisis: Interpretable Machine Learning Analysis of 2 Large Cohort Studies. JMIR Public Health and Surveillance. 2025;11. Located at: Coronavirus Research Database; ProQuest Central; 3206921765. doi:10.2196/60125

335. Tomassini S, Duranti D, Zeggada A, Cosimo Quattrocchi C, Melgani F, Giorgini P. Multi-Branch CNN-LSTM Fusion Network-Driven System With BERT Semantic Evaluator for Radiology Reporting in Emergency Head CTs. IEEE J Transl Eng Health Med. 2025;13:61–74. doi:10.1109/JTEHM.2025.3535676

336. Vali M, Hossein Motahari Nezhad, Kovacs L, Gandomi AH. Machine learning algorithms for predicting PTSD: a systematic review and meta-analysis. BMC Medical Informatics and Decision Making. 2025;25:1–16. Located at: Coronavirus Research Database; ProQuest Central; 3165425967. doi:10.1186/s12911-024-02754-2

337. Wang L, Zhang S, Gao Z, Jiang D. Construction and validation of a risk prediction model for chronic obstructive pulmonary disease (COPD): a cross-sectional study based on the NHANES database from 2009 to 2018. BMC Pulm Med. 2025 Jul 3;25(1):317. doi:10.1186/s12890-025-03776-w PubMed PMID: 40611010; PubMed Central PMCID: PMC12225070.

338. Wang X, Zhang P, Lu H, Luo D, Yang D, Kang L, et al. Risk prediction models for dental caries in children and adolescents: a systematic review and meta-analysis. BMJ Open. 2025;15(3). Located at: ProQuest Central; 3176352304. doi:10.1136/bmjopen-2024-088253

339. Xiong X, Fu H, Xu B, Wang W, Zhou M, Hu P, et al. Ten Machine Learning Models for Predicting Preoperative and Postoperative Coagulopathy in Patients With Trauma: Multicenter Cohort Study. Journal of Medical Internet Research. 2025;27. Located at: ProQuest Central; 3222368279. doi:10.2196/66612

340. Yang X, Zhang Y, Li Y, Wu Z. Performance of Artificial Intelligence in Diagnosing Lumbar Spinal Stenosis: A Systematic Review and Meta-Analysis. Spine (Phila Pa 1976). 2025 May 15;50(10):E179–96. doi:10.1097/BRS.0000000000005174 PubMed PMID: 39451133.

341. Yuan S, Yang Z, Li J, Wu C, Liu S. AI-Powered early warning systems for clinical deterioration significantly improve patient outcomes: a meta-analysis. BMC Medical Informatics and Decision Making. 2025;25:1–8. Located at: Coronavirus Research Database; ProQuest Central; 3216558206. doi:10.1186/s12911-025-03048-x

342. Zhang J, Zhu W, Jiang P, Ma F, Li Y, Cao Y, et al. In-depth analysis of the risk factors for persistent severe acute respiratory syndrome coronavirus 2 infection and construction of predictive models: an exploratory research study. BMC Infectious Diseases. 2025;25:1–18. Located at: Coronavirus Research Database; ProQuest Central; 3216558387. doi:10.1186/s12879-025-11083-2

343. Zmudzki F, Rob J E M Smeets, Groenewegen JS, van der Graaff E. Machine Learning Clinical Decision Support for Interdisciplinary Multimodal Chronic Musculoskeletal Pain Treatment: Prospective Pilot Study of Patient Assessment and Prognostic Profile Validation. JMIR Rehabilitation and Assistive Technologies. 2025;12. Located at: ProQuest Central; 3206910829. doi:10.2196/65890

344. Lekadir K, Frangi AF, Porras AR, Glocker B, Cintas C, Langlotz CP, et al. FUTURE-AI: international consensus guideline for trustworthy and deployable artificial intelligence in healthcare. BMJ. 2025 Feb 5;388:e081554. doi:10.1136/bmj-2024-081554

345. Raghunathan K, Morris ME, Wani TA, Edvardsson K, Peiris C, Fowler-Davis S, et al. Using artificial intelligence to improve healthcare delivery in select allied health disciplines: a scoping review protocol. BMJ Open. 2025 Mar;15(3):e098290. doi:10.1136/bmjopen-2024-098290

346. Petrella F, Rizzo S. Artificial Intelligence in Oncologic Thoracic Surgery: Clinical Decision Support and Emerging Applications. Cancers. 2026;18(2):246. Located at: ProQuest Central; 3297397967. doi:10.3390/cancers18020246
